# Effects of Formal Metal Oxidation State on the Preferred Structure Types in Binuclear Actinide Carbonyl Derivatives: Predicted Tetramerization of Carbon Monoxide to a Bridging Squarate Group in Uranium Chemistry

**DOI:** 10.1021/acs.jpca.5c07951

**Published:** 2026-02-27

**Authors:** Amr A. A. Attia, Alexandru Lupan, R. Bruce King

**Affiliations:** † Faculty of Chemistry and Chemical Engineering, Babeş-Bolyai University, Cluj-Napoca RO-400028, Romania; ‡ Department of Chemistry, University of Georgia, Athens, Georgia 30602, United States

## Abstract

The structures and energetics of the binuclear cyclooctatetraene uranium carbonyls (C_8_H_8_)_2_U_2_(CO)_*n*_ (*n* = 2, 3, 4, 5) have been studied by density functional theory. The most interesting observation from this work is the prediction of low-energy structures in the tetracarbonyl system of the type (C_8_H_8_)_2_U_2_(η^4^-μ-C_4_O_4_), in which the four CO groups couple to form a bridging C_4_O_4_ squarate unit. Such a tetramerization of carbon monoxide to give a squarate unit by organouranium compounds has been observed experimentally by Cloke and co-workers in sandwich compounds of the type (η^5^-Me_5_C_5_)­U­(η^8^-C_8_H_6_{SiR_3_}_2_) containing both five-membered and eight-membered rings. However, tetramerizations of CO groups to squarate were not predicted in theoretical studies of related (C_8_H_8_)_2_Th_2_(CO)_4_ or (C_5_H_5_)_2_M_2_(CO)_4_ systems (M = Th, U). These bridging squarate (C_8_H_8_)_2_U_2_(η^4^-μ-C_4_O_4_) structures found in this work are thermochemically favored to the extent that the lowest energy structure of the tricarbonyl (C_8_H_8_)_2_U_2_(CO)_3_ is disfavored relative to disproportionation into such a bridging squarate tetracarbonyl structure and the lowest energy structure of the dicarbonyl (C_8_H_8_)_2_U_2_(CO)_2_. In the remaining low-energy (C_8_H_8_)_2_U_2_(CO)_*n*_ (*n* = 2, 3, 4, 5) structures, the carbonyl groups are all isolated, either as terminal CO groups similar to those bonding to d-block metals or as bridging η^2^-μ-CO groups bonded to uranium through both their carbon and oxygen atoms. The viability of formal uranium oxidation states from +3 to +6, as found experimentally in diverse stable molecules, leads to a variety of spin states and uranium–uranium bonding modes in the low-energy (C_8_H_8_)_2_U_2_(CO)_*n*_ (*n* = 2, 3, 4, 5) structures. This contrasts with the previously studied thorium systems (C_8_H_8_)_2_Th_2_(CO)_*n*_ (*n* = 2, 3, 4, 5)^6^, where the maximum viable formal thorium oxidation state of +4 limits the range of accessible structure types, metal–metal bonding modes, and spin states.

## Graphical abstract

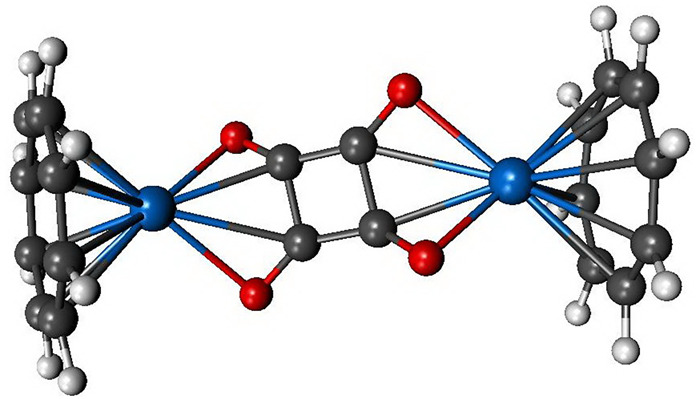

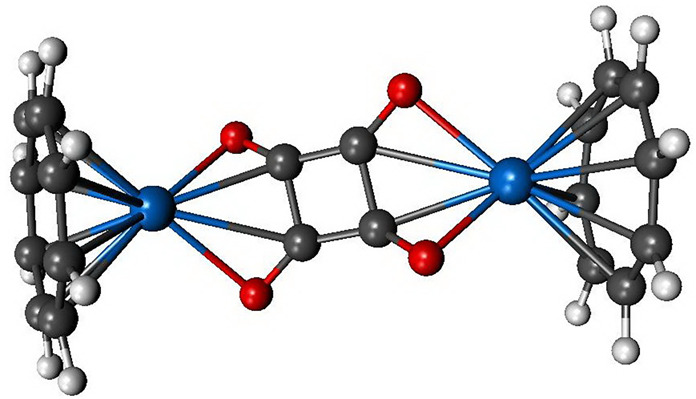

## 1 Introduction

The richness of metal carbonyl chemistry of the d-block transition metals, as exemplified by stable binary metal carbonyls such as Ni­(CO)_4_, Fe­(CO)_5_, and M­(CO)_6_ (M = Cr, Mo, W),^1–3^ is not matched by that of the f-block metals such as the lanthanides and actinides. Initially this could seem rather surprising since both d-block and f-block transition metals have available d-orbitals for the dπ→pπ* back-bonding to carbonyl groups coordinated to the central metal atom(s) solely through the carbon atom. However, a major factor limiting the scope of metal carbonyl chemistry of the f-block metals is their high affinity for oxygen. This leads to preferred structures in which carbonyl groups are bonded to the metals not only through their carbon atoms but also through their oxygen atoms. This high affinity of the f-block metals for bonding to oxygen is supported by our theoretical studies on the binuclear derivatives Cp_2_Ln_2_(CO)_*n*_ (Ln = La, Lu; Cp = η^5^-C_5_H_5_),^4^ Cp_2_Th_2_(CO)_*n*_,^5^ (C_8_H_8_)_2_Th_2_(CO)_*n*_,^6^ and Cp_2_U_2_(CO)_*n*_.^7^ In most of the low-energy structures found in all of these binuclear systems, at least two carbonyl groups are bridging η^2^-μ-CO groups that are bonded to a metal atom not only through their carbon atoms but also through their oxygen atoms. In addition, a number of low-energy structures were found in which two carbonyl groups couple to form a bridging C_2_O_2_ unit that uses both of its oxygen atoms for bonding to the central M_2_ unit. In addition to a central MC_*n*_O_*n*_M unit in a Cp_2_M_2_(CO)_*n*_ or (C_8_H_8_)_2_M_2_(CO)_*n*_ derivative, terminal CO groups are predicted for some of the carbonyl richer structures. Such terminal CO groups in f-block metal carbonyl derivatives are predicted to exhibit ν­(CO) frequencies in a similar region to that of similar terminal CO groups in related d-block metal derivatives, typically above 1800 cm^–1^.

Experimentally known viable carbonyl derivatives of the f-block metals stable under ambient conditions are very limited. Several mononuclear substituted triscyclopentadienyluranium carbonyls of the type Cp_3_U­(CO) are known in which Cp corresponds to a suitably substituted pentahapto cyclopentadienyl ligand.^8–11^ The steric requirements of three such Cp ligands around a single uranium atom restrict the available space for a fourth ligand. Because of the resulting blocking effect of the uranium coordination sphere by three such ligands, addition of a carbonyl group to a Cp_3_U system to form a Cp_3_U­(CO) derivative leads to a linearly coordinated CO group bonded to the metal atom through only its carbon atom rather than the more sterically demanding laterally bonding mode involving coordination of both its carbon and oxygen atoms. More recently the stable uranium carbonyl sterically hindered aryloxy derivative Cp*_2_U­(OC_6_H_2_Bu^*t*^_2_Me)­(CO) (Cp* = η^5^-Me_5_C_5_) has been synthesized and structurally characterized by X-ray crystallography.^12^ The related Cp*_2_U­(As_2_Mes_2_)­(CO) (Mes = mesityl) has also been prepared in solution and characterized spectroscopically.^13^ However, it was only stable in a CO atmosphere and thus could not be structurally characterized. The binary actinide metal carbonyls M­(CO)_*n*_ (M = Th, *n* = 1–6 ^14,15^ ; M = U, *n* = 1, 2, 6 ^16^) have been observed at low temperatures in reactions of laser ablated metal atoms with carbon monoxide. However, they are not stable under normal laboratory conditions.

Carbonyl groups as well as their C_2_O_2_ dimers bridging central M_2_ units of f-block metals by forming both U–C and U–O bonds can be considered as dianions formed by deprotonation of simple molecules. Thus, a bridging η^2^-μ-CO group can be considered as a CO^2–^ dianion derived from double deprotonation of formaldehyde, HC­(O)­H, or its C­(OH)_2_ tautomer. Similarly, a bridging η^4^-μ-C_2_O_2_ group can be considered as a C_2_O_2_^4–^ tetraanion derived from a quadruple deprotonation of acetaldehyde, CH_3_C­(O)­H, or its enol tautomer vinyl alcohol, CH_2_=CHOH. Such interpretations can be very helpful in accounting for the lowest energy binuclear lanthanide and thorium Cp_2_M_2_(CO)_*n*_ and (C_8_H_8_)_2_M_2_(CO)_*n*_ structures since lanthanides have a strongly preferred +3 oxidation state and thorium has a strongly preferred +4 oxidation state (Figure 1 and Table 1). Thus, a typical central structural unit in Cp_2_Ln_2_(CO)_*n*_ derivatives is of the type Ln^III^_2_(η^2^-μ-C_2_O_2_) with the favorable +3 lanthanide oxidation state after considering the uninegative Cp^–^ anions bonded to each lanthanide. Similarly, a typical central structural unit in (C_8_H_8_)_2_Th_2_(CO)_*n*_ derivatives is of the type Th^IV^_2_(η^2^-μ-C_2_O_2_) after considering the dinegative C_8_H_8_^2–^ dianion bonded to each thorium atom. For the Cp_2_Th_2_(CO)_*n*_ systems a central triply bridging Th^IV^_2_(η^2^-μ-CO)_3_ unit leads to the favorable +4 oxidation state for each thorium atom. Cp_2_Th_2_(CO)_*n*_ systems with only two bridging η^2^-μ-CO units correspond to the d^1^ or f^1^ Th­(III) oxidation state suggesting the possibility of a Th–Th single bond in such systems. Note, however, that the rigid geometry of the η^2^-μ-CO or η^4^-μ-C_2_O_2_ bridges across a central M_2_ unit can restrict severely the range of metal–metal bond distances. This complicates any relationship between such distances and formal metal–metal bond order. Mayer Bond Orders (MBO) appear to be closer to the apparent formal metal–metal bond orders deduced from other considerations than the Wiberg Bond Indices (WBI).^17^

**Table 1:** Formal f-Block Metal Oxidation States in Cp_2_M_2_(CO)_*n*_ and (C_8_H_8_)_2_M_2_(CO)_*n*_ Derivatives and the Nature of Their Metal–Metal Bonds

central framework	dissection	M–M bonding	literature
Cp_2_Ln_2_(η^4^-μ-C_2_O_2_)	2Ln^3+^+2Cp^–^ + η^4^-μ-C_2_O_2_^4–^	none	^4^
Cp_2_Th_2_(η^2^-μ-CO)_2_	2Th^3+^ + 2Cp^–^ + 2η^2^-μ-CO	Th–Th ∼ 3.4 Å	^5^
Cp_2_Th_2_(η^2^-μ-CO)_3_	2Th^4+^ + 2Cp^–^ + 3η^2^-μ-CO	none (Th···Th ∼ 3.7 Å)	^5^
(η^8^-C_8_H_8_)_2_Th_2_(η^4^-μ-C_2_O_2_)	2Th^4+^ + 2C_8_H_8_^2–^ + η^4^-μ-C_2_O_2_^4–^	none	^6^
Cp_2_U_2_(η^2^-μ-CO)_2_	2U^3+^ + 2Cp^2^ + 2η^2^-μ-CO	U≡U ∼ 2.4 Å	^7^
Cp_2_U_2_(η^2^-μ-CO)_3_	2U^4+^ + 2Cp^2^ + 3η^2^-μ-CO	U=U ∼ 2.45 Å	^7^
Cp_2_U_2_(η^2^-μ-CO)_4_	2U^5+^ + 2Cp^2^ + 4η^2^-μ-CO	U–U ∼ 3.4 Å	^7^
(η°-C_8_H_8_)_2_U_2_(η^2^-CO)_2_	2U^4+^ + 2C_8_H_8_^2–^ + 2η^2^-μ-CO	U=U ∼ 2.5 Å	this work

**Figure 1:**
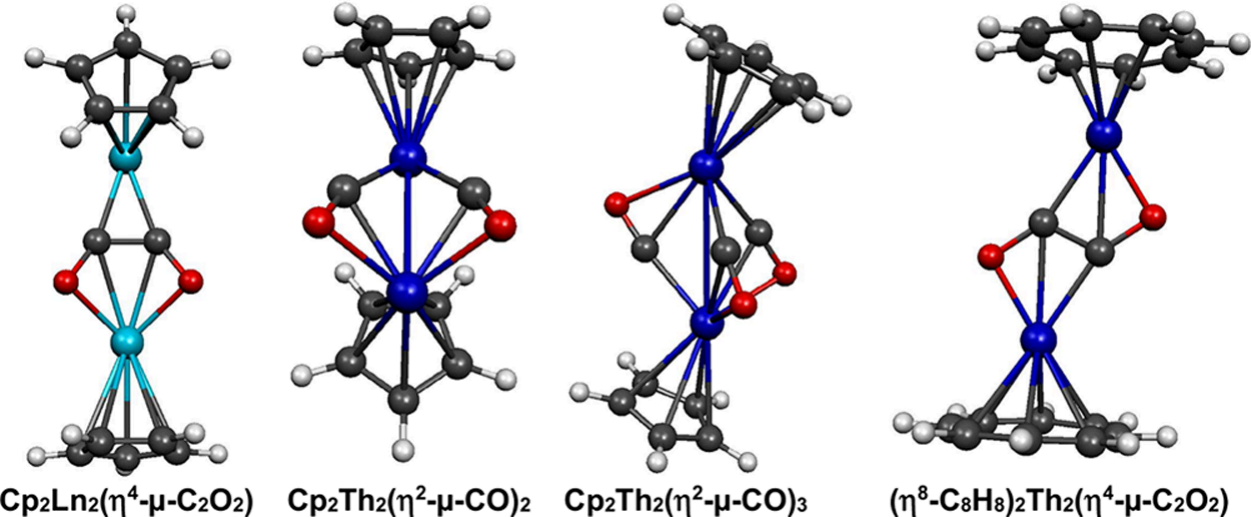
Sample structures of the binuclear lanthanide and thorium carbonyl complexes listed in Table 1.

The metal–metal bonding situation with the f-block metal uranium in its binuclear derivatives is significantly more complicated than that with the lanthanides and thorium because of the variety of reasonably favorable uranium oxidation states ranging from +3 to +6. For any uranium formal oxidation state below +6 in a binuclear derivative, electrons remain on each uranium atom to provide opportunities for diverse types of uranium–uranium bonding. The complexity of such uranium–uranium bonding is illustrated by the unusual bonding in the bare uranium dimer U_2_. Thus, the formal quintuple bond in U_2_ consists of a normal σ + 2π triple bond similar to the C≡C triple bond in acetylene, supplemented by four single-electron “half bonds”.^18,19^ This, coupled with two unpaired nonbonding electrons leads to a septet ground state for U_2_. Furthermore, a computational study on the permethylated “diuranocene,” (η^5^-Me_5_C_5_)_2_U_2_, suggests an unusual σ + ^2^/_2_π + ^2^/_2_δ net triple U≡U bond consisting of a full σ-bond supplemented by four one-electron “half-bonds” with π and δ symmetries.^20^ Such a formal U≡U triple bond with four unpaired electrons within the bond corresponds to a formal f^2^ U­(IV) oxidation state with two additional unpaired electrons on each uranium atom. The total of eight unpaired electrons predicted for (η^5^-Me_5_C_5_)_2_U_2_ corresponds to an unusually high spin nonet ground state for the molecule.

Experimental work on interaction of carbon monoxide with organouranium systems provides examples of coupling of several CO units to give uranium complexes containing oxocarbon ligands^21^ such as ethynediolate (C_2_O_2_ ^2^),^22^ deltate (*cyclo*-C_3_O_3_^2–^),^23^ and squarate (*cyclo*-C_4_O_4_^2–^).^24^ Thus, two types of formal uranium­(III) derivatives, namely sandwich compounds (η^5^-Me_5_C_5_)­U­(η^8^-C_8_H_6_{SiR_3_}_2_) containing both cyclopentadienyl and cyclooctatetraene ligands as well as the uranium­(III) tris­(amide) complex^25^ [(Me_3_Si)_2_N]_3_U, promote the reductive dimerization of CO to give complexes with a coordinated ethynediolate ligand. For the uranium sandwich compounds, the extent of CO coupling appears to depend on the steric demands of the substituents on the cyclooctatetraene ring. The mechanism of CO dimerization on the mixed sandwich system is suggested by computational studies to involve a binuclear uranium intermediate containing a bridging ethynediolate group.^26^

A variety of binuclear uranium carbonyl systems of the general type (C_*x*_H_*x*_)­(C_*y*_H_*y*_)­U_2_(CO)_*n*_ containing various combinations of the anionic planar hydrocarbon ligands C_5_H_5_^–^ (Cp^–^), C_7_H_7_^3–^, and C_8_H_8_^2–^ with either 6 or 10 π-electrons conforming to the Hückel 4*n* + 2 rule are of potential interest. The aggregate negative charge provided by these hydrocarbon ligands in their (C_*x*_H_*x*_)­(C_*y*_H_*y*_)­U_2_(CO)_*n*_ complexes is anticipated to have a major effect on the nature of their carbonyl groups in the lowest energy structures. For our initial studies in this area, we chose the Cp_2_U_2_(CO)_*n*_ systems containing two monoanionic Cp^–^ ligands so that the uranium atoms have an average formal U­(I) oxidation state if the carbonyl ligands are neutral ligands. Such carbonyl groups are similar to the CO groups bonded only through carbon atoms found in almost all carbonyl derivatives of the d-block metals. Numerous cyclopentadienyluranium derivatives are known that could be the precursors of the synthesis of such molecules. The low formal U­(I) oxidation states in Cp_2_U_2_(CO)_*n*_ complexes might be expected to lead to reduction of some of the CO groups to formally dianionic bridging η^2^-μ-CO groups or reductive coupling to tetraanionic C_2_O_2_^4–^ groups. However, no examples of CO reductive coupling to form C_2_O_2_^4–^ tetraanionic ligands were found in the lowest energy structures.^7^ In this way the uranium Cp_2_U_2_(CO)_*n*_ systems differ significantly from the analogous thorium systems Cp_2_Th_2_(CO)_*n*_ where a number of examples of reductive coupling to form C_2_O_2_^4–^ ligands were predicted.^5^ The retention of uranium valence electrons not involved in metal–ligand bonding in the low-energy Cp_2_U_2_(CO)_*n*_ (*n* = 3, 4) structures, even after reduction of CO to η^4^-μ-CO^2–^ or η^4^-μ-C_2_O_2_^4–^, was found to lead to a complicated variety of predicted U–U bonding modes including formal multiple bonds containing unpaired electrons in single-electron half-bond components. This leads to spin states in low-energy structures ranging from singlet to quintet.

We now report a study of (C_8_H_8_)_2_U_2_(CO)_*n*_ complexes containing two dianionic C_8_H_8_^2–^ ligands so that each uranium atom has the formal U­(II) oxidation state if all of the CO groups are neutral terminal ligands bonded exclusively through their carbon atoms. This change in the hydrocarbon ligands is expected to have a profound effect in the energetically preferred structures. The most striking finding from this study is the presence of a bridging squarate tetraanion C_4_O_4_^4–^ in the lowest energy structures of the tetracarbonyl (C_8_H_8_)_2_U_2_(CO)_4_ arising from the tetramerization of carbon monoxide. The long U···U distances in these bridging squarate structures combined with the quintet spin state of the two lowest energy (C_8_H_8_)_2_U_2_(η^4^-μ-C_4_O_4_) indicate that these are high-spin f^2^ U­(IV) complexes. A higher energy low-spin singlet bridging squarate (C_8_H_8_)_2_U_2_(η^4^-μ-C_4_O_4_) complex was also found. This prediction of low-energy bridging squarate structures in the (C_8_H_8_)_2_U_2_(CO)_4_ system is not totally surprising because of the experimental observation of tetramerization of carbon monoxide to squarate observed by Cloke and co-workers in related organouranium sandwich compounds.^24^

However, this tetramerization of carbon monoxide was the only example of coupling of carbonyl groups found in the entire series of (C_8_H_8_)_2_U_2_(CO)_*n*_ (*n* = 2, 3, 4, 5) found in this work. The carbonyl groups in all of the other lowest energy such structures are either bridging η^2^μ-CO groups or terminal CO groups using only their carbon atoms for bonding to a uranium atom similar to the terminal CO groups in the ubiquitous d-block transition metal carbonyl derivatives.

## 2 Theoretical Methods

The initial (C_8_H_8_)_2_U_2_(CO)_*n*_ (*n* = 2, 3, 4, 5) structures were constructed by systematic placement of CO molecules as terminal and bridging ligands coordinating through only the carbon atom or through both the carbon and oxygen atoms. This led to 19 different starting structures for (C_8_H_8_)_2_U_2_(CO)_2_, 71 starting structures for (C_8_H_8_)_2_U_2_(CO)_3_, 104 starting structures for (C_8_H_8_)_2_U_2_(CO)_4_ and 189 starting structures for (C_8_H_8_)_2_U_2_(CO)_5_. All structures were optimized as singlets, triplets, quintets, and septets.

Full geometry optimizations were carried out on the (C_8_H_8_)_2_U_2_(CO)_*n*_ systems by using the BP86 DFT functional coupled with the def2-TZVP basis set for all atoms except uranium for which the ECP60MWB basis set including pseudopotentials was used.^27–33^ The nature of the stationary points after optimization was checked by calculations of the harmonic vibrational frequencies. If significant imaginary frequencies were found, the optimization was continued by following the corresponding normal modes to ensure that genuine minima were obtained. In addition, single point energy calculations were performed on all optimized structures by utilizing the BP86 DFT functional and the def2-TZVP basis set coupled with the zero-order regular approximation (ZORA)^34^ as implemented in the ORCA 3.0.3 software package.^35^ These calculations were performed using very tight convergence criteria; the resulting energetics are discussed in the text. Single point calculations of the relative energies of the structures optimized by the PB86 method were performed using the M06L/def2-TZVP/ZORA method and are listed in the tables.

All geometry optimizations were performed using the Gaussian 09 package^36^ with the default settings for the SCF cycles and geometry optimization. Wiberg bond indices (WBIs) for the U–U interactions in the optimized (C_8_H_8_)_2_U_2_(CO)_*n*_ structures were determined using NBO analysis^37^ since they are well-established as means for evaluating M–M interactions. In addition, U–U Mayer bond order values (MBOs)^17^ were also calculated since they were often found to be more reliable than WBIs for many inorganic systems. Indeed for a number of the compounds discussed in this paper, their MBOs were found to give more reasonable values than their WBIs. The structures, total and relative energies, and relevant interatomic distances for all of the optimized structures are given in the Supporting Information. Structures in the main text are designated as **C8U2COn-xA** where **n** refers to the number of CO groups, **A** refers to the spin state with **S**, **T**, and **Q** corresponding to singlet, triplet, and quintet spin states, respectively, and **x** orders the structures according to their relative energies by the BP86 method. Only the lowest energy and thus potentially chemically significant structures are considered in detail in this paper. However, more comprehensive lists of structures, including higher energy structures, are given in the Supporting Information.

## 3 Results and Discussion

### 3.1 The (C_8_H_8_)_2_U_2_(CO)_4_ Tetracarbonyl System Including Bridging Squarate Structures

Seven (C_8_H_8_)_2_U_2_(CO)_4_ tetracarbonyl structures were found within 13 kcal/mol of the lowest energy structure **C8U2CO4-1Q** using the BP86 method (Figure 2 and Table 2). However, single point calculations of the energies of these seven optimized (C_8_H_8_)_2_U_2_(CO)_4_ structures using the M06L/def2-TZVP/ZORA method showed the three quintet structures to have far lower energies than any of the other four (C_8_H_8_)_2_U_2_(CO)_4_ structures. Thus, using this M06L method gave an energy of the highest energy of the three quintet (C_8_H_8_)_2_U_2_(CO)_4_ structures, namely **C8U2CO4-5Q**, more than 10 kcal/mol below the lowest energy lower spin structure, namely the singlet structure **C8U2CO4-1S**.

**Figure 2:**
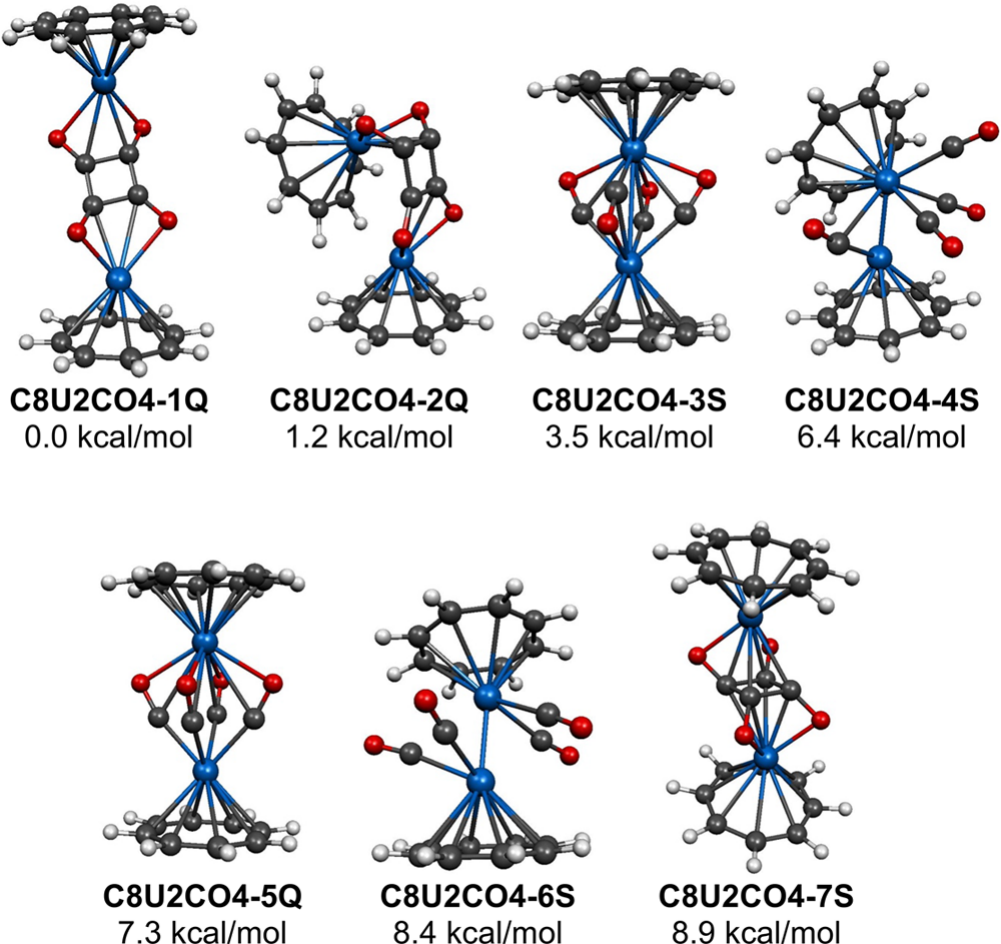
Seven (C_8_H_8_)_2_U_2_(CO)_4_ structures within 13 kcal/mol of energy.

**Table 2:** Seven (C_8_H_8_)_2_U_2_(CO)_4_ Structures within 13 kcal/mol of Energy by the BP86 Method with Δ*E* in kcal/mol, Bond Lengths in Å, and ν­(CO) Frequencies in cm^–1^

	Δ*E*		U–U bonding				bridging CO groups	
structure	BP86	M06L	length	WBI	MBO	order	type	ν(CO)
**C8U2CO4-1Q**	0.0	0.4	5.358	0.08	0.09	0	η^4^-μ-C_4_O_4_	1289, 1328, 1495, 1631
**C8U2CO4-2Q**	1.2	0.0	4.021	0.24	0.27	0	η^4^-μ-C_4_O_4_	1296, 1377, 1407, 1547
**C8U2CO4-3S**	3.5	22.0	3.460	1.52	1.07	1	4η^2^-μ-CO	1464, 1542, 1561, 1653
**C8U2CO4-4S**	6.4	25.0	2.480	3.66	2.12	2	all terminal CO groups	
**C8U2CO4-5Q**	7.3	11.1	4.004	0.42	0.52	0	4η^2^-μ-CO	1566, 1572, 1587, 1658
**C8U2CO4-6S**	8.4	29.2	2.536	3.54	1.80	2	all terminal CO groups	
**C8U2CO4-7S**	8.9	30.7	4.598	1.10	1.12	1	η^4,4^-μ-C_4_O_4_	1201, 1214, 1221, 1299

The two lowest-energy (C_8_H_8_)_2_U_2_(CO)_4_ structures, namely the energetically very closely spaced quintet structures **C8U2CO4-1Q** and **C8U2CO4-2Q** within 1.5 kcal/mol, have all four of their CO groups coupled through their carbon atoms to form a C_4_ cyclobutane ring leading to a bridging squarate unit (Figure 2 and Table 2). The long U···U distances in **C8U2CO4-1Q** and **C8U2CO4-2Q** as well as their near-zero WBI and MBO values indicate the lack of a formal uranium–uranium bond. The resulting μ-C_4_O_4_ unit in **C8U2CO4-1Q** is bonded to the uranium atoms through only its oxygen atoms. Considering the bridging C_4_O_4_^4–^ unit as a squarate tetraanion leads to a formal f^2^ U­(IV) uranium oxidation state after considering the C_8_H_8_^2–^ rings as the usual dianions. The quartet spin state of **C8U2CO41Q** implies high-spin U­(IV) for both uranium atoms with no pairing of the f^2^ electrons on either uranium atom. In the other low-energy quartet (C_8_H_8_)_2_U_2_(CO)_4_ squarate structure **C8U2CO4-2Q** one of the uranium atoms is bonded to the bridging squarate group through not only two oxygen atoms but also two carbon atoms. However, the other uranium atom in **C8U2CO4-2Q** is bonded to the bridging squarate group only through two of its oxygen atoms. If the bonding of one uranium atom in **C8U2CO4-2Q** to two adjacent carbon atoms in the squarate ring is regarded as the analogue of an olefin bonded to a d-block metal through its C=C double bond, then these U–C bonds in **C8U2CO4-2Q** do not affect the formal uranium oxidation state. The addition of two U–C bonds in going from **C8U2CO4-1Q** to **C8U2CO4-2Q** has a drastic effect on the stereochemistry in order to place two carbon atoms in the C_4_O_4_ unit within bonding distance of the uranium atom.

Two (C_8_H_8_)_2_U_2_(CO)_4_ structures were found in which the four CO groups form separate bridging η^2^-μ-CO groups rather than tetramerize into a squarate ring (Figure 2 and Table 2), namely the singlet structure **C8U2CO4-3S** and the quintet structure **C8U2CO4-5Q** lying 3.5 and 7.3 kcal/mol (BP86), respectively, in energy above **C8U2CO4-1Q** (Figure 2 and Table 2). In the singlet structure **C8U2CO4-3S**, three of the η^2^-μ-CO groups are oriented in one direction with the fourth such group oriented in the opposite direction. All of the CO groups are bonded to uranium atoms through both their carbon and oxygen atoms. Considering each of the η^2^-μ-CO^2–^ groups as a dianions leads to the favorable f^0^ U­(VI) oxidation state corresponding to the observed singlet spin state. These η^2^-μ-CO^2–^ groups are predicted to exhibit very low ν­(CO) frequencies ranging from 1464 to 1568 cm^–1^ as compared with typical ν­(CO) terminal and bridging μ-CO groups in d-block transition metal chemistry. This relates to the extreme back-bonding leading to their formal dianionic nature. The geometry of the four η^2^-μ-CO^2–^ bridges each bonded to uranium through both their carbon and oxygen atom limits the U···U separation to an apparent maximum of ∼3.46 Å, which is close enough for some type of interaction as indicated by WBI and MBO values around 1.

The situation is very different for the quintet (C_8_H_8_)_2_U_2_(CO)_4_ structure **C8U2CO4-5Q** with all four bridging η^2^-μ-CO groups oriented in the same direction so that one uranium atom is bonded only to carbonyl carbon atoms and the other uranium is bonded only to carbonyl oxygen atoms (Figure 2 and Table 2). The high spin state of **C8U2CO4-5Q** is consistent with its η^2^-μ-CO groups being neutral rather than dianionic ligands so that the formal uranium oxidation state is U­(II) rather than U­(VI). In **C8U2CO4-5Q** the uranium atom bonded to the four carbonyl oxygen atoms has a spin density of 2.24 whereas the other uranium atom has a spin density of 1.28. The remaining spin density of the quintet structure is distributed approximately equally among the four carbonyl groups.

The remaining two of the seven lowest energy (C_8_H_8_)_2_U_2_(CO)_4_ structures, namely the singlet **C8U2CO4-4S** and **C8U2CO4-6S** structures lying 6.4 and 8.4 kcal/mol (BP86) above **C8U2CO4-1Q** by the BP86 method, have exclusively terminal CO groups (Figure 2 and Table 2). Each of these two structures has a short unbridged U=U distance of ∼2.5 Å suggested by its MBO value of ∼2 to be a formal double bond. These bond lengths are somewhat shorter than the 2 × 1.34 = 2.68 Å length suggested by twice the U=U double bond radius.^38^ In **C8U2CO4-4S** the terminal CO groups are unevenly divided so that one uranium atom bears three CO groups whereas the other uranium atom bears only a single CO group. This contrasts with **C8U2CO4-6S** in which each uranium atom bears two CO groups.

### 3.2 The (C_8_H_8_)_2_U_2_(CO)_2_ Dicarbonyl System

Four (C_8_H_8_)_2_U_2_(CO)_2_ dicarbonyl structures were found within energies of 14 kcal/mol above the lowest energy structure **C8U2CO2-1T** (Figure 3 and Table 3). All four structures have two separate bridging η^2^-μ-CO groups. These two η^2^-μ-CO groups in each of these four (C_8_H_8_)_2_U_2_(CO)_2_ structures exhibit two ν­(CO) frequencies ranging from 1494 to 1609 cm^–1^, which are considerably below those found for either terminal or bridging carbonyl groups bonded to metals solely through their carbon atoms. The two lowest energy such (C_8_H_8_)_2_U_2_(CO)_2_ structures **C8U2CO2-1T** and **C8U2CO2-2T** are triplet structures with distinctly different stereochemistries. The next two structures **C8U2CO2-3S** and **C8U2CO2-4S**, lying 6.6 and 9.8 kcal/mol (BP86), respectively, above **C8U2CO2-1T** are singlet structures with stereochemistries similar to the triplet structures **C8U2CO2-2T** and **C8U2CO2-1T**, respectively. The single point calculations using the M06L functional agree well within experimental error with those obtained by the BP86 method.

**Figure 3:**
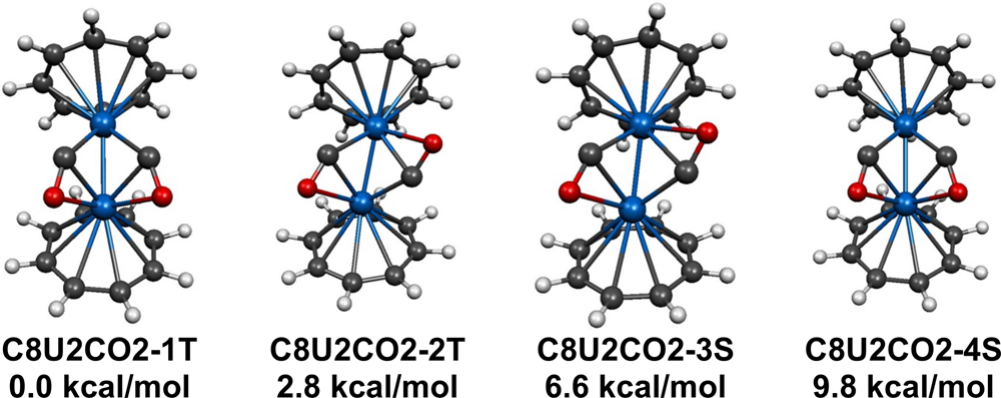
Four (C_8_H_8_)_2_U_2_(CO)_2_ structures within 14 kcal/mol of energy.

**Table 3:** Four (C_8_H_8_)_2_U_2_(CO)_2_ Structures within 14 kcal/mol of Energy with Δ*E* in kcal/mol, Bond Lengths in Å, and ν­(CO) Frequencies in cm^–1^

	Δ*E*		U–U bonding				bridging CO groups	
structure	BP86	M06L	length	WBI	MBO	order	type	ν(CO)
**C8U2CO2-1T**	0.0	0.0	2.550	2.88	2.16	2	2η^2^-μ-CO	1504, 1560
**C8U2CO2-2T**	2.8	4.7	2.537	2.98	2.32	2	2η^2^-μ-CO	1524, 1585
**C8U2CO2-3S**	6.6	10.8	2.501	3.48	2.28	2	2η^2^-μ-CO	1544, 1609
**C8U2CO2-4S**	9.8	12.5	2.515	3.20	2.15	2	2η^2^-μ-CO	1494, 1552

Considering the η^2^-μ-CO^2–^ groups and the C_8_H_8_^2–^ rings each to be dianions leads to the formal f^2^ U­(IV) oxidation state for the uranium atoms. The U=U distances of ∼2.5 Å in all four (C_8_H_8_)_2_U_2_(CO)_2_ structures suggest that the two electrons of each f^2^ U­(IV) join to give a formal double bond consistent with the MBO values around 2. In the triplet structures **C8U2CO2-1T** and **C8U2CO2-2T** this U=U double bond is of the σ + ^2^/_2_π type similar to dioxygen. However, in the singlet structures **C8U2CO2-3S** and **C8U2CO2-4S** the U=U double bond is of the σ + π type similar to ethylene.

### 3.3 The (C_8_H_8_)_2_U_2_(CO)_3_ Tricarbonyl Structures

Seven (C_8_H_8_)_2_U_2_(CO)_3_ tricarbonyl structures were found within 14 kcal/mol of energy of the lowest energy structure **C8U2CO3-1T** (Figure 4 and Table 4). This structure **C8U2CO3-1T** has one bridging η^2^-μ-CO group and two terminal μ-CO groups. Considering the bridging carbonyl group in **C8U2CO3-1T** as the dianion η^2^-μ-CO^2–^ leads to a formal f^3^ U­(III) oxidation state. Interpreting the U=U distance as a formal double bond leaves formally one unpaired electron on each uranium atom in **C8U2CO3-1T** corresponding to the triplet spin state. However, the spin densities on the two uranium atoms in **C8U2CO3-1T** are significantly different with the uranium atom bonded to the two CO oxygen atoms having a spin density of 1.30 whereas the other uranium atom bonded to only a single CO oxygen atom has a spin density of only 0.69.

**Figure 4:**
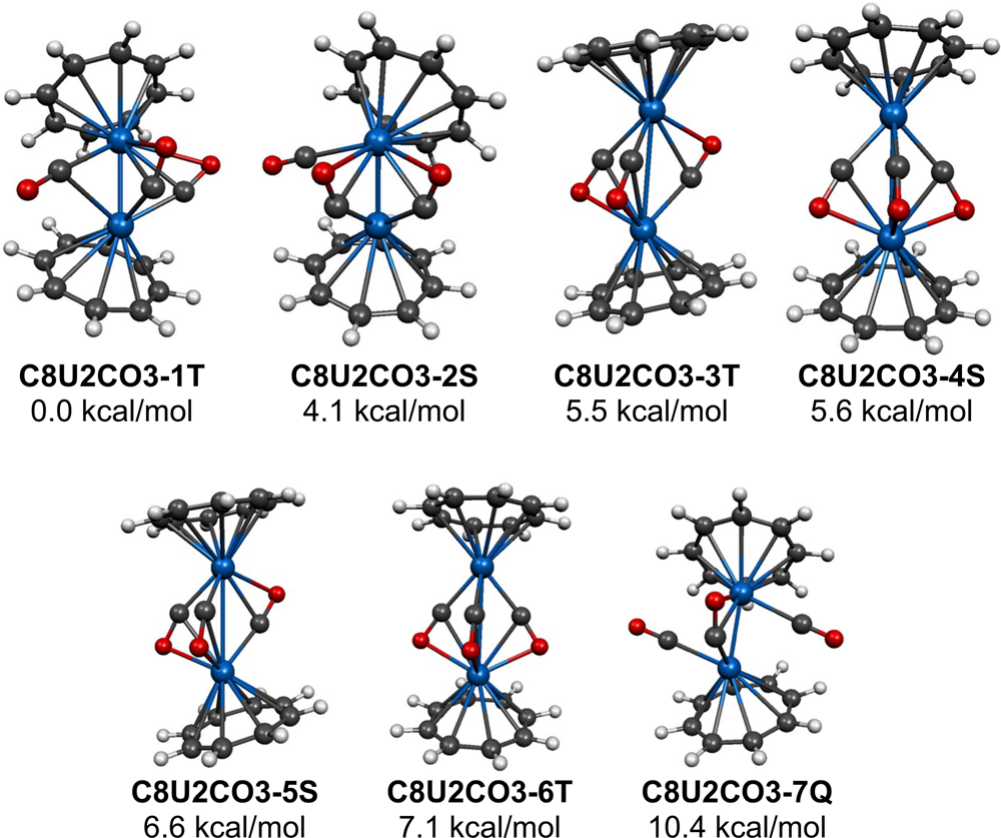
Seven (C_8_H_8_)_2_U_2_(CO)_3_ structures within 14 kcal/mol of energy.

**Table 4:** Seven (C_8_H_8_)_2_U_2_(CO)_3_ Structures within 14 kcal/mol of Energy with Δ*E* in kcal/mol, Bond Lengths in Å, and ν­(CO) Frequencies in cm^–1^

	Δ*E*		U–U bonding				bridging CO groups	
structure	BP86	M06L	length	WBI	MBO	order	type	ν(CO)
**C8U2CO3-1T**	0.0	0.0	2.581	2.96	2.04	2	η^2^-μ-CO	1588
**C8U2CO3-2S**	4.1	4.6	2.501	3.48	2.01	2	2η^2^-μ-CO	1632, 1680
**C8U2CO3-3T**	5.5	8.5	3.604	1.10	1.10	1	3η^2^-μ-CO	1457, 1465, 1532
**C8U2CO3-4S**	5.6	14.5	3.530	1.39	1.12	1	3η^2^-μ-CO	1419, 1424, 1484
**C8U2CO3-5S**	6.6	16.6	3.525	1.43	1.22	1	3η^2^-μ-CO	1382, 1402, 1455
**C8U2CO3-6T**	7.1	4.5	3.809	0.82	0.83	0–1	3η^2^-μ-CO	1440, 1496, 1549
**C8U2CO3-7Q**	10.4	7.4	2.609	2.63	2.05	2	1η^2^-μ-CO	1550

The next lowest energy (C_8_H_8_)_2_U_2_(CO)_3_ structure, namely the singlet structure **C8U2CO3-2S** lying 4.1 kcal/mol in energy above **C8U2CO3-1T**, has two bridging η^2^-μ-CO groups (Figure 4 and Table 4). Considering these bridging carbonyl groups as dianions η^2^-μ-CO^2–^ leads to the f^2^ U­(IV) formal oxidation state for each uranium atom. The central U=U bond of length ∼2.5 Å with an MBO value around 2 in **C8U2CO3-2S** is similar to that in **C8U2CO3-1T** and can likewise be considered a formal double bond using both of the metal electrons in the f^2^ U­(IV) oxidation state. This leaves no unpaired electrons on the uranium atoms accounting for the singlet spin state of **C8U2CO3-2S**.

The four energetically closely spaced (C_8_H_8_)_2_U_2_(CO)_3_ structures **C8U2CO3-3T**, **C8U2CO3-4S**. **C8U2CO3-5S**, and **C8U2CO3-6T**, lying 5.5, 5.6, 6.6, and 7.1 kcal/mol above **C8U2CO3-1T**, all have three bridging η^2^-μ-CO groups and U–U distances in the range 3.5–3.8 Å (Figure 4 and Table 4). Considering these bridging groups as the dianions η^2^-μ-CO^2–^ leads to the formal f^1^ U­(V) oxidation state for the uranium atoms. The MBO and WBI values for these four (C_8_H_8_)_2_U_2_(η^2^-μ-CO)_3_ structures around 1 suggest formal single bonds. These formal single U–U bonds are relatively long as compared with twice the U–U single bond radius of 2 × 1.70 = 3.40 Å^38^ because of the geometry imposed by the three bridging carbonyls in the central U­(η^2^-μ-CO)_3_U units. The single point M06L calculations of the relative energies of the optimized (C_8_H_8_)_2_U_2_(CO)_3_ structures are consistent with those found by the BP86 method except for the significantly higher energies of the two singlet structures **C8U2CO3-4S** and **C8U2CO3-5S** with three bridging η^2^-μ-CO groups.

The next (C_8_H_8_)_2_U_2_(CO)_3_ structure in terms of energy is the quintet structure **C8U2CO3-7Q** lying 10.4 kcal/mol in energy above **C8U2CO3-1T** (Figure 4 and Table 4). Structure **C8U2CO3-7Q** has only a single bridging η^2^-μ-CO group combined with two terminal CO groups. Thus, the formal uranium oxidation state in **C8U2CO3-7Q** appears to be f^3^ U­(III). The U=U distance combined with a MBO value of 2.05 suggests a formal U=U double bond leaving one unpaired electron on each uranium atom. This combined with the U=U double bond being of the σ + ^2^/_2_π type containing two additional unpaired electrons similar to that in triplet dioxygen can account for the four unpaired electrons of the quintet spin state in **C8U2CO3-7Q**.

In the triplet structures **C8U2CO3-3T** and **C8U2CO3-6T** the spin densities are distributed unevenly between the two uranium atoms with the uranium atom forming the most U–O bonds with the carbonyl groups having the highest spin density. Thus, in **C8U2CO3-3T** the uranium atom bonded to two CO oxygen atoms has a spin density of 1.54 whereas the other uranium atom has a spin density of only 0.40. This effect is more extreme in **C8U2CO3-6T** in which one uranium atom is bonded to all three CO oxygen atoms whereas the other uranium atom forms no U–O bonds. Thus, in **C8U2CO3-6T** the two unpaired electrons of the triplet spin state are localized on the uranium atom forming three U–O bonds as indicated by a Mulliken spin density of 2.01. The other uranium atom in **C8U2CO3-6T** not forming any U–O bonds has a negligible spin density of 0.08.

### 3.4 The (C_8_H_8_)_2_U_2_(CO)_5_ Pentacarbonyl Structures

The potential energy surface of the (C_8_H_8_)_2_U_2_(CO)_5_ pentacarbonyl system is more complicated than that of the (C_8_H_8_)_2_U_2_(CO)_*n*_ (*n* = 2, 3, 4) systems with fewer CO groups. Thus, 10 structures lying within 11 kcal/mol of the lowest energy structure **C8U2CO5-1S** were found (Figure 5 and Table 5). All of these (C_8_H_8_)_2_U_2_(CO)_5_ structures have at least one terminal CO group since apparently five CO bridges cannot fit comfortably between two uranium atoms. Structure **C8U2CO5-1S** as well as the higher energy structure **C8U2CO5-4S**, lying 6.3 kcal/mol above **C8U2CO5-1S**, are singlet structures with exclusively terminal CO groups. In **C8U2CO5-1S** three terminal CO groups are bonded to one uranium atom and the remaining two terminal CO groups bonded to the other uranium atom. In **C8U2CO5-4S** the terminal CO groups are more unevenly distributed with four bonded to one uranium atom leaving only one for bonding to the other uranium atom. The U=U distances around ∼2.55 Å in **C8U2CO5-1S** and **C8U2CO5-4S** with MBO values around 2 suggest formal double bonds similar to the U=U double bonds in the (C_8_H_8_)_2_U_2_(CO)_4_ structures **C8U2CO4-4S** and **C8U2CO4-6S** also having exclusively terminal CO groups.

**Figure 5:**
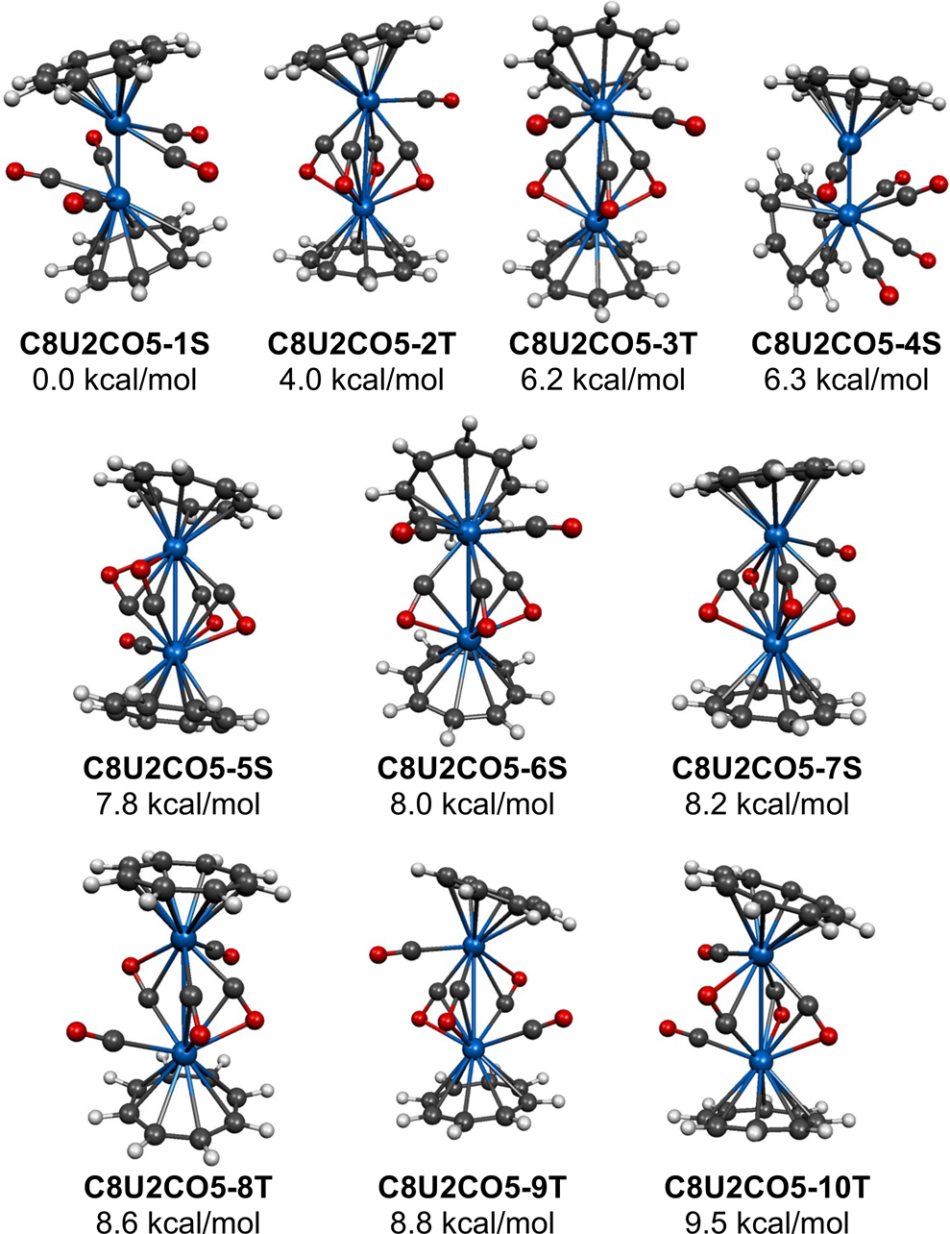
Ten (C_8_H_8_)_2_U_2_(CO)_5_ structures within 11 kcal/mol of energy.

**Table 5:** Ten (C_8_H_8_)_2_U_2_(CO)_5_ Structures within 11 kcal/mol of Energy with Δ*E* in kcal/mol, Bond Lengths in Å, and ν­(CO) Frequencies in cm^–1^

	Δ*E*		U–U bonding				bridging CO groups	
structure	BP86	M06L	length	WBI	MBO	order	type	ν(CO)
**C8U2CO5-1S**	0.0	3.3	2.560	3.49	1.74	2	all terminal CO groups	
**C8U2CO5-2T**	4.0	0.0	3.793	0.83	0.78	0	4η^2^-μ-CO	1565, 1583, 1598, 1637
**C8U2CO5-3T**	6.2	3.9	3.850	0.72	0.69	1	3η^2^-μ-CO	1506, 1539, 1577
**C8U2CO5-4S**	6.3	7.0	2.519	3.63	1.90	2	all terminal CO groups	
**C8U2CO5-5S**	7.8	10.0	3.425	1.64	1.17	1	4η^2^-μ-CO	1535, 1575, 1606, 1649
**C8U2CO5-6S**	8.0	13.8	3.546	1.35	0.98	1	3η^2^-μ-CO	1462, 1466, 1509
**C8U2CO5-7S**	8.2	11.0	3.479	1.48	1.07	1	4η^2^-μ-CO	1470, 1536, 1550, 1675
**C8U2CO5–8T**	8.6	9.7	3.618	1.07	1.00	1	3η^2^-μ-CO	1513, 1533, 1585
**C8U2CO5-9T**	8.8	8.1	3.681	0.98	0.82	1	3η^2^-μ-CO	1559, 1567, 1676
**C8U2CO5-10T**	9.5	10.5	3.672	1.04	0.99	1	3η^2^-μ-CO	1525,1528,1610

The next (C_8_H_8_)_2_U_2_(CO)_5_ structure on the relative energy scale, namely **C8U2CO4-2T**, as well as the higher energy structures **C8U2CO5-5S** and **C8U2CO5-7S**, lying 4.0, 7.8, and 8.2 kcal/mol, respectively, above **C8U2CO5-1S**, have four η^2^-μ-CO bridges and one terminal CO group (Figure 5 and Table 5). The triplet structure **C8U2CO4-2T** has all four η^2^μ-CO groups oriented in the same direction. It can be derived from the quintet (C_8_H_8_)_2_U_2_(CO)_4_ structure **C8U2CO4-5Q** by addition of a terminal CO group to the uranium atom bonded exclusively to carbon atoms of the four bridging η^2^-μ-CO groups. The singlet structure **C8U2CO5-5S** has each uranium atom bonded to two carbon atoms and two oxygen atoms of the set of four bridging η^2^-μ-CO groups. However, in **C8U2CO5-7S** the bonding of the uranium atoms to the set of four bridging η^2^-μ-CO groups is less symmetrically distributed with one uranium atom bonded to three carbon atoms and one oxygen atom and the other uranium atom bonded to three oxygen atoms and one carbon atom. The (C_8_H_8_)_2_U_2_(CO)_5_ structure **C8U2CO5-7S** can be derived from the (C_8_H_8_)_2_U_2_(CO)_4_ structure **C8U2CO4-3S** by adding a terminal CO group to the uranium atom bonded to the carbon atoms of three of the η^2^-μ-CO groups.

### 3.5 CO Dissociation Energies

Table 6 lists the Δ*H* and Δ*G* values for carbonyl dissociation from the (C_8_H_8_)_2_U_2_(CO)_*n*_ (*n* = 5, 4, 3) structures considering the lowest energy structures but also including CO dissociation from the lowest energy structures preserving the spin state, i.e., without intersystem crossing. All of the CO dissociation processes listed in Table 6 are seen to be endothermic implying that none of the lowest energy structures are inherently thermochemically disfavored relative to CO loss. The CO dissociation of the lowest energy structure **C8U2CO5-1S** of the pentacarbonyl (C_8_H_8_)_2_U_2_(CO)_5_ to either the quintet or singlet tetracarbonyl structures is clearly less endothermic than that of the lowest energy structure tetracarbonyl **C8U2CO4-1Q** of the tetracarbonyl (C_8_H_8_)_2_U_2_(CO)_4_ to either the triplet or quintet tricarbonyl structures. This suggests that both the lowest energy pentacarbonyl and tetracarbonyl structures are viable toward CO dissociation. However, the CO dissociation energy of the lowest energy structure **C8U2CO3-1T** of the tricarbonyl (C_8_H_8_)_2_U_2_(CO)_3_ to the lowest energy dicarbonyl **C8U2CO2-1T** is less endothermic than that of the tetracarbonyl **C8U2CO4-1Q**. This suggests that the tricarbonyl (C_8_H_8_)_2_U_2_(CO)_3_ is thermochemically disfavored relative to disproportionation into the dicarbonyl + tetracarbonyl, i.e., into (C_8_H_8_)_2_U_2_(CO)_2_ + (C_8_H_8_)_2_U_2_(CO)_4._ This may relate to the fact that the CO dissociation from the lowest energy tricarbonyl structure **C8U2CO3-1T** involves simple loss of a terminal CO group with no change in the central U_2_(η^2^-μ-CO)_2_ core whereas CO loss from the lowest energy tetracarbonyl structure **C8U2CO4-1Q** requires breakup of the bridging squarate ligand.

**Table 6:** Carbonyl Dissociation Energies from the Lowest Energy (C_8_H_8_)_2_U_2_(CO)_*n*_ (*n* = 5, 4, 3) Structures

reaction	Δ*H*	Δ*G*
**C8U2CO5-1S** → **C8U2CO4-1Q** + CO	12.6	11.9
**C8U2CO5-1S** → **C8U2CO4-3S** + CO	17.5	15.4
**C8U2CO4-1Q** → **C8U2CO3-1T** + CO	24.6	21.7
**C8U2CO4-1Q** → **C8U2CO3-7Q** + CO	35.1	32.1
**C8U2CO3-1T** → **C8U2CO2-1T** + CO	18.5	16.8

## 4 Summary

The most interesting observation from our theoretical study of (C_8_H_8_)_2_U_2_(CO)_*n*_ (*n* = 2, 3, 4, 5) systems is the predicted low energy structures in the tetracarbonyl system of the type (C_8_H_8_)_2_U_2_(η^4^μC_4_O_4_) in which the four CO groups couple to form a bridging C_4_O_4_ squarate unit. Such a tetramerization of carbon monoxide to squarate by organouranium compounds has been observed experimentally by Cloke and co-workers^24^ in sandwich compounds of the type (η^5^-Me_5_C_5_)­U­(η^8^-C_8_H_6_{SiR_3_}_2_) containing both five-membered and eight-membered rings. The bridging squarate (C_8_H_8_)_2_U_2_(η^4^μC_4_O_4_) structures are thermochemically favored to the extent that the lowest energy structure of the tricarbonyl (C_8_H_8_)_2_U_2_(CO)_3_ is thermochemically favored to disproportionate into such a bridging squarate tetracarbonyl structure and the lowest energy structure of the dicarbonyl (C_8_H_8_)_2_U_2_(CO)_2_.

Carbonyl groups in the remaining low-energy (C_8_H_8_)_2_U_2_(CO)_*n*_ (*n* = 2, 3, 4, 5) structures are all isolated, either as terminal CO groups similar to those bonding to d-block metals or as bridging η^2^-μ-CO groups bonded to uranium through both their carbon and oxygen atoms. These bridging carbonyl groups can be regarded formally as dianions η^2^-μ-CO^2–^ derived from the double deprotonation of formaldehyde, HCHO, in determining the formal oxidation state of the central uranium atoms. The preference for η^2^-μ-CO groups forming U–O bonds can be related to the high affinity of uranium for oxygen. The viability of formal uranium oxidation states from +3 to +6, as found experimentally in diverse stable molecules, leads to a variety of spin states and uranium–uranium bonding modes in the low-energy (C_8_H_8_)_2_U_2_(CO)_*n*_ (*n* = 2, 3, 4, 5) structures. This contrasts with the previously studied thorium systems (C_8_H_8_)_2_Th_2_(CO)_*n*_ (*n* = 2, 3, 4, 5)^6^ where the maximum viable formal thorium oxidation state of +4 limits the range of accessible structure types, metal–metal bonding modes, and spin states.

## Supplementary Material





## Notes

Distance matrices and energy rankings for the lowest energy structures (PDF)
Cartesian coordinates of the optimized structures (XYZ)
