# Supplementary material for: Effects of Formal Metal Oxidation State on the Preferred Structure Types in Binuclear Actinide Carbonyl Derivatives: Predicted Tetramerization of Carbon Monoxide to a Bridging Squarate Group in Uranium Chemistry
Source: J Phys Chem A. 2026 Feb 27;130(10):2071–9. doi: 10.1021/acs.jpca.5c07951 (PMC12990112; doi:10.1021/acs.jpca.5c07951)
Supplement: Supplementary file 1 [file jp5c07951_si_001.pdf]

# Effects of Formal Metal Oxidation State on the Preferred Structure Types in Binuclear Actinide Carbonyl Derivatives: Predicted Tetramerization of Carbon Monoxide to a Bridging Squarate Group in Uranium Chemistry

Amr A. A. Attia,[1,†] Alexandru Lupan,[*1,‡] and R. Bruce King[*2,§]

[1]*Faculty of Chemistry and Chemical Engineering, Babeş-Bolyai University, str. Arany János nr. 11, Ro-400028, Cluj-Napoca, Romania*

[2]*Department of Chemistry, University of Georgia, Athens, Georgia, 30602, USA*

## Supporting Information

Complete Gaussian09 Reference.
Table S1: Distance matrices and energy rankings for the lowest energy $(COT)_2U_2(CO)_2$ structures.
Table S2: Distance matrices and energy rankings for the lowest energy $(COT)_2U_2(CO)_3$ structures.
Table S3: Distance matrices and energy rankings for the lowest energy $(COT)_2U_2(CO)_4$ structures.
Table S4: Distance matrices and energy rankings for the lowest energy $(COT)_2U_2(CO)_5$ structures.
Table S5: Mulliken spin densities for the lowest energy $(COT)_2U_2(CO)_n$ ($n$ = 2–5) structures.
Table S6: Single-point M06L/def2-TZVP/ZORA energies for optimized lowest-energy $(COT)_2U_2(CO)_n$ ($n$ = 2–5) structures..

† ORCID: 0000-0002-6604-6795 https://orcid.org/0000-0002-6604-6795
‡ ORCID: 0000-0002-9353-7629 https://orcid.org/0000-0002-9353-7629
§ ORCID: 0000-0001-9177-5220 https://orcid.org/0000-0001-9177-5220

Complete Gaussian09 Reference.

Gaussian 09, Revision E.01, M. J. Frisch, G. W. Trucks, H. B. Schlegel, G. E. Scuseria, M. A. Robb, J. R. Cheeseman, G. Scalmani, V. Barone, B. Mennucci, G. A. Petersson, H. Nakatsuji, M. Caricato, X. Li, H. P. Hratchian, A. F. Izmaylov, J. Bloino, G. Zheng, J. L. Sonnenberg, M. Hada, M. Ehara, K. Toyota, R. Fukuda, J. Hasegawa, M. Ishida, T. Nakajima, Y. Honda, O. Kitao, H. Nakai, T. Vreven, J. A. Montgomery, Jr., J. E. Peralta, F. Ogliaro, M. Bearpark, J. J. Heyd, E. Brothers, K. N. Kudin, V. N. Staroverov, R. Kobayashi, J. Normand, K. Raghavachari, A. Rendell, J. C. Burant, S. S. Iyengar, J. Tomasi, M. Cossi, N. Rega, J. M. Millam, M. Klene, J. E. Knox, J. B. Cross, V. Bakken, C. Adamo, J. Jaramillo, R. Gomperts, R. E. Stratmann, O. Yazyev, A. J. Austin, R. Cammi, C. Pomelli, J. W. Ochterski, R. L. Martin, K. Morokuma, V. G. Zakrzewski, G. A. Voth, P. Salvador, J. J. Dannenberg, S. Dapprich, A. D. Daniels, O. Farkas, J. B. Foresman, J. V. Ortiz, J. Cioslowski, and D. J. Fox, Gaussian, Inc., Wallingford CT, 2016.

Table S1: Distance table for the lowest-lying $U_2COT_2(CO)_2$ optimized structures obtained at the BP86/def2-TZVP/ZORA level of theory. Included are the zero-point corrected absolute energies in (a.u.) and relative energies in (kcal/mol). The hydrogen atoms of the cyclooctatetraene rings are omitted for clarity. S, T, Q and H denote the singlet, triplet, quintet and septet spin states respectively.

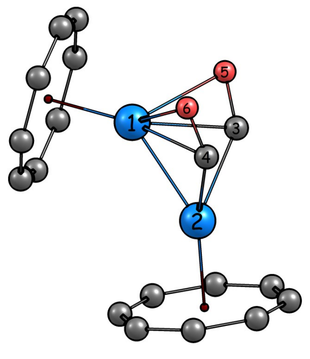


1T. -60343.271243 0.0

| | | 1 | 2 | 3 | 4 | 5 | 6 |
|---|---|---|---|---|---|---|---|
| 1 | U | 0.000000 | | | | | |
| 2 | U | 2.549900 | 0.000000 | | | | |
| 3 | C | 2.359406 | 2.227245 | 0.000000 | | | |
| 4 | C | 2.358979 | 2.229110 | 2.453066 | 0.000000 | | |
| 5 | O | 2.412041 | 3.319100 | 1.241104 | 3.022736 | 0.000000 | |
| 6 | O | 2.410153 | 3.320391 | 3.019718 | 1.241429 | 3.092895 | 0.000000 |

U1-centroid: 1.941062Å U2-centroid: 1.911489Å

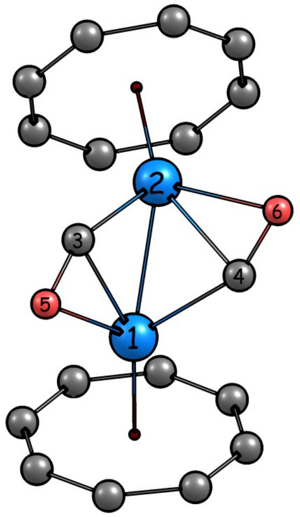


2T. -60343.266766 +2.8

| | | 1 | 2 | 3 | 4 | 5 | 6 |
|---|---|---|---|---|---|---|---|
| 1 | U | 0.000000 | | | | | |
| 2 | U | 2.536881 | 0.000000 | | | | |
| 3 | C | 2.346380 | 2.261629 | 0.000000 | | | |
| 4 | C | 2.260428 | 2.346816 | 2.693272 | 0.000000 | | |
| 5 | O | 2.448048 | 3.360556 | 1.236197 | 3.264216 | 0.000000 | |
| 6 | O | 3.359763 | 2.449771 | 3.264251 | 1.235818 | 4.087923 | 0.000000 |

U1-centroid: 1.926723Å U2-centroid: 1.925763Å

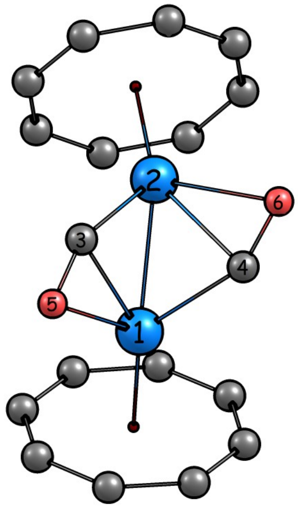


3S. -60343.260682 +6.6

| | | 1 | 2 | 3 | 4 | 5 |
|---|---|---|---|---|---|---|
| 1 | U | 0.000000 | | | | |
| 2 | U | 2.506645 | 0.000000 | | | |
| 3 | C | 2.351755 | 2.260909 | 0.000000 | | |
| 4 | C | 2.260953 | 2.353484 | 2.720106 | 0.000000 | |
| 5 | O | 2.460825 | 3.350604 | 1.231584 | 3.296686 | 0.000000 |
| 6 | O | 3.350579 | 2.464264 | 3.298794 | 1.230922 | 4.126094 |

| | | 6 |
|---|---|---|
| 6 | O | 0.000000 |

U1-centroid: 1.929643Å U2-centroid: 1.928568Å

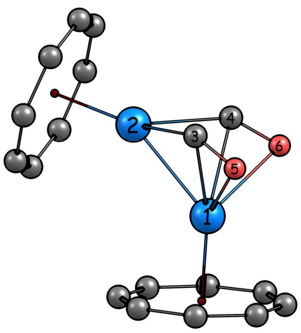


4S. -60343.255621 +9.8

| | | 1 | 2 | 3 | 4 | 5 |
|---|---|---|---|---|---|---|
| 1 | U | 0.000000 | | | | |
| 2 | U | 2.614826 | 0.000000 | | | |
| 3 | C | 2.362672 | 2.233084 | 0.000000 | | |
| 4 | C | 2.362310 | 2.232797 | 2.458822 | 0.000000 | |
| 5 | O | 2.380679 | 3.327971 | 1.243327 | 3.024124 | 0.000000 |
| 6 | O | 2.381300 | 3.328046 | 3.024043 | 1.243186 | 3.090665 |

| | | 6 |
|---|---|---|
| 6 | O | 0.000000 |

U1-centroid: 1.938639Å U2-centroid: 1.917326Å

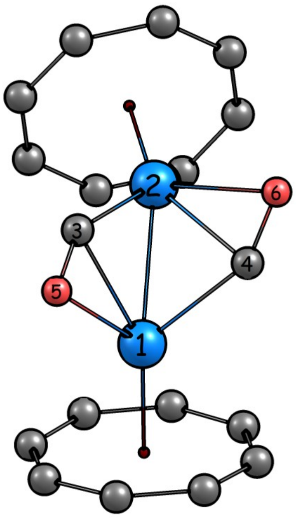


5Q. -60343.248662 +14.2

| | | 1 | 2 | 3 | 4 | 5 |
|---|---|---|---|---|---|---|
| 1 | U | 0.000000 | | | | |
| 2 | U | 2.634851 | 0.000000 | | | |
| 3 | C | 2.411195 | 2.282659 | 0.000000 | | |
| 4 | C | 2.281639 | 2.411257 | 2.795614 | 0.000000 | |
| 5 | O | 2.456993 | 3.370798 | 1.231049 | 3.258011 | 0.000000 |
| 6 | O | 3.369798 | 2.456551 | 3.258731 | 1.231102 | 3.983695 |

| | | 6 |
|---|---|---|
| 6 | O | 0.000000 |

U1-centroid: 1.922477Å U2-centroid: 1.921781Å

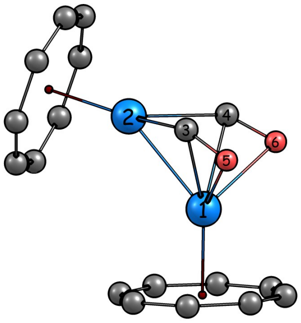

6Q. -60343.244744 +16.6

| | | 1 | 2 | 3 | 4 | 5 |
|---|---|---|---|---|---|---|
| 1 | U | 0.000000 | | | | |
| 2 | U | 2.646828 | 0.000000 | | | |
| 3 | C | 2.397975 | 2.296074 | 0.000000 | | |
| 4 | C | 2.397958 | 2.299074 | 2.829810 | 0.000000 | |
| 5 | O | 2.434864 | 3.384048 | 1.233890 | 3.347152 | 0.000000 |
| 6 | O | 2.436999 | 3.386581 | 3.343864 | 1.233303 | 3.417432 |

| | | 6 |
|---|---|---|
| 6 | O | 0.000000 |

U1-centroid: 1.926662Å U2-centroid: 1.916216Å

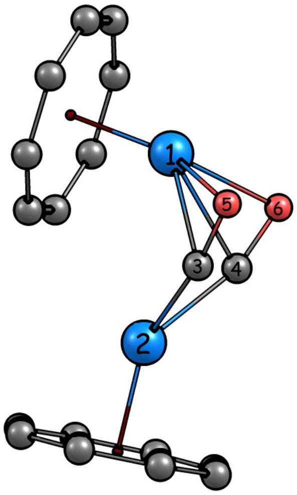

7T. -60343.240917 +19.0

| | | 1 | 2 | 3 | 4 | 5 |
|---|---|---|---|---|---|---|
| 1 | U | 0.000000 | | | | |
| 2 | U | 3.297792 | 0.000000 | | | |
| 3 | C | 2.394557 | 2.154993 | 0.000000 | | |
| 4 | C | 2.397709 | 2.154617 | 2.174198 | 0.000000 | |
| 5 | O | 2.261797 | 3.394205 | 1.284335 | 2.816583 | 0.000000 |
| 6 | O | 2.265015 | 3.393062 | 2.820093 | 1.283401 | 2.895196 |

| | | 6 |
|---|---|---|
| 6 | O | 0.000000 |

U1-centroid: 1.940051Å U2-centroid: 1.945038Å

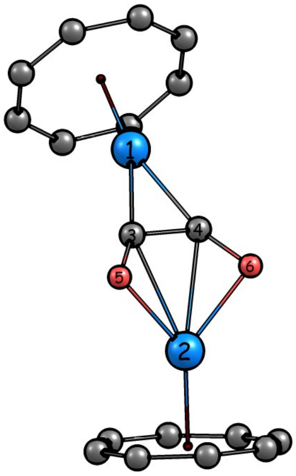

8Q. -60343.238836 +20.3

| | | 1 | 2 | 3 | 4 | 5 |
|---|---|---|---|---|---|---|
| 1 | U | 0.000000 | | | | |
| 2 | U | 4.512071 | 0.000000 | | | |
| 3 | C | 2.313521 | 2.554459 | 0.000000 | | |
| 4 | C | 2.313709 | 2.552378 | 1.454892 | 0.000000 | |
| 5 | O | 3.642502 | 2.160286 | 1.354941 | 2.450163 | 0.000000 |
| 6 | O | 3.643230 | 2.159283 | 2.450401 | 1.355789 | 2.864047 |

| | | 6 |
|---|---|---|
| 6 | O | 0.000000 |

U1-centroid: 1.919429Å U2-centroid: 1.886611Å

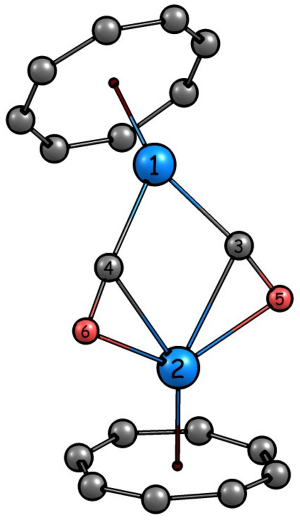

9S. -60343.236987 +21.5

| | | 1 | 2 | 3 | 4 | 5 |
|---|---|---|---|---|---|---|
| 1 | U | 0.000000 | | | | |
| 2 | U | 3.777833 | 0.000000 | | | |
| 3 | C | 2.204141 | 2.508130 | 0.000000 | | |
| 4 | C | 2.204267 | 2.509678 | 2.720283 | 0.000000 | |
| 5 | O | 3.459419 | 2.292940 | 1.263978 | 3.540835 | 0.000000 |
| 6 | O | 3.457402 | 2.292446 | 3.545513 | 1.263802 | 4.028006 |

| | | 6 |
|---|---|---|
| 6 | O | 0.000000 |

U1-centroid: 1.919429Å U2-centroid: 1.922155Å

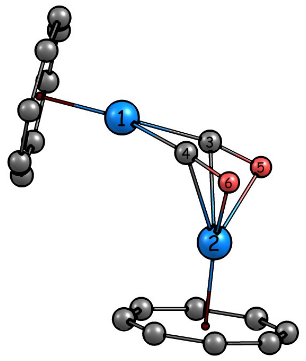

10T. -60343.233241 +23.8

| | | 1 | 2 | 3 | 4 | 5 |
|---|---|---|---|---|---|---|
| 1 | U | 0.000000 | | | | |
| 2 | U | 3.500400 | 0.000000 | | | |
| 3 | C | 2.154549 | 2.423435 | 0.000000 | | |
| 4 | C | 2.150518 | 2.416228 | 2.115097 | 0.000000 | |
| 5 | O | 3.416222 | 2.228553 | 1.291412 | 2.770954 | 0.000000 |
| 6 | O | 3.414528 | 2.224611 | 2.779506 | 1.291583 | 2.852805 |

| | | 6 |
|---|---|---|
| 6 | O | 0.000000 |

U1-centroid: 1.928681Å U2-centroid: 1.922864Å

Table S2: Distance table for the lowest-lying $U_2COT_2(CO)_3$ optimized structures obtained at the BP86/def2-TZVP/ZORA level of theory. Included are the zero-point corrected absolute energies in (a.u.) and relative energies in (kcal/mol). The hydrogen atoms of the cyclooctatetraene rings are omitted for clarity. S, T, Q and H denote the singlet, triplet, quintet and septet spin states respectively.

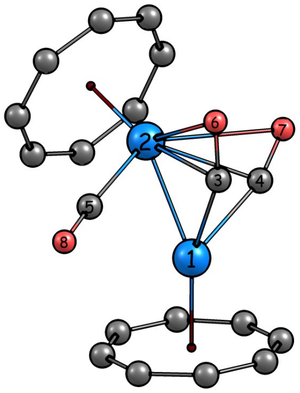


1T. -60456.627452  0.0

| | | 1 | 2 | 3 | 4 | 5 |
|---|---|---|---|---|---|---|
| 1 | U | 0.000000 | | | | |
| 2 | U | 2.580767 | 0.000000 | | | |
| 3 | C | 2.263517 | 2.405688 | 0.000000 | | |
| 4 | C | 2.252871 | 2.539965 | 2.437572 | 0.000000 | |
| 5 | C | 2.681022 | 2.332900 | 2.696323 | 4.100685 | 0.000000 |
| 6 | O | 3.366663 | 2.524576 | 1.227017 | 3.016817 | 3.141891 |
| 7 | O | 3.412244 | 2.953967 | 3.093694 | 1.195012 | 4.893909 |
| 8 | O | 3.355177 | 3.508852 | 3.445499 | 5.081291 | 1.177532 |
| | | 6 | 7 | 8 | | |
| 6 | O | 0.000000 | | | | |
| 7 | O | 3.241983 | 0.000000 | | | |
| 8 | O | 3.948777 | 5.953292 | 0.000000 | | |

U1-centroid: 1.940878Å  U2-centroid: 1.930393Å

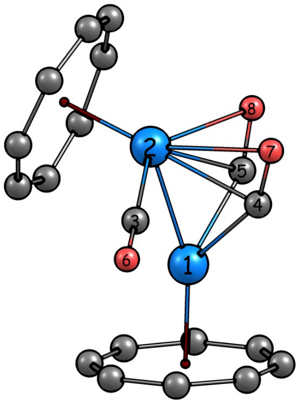


2S. -60456.620884  +4.1

| | | 1 | 2 | 3 | 4 | 5 |
|---|---|---|---|---|---|---|
| 1 | U | 0.000000 | | | | |
| 2 | U | 2.501449 | 0.000000 | | | |
| 3 | C | 2.951793 | 2.356503 | 0.000000 | | |
| 4 | C | 2.239385 | 2.418501 | 2.693259 | 0.000000 | |
| 5 | C | 2.263367 | 2.470789 | 4.192716 | 2.369857 | 0.000000 |
| 6 | O | 3.728944 | 3.529207 | 1.173352 | 3.482463 | 5.231290 |
| 7 | O | 3.344905 | 2.607300 | 3.003473 | 1.216266 | 2.938062 |
| 8 | O | 3.364980 | 2.676249 | 4.708190 | 2.925464 | 1.213380 |
| | | 6 | 7 | 8 | | |
| 6 | O | 0.000000 | | | | |
| 7 | O | 3.796605 | 0.000000 | | | |
| 8 | O | 5.813614 | 3.004244 | 0.000000 | | |

U1-centroid: 1.957461Å  U2-centroid: 1.924721Å

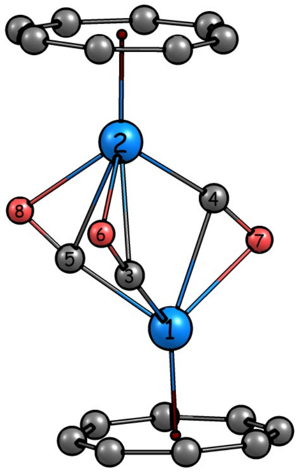

3T. -60456.618704 +5.5

| | | 1 | 2 | 3 | 4 | 5 |
|---|---|---|---|---|---|---|
| 1 | U | 0.000000 | | | | |
| 2 | U | 3.604001 | 0.000000 | | | |
| 3 | C | 2.190098 | 2.513409 | 0.000000 | | |
| 4 | C | 2.455491 | 2.241910 | 2.789386 | 0.000000 | |
| 5 | C | 2.190289 | 2.514736 | 2.175116 | 2.789984 | 0.000000 |
| 6 | O | 3.420415 | 2.355299 | 1.250671 | 3.482248 | 2.831939 |
| 7 | O | 2.341328 | 3.478490 | 3.461694 | 1.248741 | 3.461268 |
| 8 | O | 3.420259 | 2.356326 | 2.831591 | 3.482042 | 1.250441 |

| | | 6 | 7 | 8 |
|---|---|---|---|---|
| 6 | O | 0.000000 | | |
| 7 | O | 4.390489 | 0.000000 | |
| 8 | O | 2.967657 | 4.389284 | 0.000000 |

U1-centroid: 1.938249Å U2-centroid: 1.942758Å

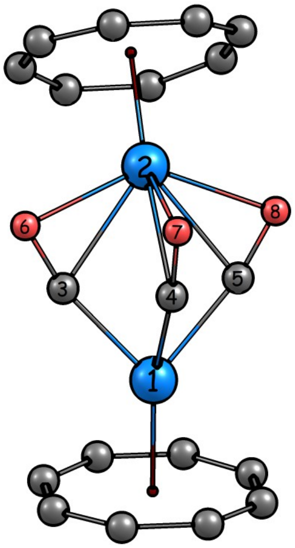

4S. -60456.618479 +5.6

| | | 1 | 2 | 3 | 4 | 5 |
|---|---|---|---|---|---|---|
| 1 | U | 0.000000 | | | | |
| 2 | U | 3.530324 | 0.000000 | | | |
| 3 | C | 2.180051 | 2.431003 | 0.000000 | | |
| 4 | C | 2.182291 | 2.554136 | 2.122611 | 0.000000 | |
| 5 | C | 2.179025 | 2.430469 | 2.955958 | 2.123842 | 0.000000 |
| 6 | O | 3.422914 | 2.287194 | 1.266238 | 2.766734 | 3.754407 |
| 7 | O | 3.398006 | 2.327277 | 2.767071 | 1.258032 | 2.767386 |
| 8 | O | 3.422044 | 2.287878 | 3.755351 | 2.768716 | 1.266085 |

| | | 6 | 7 | 8 |
|---|---|---|---|---|
| 6 | O | 0.000000 | | |
| 7 | O | 2.862170 | 0.000000 | |
| 8 | O | 4.227377 | 2.863589 | 0.000000 |

U1-centroid: 1.961028Å U2-centroid: 1.943837Å

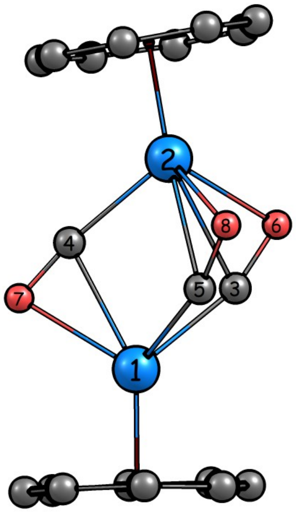


5S. -60456.616987 +6.6

| | | 1 | 2 | 3 | 4 | 5 |
|---|---|---|---|---|---|---|
| 1 | U | 0.000000 | | | | |
| 2 | U | 3.524919 | 0.000000 | | | |
| 3 | C | 2.172144 | 2.501079 | 0.000000 | | |
| 4 | C | 2.383685 | 2.206580 | 2.846465 | 0.000000 | |
| 5 | C | 2.173641 | 2.506613 | 1.950244 | 2.835299 | 0.000000 |
| 6 | O | 3.405731 | 2.272015 | 1.271844 | 3.459509 | 2.649586 |
| 7 | O | 2.328521 | 3.462139 | 3.572930 | 1.262772 | 3.556159 |
| 8 | O | 3.404415 | 2.274792 | 2.648235 | 3.440180 | 1.270378 |
| | | 6 | 7 | 8 | | |
| 6 | O | 0.000000 | | | | |
| 7 | O | 4.426589 | 0.000000 | | | |
| 8 | O | 2.769408 | 4.401622 | 0.000000 | | |

U1-centroid: 1.950279Å U2-centroid: 1.949997Å

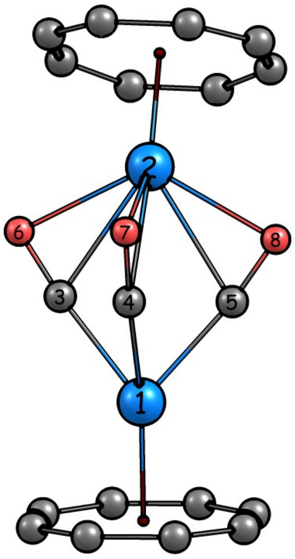


6T. -60456.616118 +7.1

| | | 1 | 2 | 3 | 4 | 5 |
|---|---|---|---|---|---|---|
| 1 | U | 0.000000 | | | | |
| 2 | U | 3.808919 | 0.000000 | | | |
| 3 | C | 2.191304 | 2.568588 | 0.000000 | | |
| 4 | C | 2.171002 | 2.677825 | 2.147226 | 0.000000 | |
| 5 | C | 2.189448 | 2.563235 | 2.823339 | 2.144416 | 0.000000 |
| 6 | O | 3.420714 | 2.381171 | 1.242797 | 2.816403 | 3.661919 |
| 7 | O | 3.399571 | 2.354672 | 2.809350 | 1.257277 | 2.806172 |
| 8 | O | 3.419960 | 2.379127 | 3.666506 | 2.816560 | 1.243305 |
| | | 6 | 7 | 8 | | |
| 6 | O | 0.000000 | | | | |
| 7 | O | 2.966546 | 0.000000 | | | |
| 8 | O | 4.208240 | 2.966555 | 0.000000 | | |

U1-centroid: 1.945050Å U2-centroid: 1.926725Å

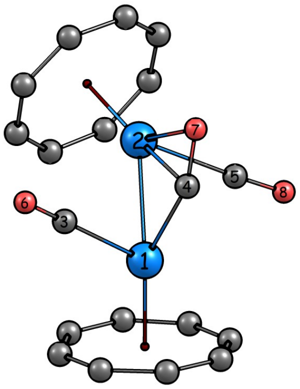


7Q. -60456.610950 +10.4

| | | 1 | 2 | 3 | 4 | 5 |
|---|---|---|---|---|---|---|
| 1 | U | 0.000000 | | | | |
| 2 | U | 2.608753 | 0.000000 | | | |
| 3 | C | 2.341622 | 3.029742 | 0.000000 | | |
| 4 | C | 2.262423 | 2.382072 | 2.696766 | 0.000000 | |
| 5 | C | 2.990037 | 2.355821 | 4.690155 | 3.004799 | 0.000000 |
| 6 | O | 3.513210 | 3.817145 | 1.171906 | 3.520761 | 5.718160 |
| 7 | O | 3.356695 | 2.434706 | 3.394809 | 1.235303 | 3.275311 |
| 8 | O | 3.791127 | 3.527720 | 5.719974 | 3.855660 | 1.172691 |
| | | 6 | 7 | 8 | | |
| 6 | O | 0.000000 | | | | |
| 7 | O | 3.963352 | 0.000000 | | | |
| 8 | O | 6.785687 | 4.142175 | 0.000000 | | |

U1-centroid: 1.925510Å U2-centroid: 1.939062Å

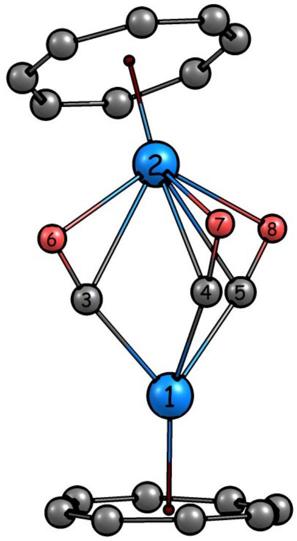


8Q. -60456.604213 +14.6

| | | 1 | 2 | 3 | 4 | 5 |
|---|---|---|---|---|---|---|
| 1 | U | 0.000000 | | | | |
| 2 | U | 3.909509 | 0.000000 | | | |
| 3 | C | 2.261656 | 2.685414 | 0.000000 | | |
| 4 | C | 2.197367 | 2.644088 | 2.075862 | 0.000000 | |
| 5 | C | 2.259892 | 2.677590 | 3.005958 | 2.065875 | 0.000000 |
| 6 | O | 3.477431 | 2.456593 | 1.229649 | 2.750189 | 3.798171 |
| 7 | O | 3.445079 | 2.307855 | 2.756491 | 1.264920 | 2.748227 |
| 8 | O | 3.477697 | 2.447671 | 3.801468 | 2.740756 | 1.231321 |
| | | 6 | 7 | 8 | | |
| 6 | O | 0.000000 | | | | |
| 7 | O | 2.904001 | 0.000000 | | | |
| 8 | O | 4.299640 | 2.893672 | 0.000000 | | |

U1-centroid: 1.931451Å U2-centroid: 1.912495Å

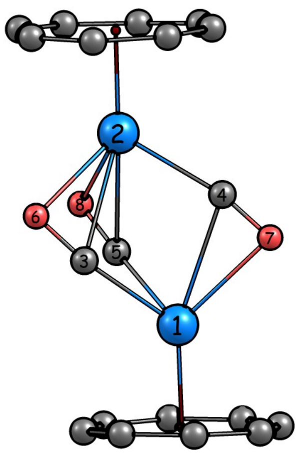


9Q. -60456.602352 +15.8

| | | 1 | 2 | 3 | 4 | 5 |
|---|---|---|---|---|---|---|
| 1 | U | 0.000000 | | | | |
| 2 | U | 3.766099 | 0.000000 | | | |
| 3 | C | 2.228524 | 2.571268 | 0.000000 | | |
| 4 | C | 2.616798 | 2.311739 | 2.883858 | 0.000000 | |
| 5 | C | 2.228006 | 2.569223 | 2.080024 | 2.848360 | 0.000000 |
| 6 | O | 3.462285 | 2.370397 | 1.248858 | 3.550166 | 2.740445 |
| 7 | O | 2.460536 | 3.518547 | 3.515754 | 1.222871 | 3.481570 |
| 8 | O | 3.461975 | 2.373985 | 2.742479 | 3.515094 | 1.248293 |

| | | 6 | 7 | 8 |
|---|---|---|---|---|
| 6 | O | 0.000000 | | |
| 7 | O | 4.420624 | 0.000000 | |
| 8 | O | 2.863760 | 4.386620 | 0.000000 |

U1-centroid: 1.932517Å U2-centroid: 1.935165Å

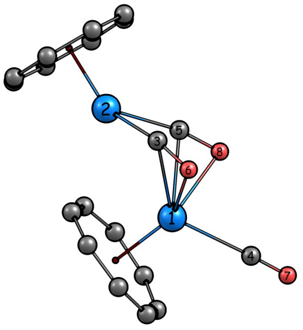


10T. -60456.594598 +20.6

| | | 1 | 2 | 3 | 4 | 5 |
|---|---|---|---|---|---|---|
| 1 | U | 0.000000 | | | | |
| 2 | U | 3.452857 | 0.000000 | | | |
| 3 | C | 2.480820 | 2.145796 | 0.000000 | | |
| 4 | C | 2.450688 | 5.624983 | 4.439155 | 0.000000 | |
| 5 | C | 2.476430 | 2.155338 | 2.095011 | 3.928872 | 0.000000 |
| 6 | O | 2.298826 | 3.383321 | 1.272595 | 3.773810 | 2.764743 |
| 7 | O | 3.611723 | 6.731155 | 5.522009 | 1.161634 | 4.919057 |
| 8 | O | 2.373477 | 3.391113 | 2.771566 | 2.965795 | 1.258817 |

| | | 6 | 7 | 8 |
|---|---|---|---|---|
| 6 | O | 0.000000 | | |
| 7 | O | 4.780031 | 0.000000 | |
| 8 | O | 2.894316 | 3.827302 | 0.000000 |

U1-centroid: 1.975845Å U2-centroid: 1.931241Å

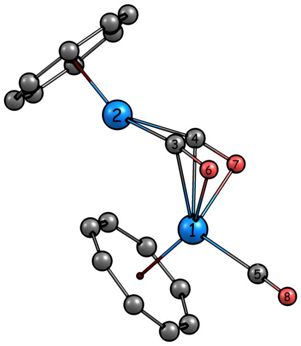


11Q. -60456.587555 +25.0

| | | 1 | 2 | 3 | 4 | 5 |
|---|---|---|---|---|---|---|
| 1 | U | 0.000000 | | | | |
| 2 | U | 3.605596 | 0.000000 | | | |
| 3 | C | 2.512368 | 2.187505 | 0.000000 | | |
| 4 | C | 2.524092 | 2.184108 | 1.953494 | 0.000000 | |
| 5 | C | 2.456623 | 5.793120 | 4.029920 | 4.467800 | 0.000000 |
| 6 | O | 2.330311 | 3.434882 | 1.268111 | 2.656050 | 3.024678 |
| 7 | O | 2.284693 | 3.428238 | 2.654212 | 1.275299 | 3.759923 |
| 8 | O | 3.616610 | 6.901255 | 5.031476 | 5.546981 | 1.160721 |
| | | 6 | 7 | 8 | | |
| 6 | O | 0.000000 | | | | |
| 7 | O | 2.781902 | 0.000000 | | | |
| 8 | O | 3.909653 | 4.767136 | 0.000000 | | |

U1-centroid: 1.925202Å U2-centroid: 1.959881Å

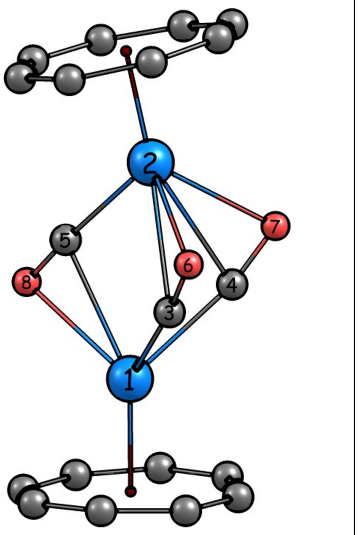


12H. -60456.586402 +25.8

| | | 1 | 2 | 3 | 4 | 5 |
|---|---|---|---|---|---|---|
| 1 | U | 0.000000 | | | | |
| 2 | U | 3.794126 | 0.000000 | | | |
| 3 | C | 2.313165 | 2.643381 | 0.000000 | | |
| 4 | C | 2.324324 | 2.646451 | 2.141700 | 0.000000 | |
| 5 | C | 2.566484 | 2.341330 | 3.011515 | 2.897225 | 0.000000 |
| 6 | O | 3.522833 | 2.479360 | 1.225908 | 2.806521 | 3.679391 |
| 7 | O | 3.532382 | 2.477866 | 2.801626 | 1.226167 | 3.546894 |
| 8 | O | 2.559897 | 3.553464 | 3.785471 | 3.598239 | 1.215204 |
| | | 6 | 7 | 8 | | |
| 6 | O | 0.000000 | | | | |
| 7 | O | 2.969449 | 0.000000 | | | |
| 8 | O | 4.652136 | 4.439523 | 0.000000 | | |

U1-centroid: 1.920145Å U2-centroid: 1.921644Å

Table S3: Distance table for the lowest-lying $U_2COT_2(CO)_4$ optimized structures obtained at the BP86/def2-TZVP/ZORA level of theory. Included are the zero-point corrected absolute energies in (a.u.) and relative energies in (kcal/mol). The hydrogen atoms of the cyclooctatetraene rings are omitted for clarity. S, T, Q and H denote the singlet, triplet, quintet and septet spin states respectively.

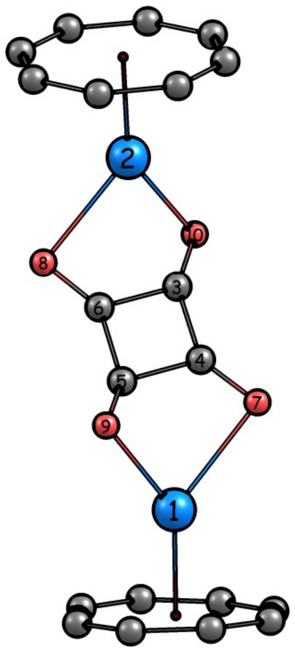


1Q. -60569.991425 0.0

| | | 1 | 2 | 3 | 4 | 5 |
|---|---|---|---|---|---|---|
| 1 | U | 0.000000 | | | | |
| 2 | U | 6.358413 | 0.000000 | | | |
| 3 | C | 4.093187 | 2.469151 | 0.000000 | | |
| 4 | C | 2.741426 | 3.831627 | 1.480688 | 0.000000 | |
| 5 | C | 2.468132 | 4.026968 | 2.138388 | 1.480460 | 0.000000 |
| 6 | C | 3.895946 | 2.711940 | 1.468537 | 2.039991 | 1.481330 |
| 7 | O | 2.423241 | 4.945161 | 2.618897 | 1.274786 | 2.432379 |
| 8 | O | 5.032886 | 2.398660 | 2.423750 | 3.302320 | 2.615845 |
| 9 | O | 2.151817 | 5.077243 | 3.404308 | 2.478793 | 1.374761 |
| 10 | O | 5.287668 | 2.170704 | 1.363987 | 2.703266 | 3.441969 |

| | | 6 | 7 | 8 | 9 | 10 |
|---|---|---|---|---|---|---|
| 6 | C | 0.000000 | | | | |
| 7 | O | 3.303359 | 0.000000 | | | |
| 8 | O | 1.277536 | 4.571911 | 0.000000 | | |
| 9 | O | 2.670413 | 2.963976 | 3.567207 | 0.000000 | |
| 10 | O | 2.486398 | 3.628050 | 2.968126 | 4.750895 | 0.000000 |

U1-centroid: 1.900550Å U2-centroid: 1.897292Å

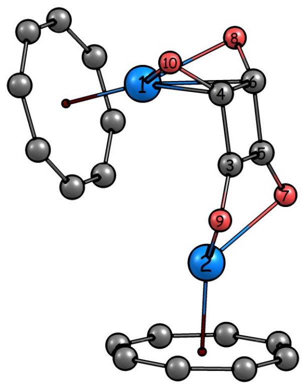


2Q. -60569.989492 +1.2

| | | 1 | 2 | 3 | 4 | 5 |
|---|---|---|---|---|---|---|
| 1 | U | 0.000000 | | | | |
| 2 | U | 4.020813 | 0.000000 | | | |
| 3 | C | 2.923443 | 2.610687 | 0.000000 | | |
| 4 | C | 2.381631 | 3.986988 | 1.535592 | 0.000000 | |
| 5 | C | 2.920366 | 2.617782 | 1.425863 | 2.107287 | 0.000000 |
| 6 | C | 2.380043 | 3.993572 | 2.108788 | 1.461708 | 1.536625 |
| 7 | O | 3.877141 | 2.269165 | 2.451512 | 3.418491 | 1.316476 |
| 8 | O | 2.224812 | 4.818009 | 3.304031 | 2.463470 | 2.591841 |
| 9 | O | 3.891546 | 2.261371 | 1.318319 | 2.703854 | 2.453868 |
| 10 | O | 2.230206 | 4.806285 | 2.588921 | 1.327755 | 3.301331 |

| | | 6 | 7 | 8 | 9 | 10 |
|---|---|---|---|---|---|---|
| 6 | C | 0.000000 | | | | |
| 7 | O | 2.703448 | 0.000000 | | | |
| 8 | O | 1.329336 | 3.503606 | 0.000000 | | |
| 9 | O | 3.421813 | 3.001831 | 4.598424 | 0.000000 | |
| 10 | O | 2.463269 | 4.591475 | 2.943924 | 3.503973 | 0.000000 |

U1-centroid: 1.934847Å U2-centroid: 1.925180Å

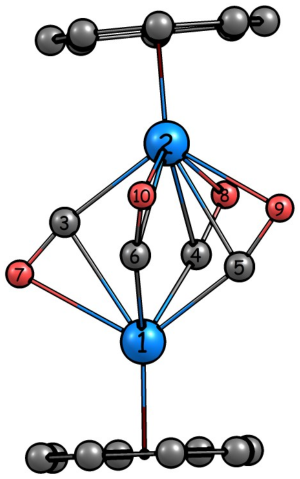


3S. -60569.985852 +3.5

| | | 1 | 2 | 3 | 4 | 5 |
|---|---|---|---|---|---|---|
| 1 | U | 0.000000 | | | | |
| 2 | U | 3.459738 | 0.000000 | | | |
| 3 | C | 2.511246 | 2.314357 | 0.000000 | | |
| 4 | C | 2.249155 | 2.523164 | 2.525336 | 0.000000 | |
| 5 | C | 2.191500 | 2.515027 | 3.287211 | 2.118516 | 0.000000 |
| 6 | C | 2.249421 | 2.521200 | 2.521817 | 3.244248 | 2.118353 |
| 7 | O | 2.540993 | 3.516331 | 1.216815 | 3.148342 | 4.023859 |
| 8 | O | 3.447847 | 2.410970 | 3.142117 | 1.236014 | 2.735214 |
| 9 | O | 3.409351 | 2.353619 | 3.944245 | 2.765080 | 1.255349 |
| 10 | O | 3.448590 | 2.406795 | 3.137213 | 3.987125 | 2.735668 |

| | | 6 | 7 | 8 | 9 | 10 |
|---|---|---|---|---|---|---|
| 6 | C | 0.000000 | | | | |
| 7 | O | 3.142669 | 0.000000 | | | |
| 8 | O | 3.987446 | 3.974796 | 0.000000 | | |
| 9 | O | 2.764761 | 4.896695 | 2.838771 | 0.000000 | |
| 10 | O | 1.236737 | 3.967958 | 4.429324 | 2.838679 | 0.000000 |

U1-centroid: 1.964550Å U2-centroid: 1.948573Å

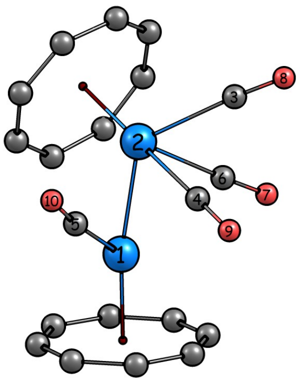


4S. -60569.981244 +6.4

| | | 1 | 2 | 3 | 4 | 5 |
|---|---|---|---|---|---|---|
| 1 | U | 0.000000 | | | | |
| 2 | U | 2.479994 | 0.000000 | | | |
| 3 | C | 4.287537 | 2.354296 | 0.000000 | | |
| 4 | C | 2.771491 | 2.296396 | 2.479761 | 0.000000 | |
| 5 | C | 2.288559 | 2.703827 | 4.462211 | 2.671934 | 0.000000 |
| 6 | C | 2.825104 | 2.277593 | 2.480370 | 2.707297 | 4.263747 |
| 7 | O | 3.636772 | 3.458591 | 3.235460 | 3.556237 | 5.305496 |
| 8 | O | 5.310711 | 3.519797 | 1.167850 | 3.243568 | 5.499244 |
| 9 | O | 3.503744 | 3.474767 | 3.257979 | 1.180788 | 3.356637 |
| 10 | O | 3.464908 | 3.355220 | 4.881723 | 3.286160 | 1.184698 |

| | | 6 | 7 | 8 | 9 | 10 |
|---|---|---|---|---|---|---|
| 6 | C | 0.000000 | | | | |
| 7 | O | 1.181754 | 0.000000 | | | |
| 8 | O | 3.221862 | 3.647078 | 0.000000 | | |
| 9 | O | 3.488571 | 4.069157 | 3.705424 | 0.000000 | |
| 10 | O | 5.186534 | 6.272613 | 5.859584 | 3.872497 | 0.000000 |

U1-centroid: 1.954346Å U2-centroid: 1.939075Å

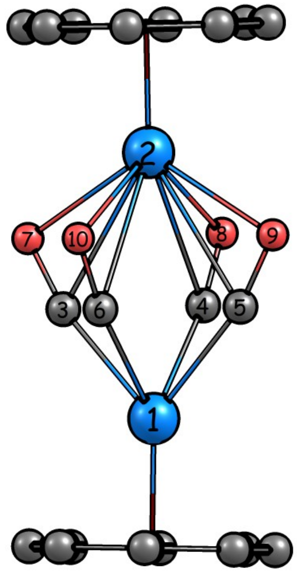


5Q. -60569.979823 +7.3

| | | 1 | 2 | 3 | 4 | 5 |
|---|---|---|---|---|---|---|
| 1 | U | 0.000000 | | | | |
| 2 | U | 4.004025 | 0.000000 | | | |
| 3 | C | 2.266967 | 2.801652 | 0.000000 | | |
| 4 | C | 2.261824 | 2.783733 | 2.176679 | 0.000000 | |
| 5 | C | 2.269905 | 2.804161 | 3.084423 | 2.152514 | 0.000000 |
| 6 | C | 2.258172 | 2.778733 | 2.151029 | 3.036901 | 2.176854 |
| 7 | O | 3.467171 | 2.480929 | 1.223480 | 2.840389 | 3.828823 |
| 8 | O | 3.466466 | 2.454172 | 2.845719 | 1.227260 | 2.810471 |
| 9 | O | 3.469085 | 2.478524 | 3.824544 | 2.810592 | 1.223742 |
| 10 | O | 3.464255 | 2.455000 | 2.812522 | 3.787052 | 2.850140 |

| | | 6 | 7 | 8 | 9 | 10 |
|---|---|---|---|---|---|---|
| 6 | C | 0.000000 | | | | |
| 7 | O | 2.809715 | 0.000000 | | | |
| 8 | O | 3.781081 | 3.023610 | 0.000000 | | |
| 9 | O | 2.835580 | 4.262156 | 2.972015 | 0.000000 | |
| 10 | O | 1.227051 | 2.975867 | 4.219229 | 3.022861 | 0.000000 |

U1-centroid: 1.915482Å U2-centroid: 1.952014Å

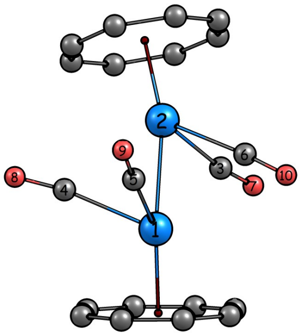


6S. -60569.978094 +8.4

| | | 1 | 2 | 3 | 4 | 5 |
|---|---|---|---|---|---|---|
| 1 | U | 0.000000 | | | | |
| 2 | U | 2.536040 | 0.000000 | | | |
| 3 | C | 2.774946 | 2.291816 | 0.000000 | | |
| 4 | C | 2.293226 | 2.780189 | 4.047803 | 0.000000 | |
| 5 | C | 2.285966 | 2.739888 | 4.285976 | 2.575206 | 0.000000 |
| 6 | C | 2.753419 | 2.288096 | 2.576188 | 4.299806 | 2.922210 |
| 7 | O | 3.640272 | 3.461739 | 1.178930 | 5.066909 | 5.359308 |
| 8 | O | 3.462045 | 3.651462 | 5.071305 | 1.178739 | 3.393876 |
| 9 | O | 3.464710 | 3.419966 | 5.245197 | 3.364067 | 1.181290 |
| 10 | O | 3.445809 | 3.466889 | 3.368646 | 5.266548 | 3.594563 |

| | | 6 | 7 | 8 | 9 | 10 |
|---|---|---|---|---|---|---|
| 6 | C | 0.000000 | | | | |
| 7 | O | 3.393875 | 0.000000 | | | |
| 8 | O | 5.374881 | 6.079564 | 0.000000 | | |
| 9 | O | 3.584074 | 6.349105 | 3.903104 | 0.000000 | |
| 10 | O | 1.180668 | 3.906261 | 6.370434 | 4.168654 | 0.000000 |

U1-centroid: 1.951091Å U2-centroid: 1.951624Å

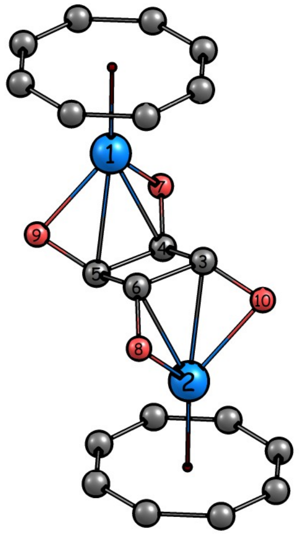


7S. -60569.977291 +8.9

| | | 1 | 2 | 3 | 4 | 5 |
|---|---|---|---|---|---|---|
| 1 | U | 0.000000 | | | | |
| 2 | U | 4.598239 | 0.000000 | | | |
| 3 | C | 2.699659 | 2.360765 | 0.000000 | | |
| 4 | C | 2.369978 | 2.690482 | 1.519414 | 0.000000 | |
| 5 | C | 2.360854 | 2.698371 | 2.138319 | 1.503857 | 0.000000 |
| 6 | C | 2.690644 | 2.370093 | 1.503695 | 2.137426 | 1.519769 |
| 7 | O | 2.191436 | 4.009268 | 2.558900 | 1.355311 | 2.458654 |
| 8 | O | 4.009672 | 2.191358 | 2.458538 | 3.279237 | 2.558786 |
| 9 | O | 2.183193 | 4.019681 | 3.286655 | 2.465999 | 1.357839 |
| 10 | O | 4.020951 | 2.183656 | 1.357679 | 2.561288 | 3.286344 |

| | | 6 | 7 | 8 | 9 | 10 |
|---|---|---|---|---|---|---|
| 6 | C | 0.000000 | | | | |
| 7 | O | 3.279216 | 0.000000 | | | |
| 8 | O | 1.355509 | 4.540081 | 0.000000 | | |
| 9 | O | 2.561790 | 2.807964 | 3.574962 | 0.000000 | |
| 10 | O | 2.466017 | 3.574745 | 2.808074 | 4.551567 | 0.000000 |

U1-centroid: 1.920759Å U2-centroid: 1.921023Å

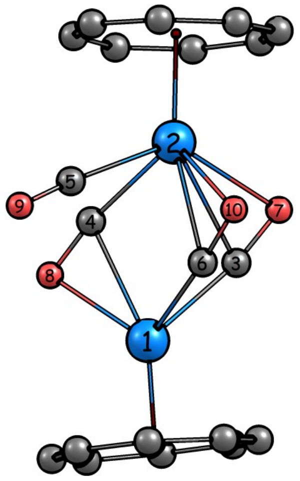


8T. -60569.970159 +13.3

| | | 1 | 2 | 3 | 4 | 5 |
|---|---|---|---|---|---|---|
| 1 | U | 0.000000 | | | | |
| 2 | U | 3.582738 | 0.000000 | | | |
| 3 | C | 2.212738 | 2.568587 | 0.000000 | | |
| 4 | C | 2.464312 | 2.222158 | 2.705004 | 0.000000 | |
| 5 | C | 3.491139 | 2.367802 | 3.936886 | 2.309711 | 0.000000 |
| 6 | C | 2.214496 | 2.539668 | 2.170191 | 2.994362 | 2.770084 |
| 7 | O | 3.425526 | 2.462679 | 1.232374 | 3.374562 | 4.429980 |
| 8 | O | 2.332775 | 3.457486 | 3.357052 | 1.252753 | 3.020711 |
| 9 | O | 3.911656 | 3.534333 | 4.848825 | 3.045984 | 1.170041 |
| 10 | O | 3.430850 | 2.423915 | 2.813486 | 3.701818 | 3.003918 |

| | | 6 | 7 | 8 | 9 | 10 |
|---|---|---|---|---|---|---|
| 6 | C | 0.000000 | | | | |
| 7 | O | 2.822547 | 0.000000 | | | |
| 8 | O | 3.655095 | 4.261853 | 0.000000 | | |
| 9 | O | 3.484432 | 5.485639 | 3.354869 | 0.000000 | |
| 10 | O | 1.237158 | 2.956742 | 4.594184 | 3.832374 | 0.000000 |

U1-centroid: 1.958061Å U2-centroid: 1.937409Å

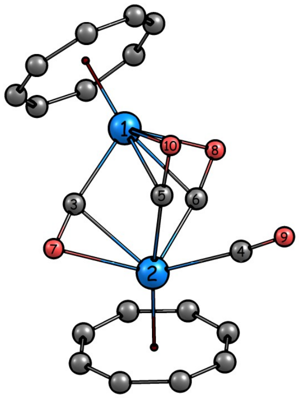


9S. -60569.967612 +14.9

| | | 1 | 2 | 3 | 4 | 5 |
|---|---|---|---|---|---|---|
| 1 | U | 0.000000 | | | | |
| 2 | U | 3.483288 | 0.000000 | | | |
| 3 | C | 2.228031 | 2.468830 | 0.000000 | | |
| 4 | C | 4.198996 | 2.352671 | 4.145201 | 0.000000 | |
| 5 | C | 2.462366 | 2.260253 | 2.333066 | 2.306659 | 0.000000 |
| 6 | C | 2.413068 | 2.179909 | 3.070930 | 2.409883 | 2.284249 |
| 7 | O | 3.449488 | 2.494961 | 1.232790 | 4.422286 | 2.909987 |
| 8 | O | 2.310564 | 3.421704 | 3.799915 | 3.199929 | 2.910562 |
| 9 | O | 4.878560 | 3.515963 | 5.143346 | 1.165901 | 3.046468 |
| 10 | O | 2.347342 | 3.467459 | 3.018975 | 3.077721 | 1.249404 |

| | | 6 | 7 | 8 | 9 | 10 |
|---|---|---|---|---|---|---|
| 6 | C | 0.000000 | | | | |
| 7 | O | 3.861205 | 0.000000 | | | |
| 8 | O | 1.263678 | 4.795175 | 0.000000 | | |
| 9 | O | 3.173685 | 5.490460 | 3.611193 | 0.000000 | |
| 10 | O | 2.868663 | 3.805239 | 2.964368 | 3.447428 | 0.000000 |

U1-centroid: 1.935968Å U2-centroid: 1.989142Å

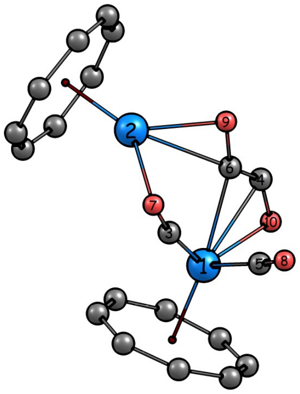


10T. -60569.967037 +15.3

| | | 1 | 2 | 3 | 4 | 5 |
|---|---|---|---|---|---|---|
| 1 | U | 0.000000 | | | | |
| 2 | U | 3.792226 | 0.000000 | | | |
| 3 | C | 2.157044 | 2.541287 | 0.000000 | | |
| 4 | C | 2.454937 | 3.789848 | 3.492549 | 0.000000 | |
| 5 | C | 2.326778 | 4.270959 | 2.188362 | 3.232956 | 0.000000 |
| 6 | C | 2.375911 | 2.594520 | 2.464916 | 1.361526 | 2.727654 |
| 7 | O | 3.406265 | 2.310000 | 1.262064 | 4.351101 | 2.936572 |
| 8 | O | 3.492754 | 4.853207 | 2.926489 | 4.098024 | 1.168897 |
| 9 | O | 3.573660 | 2.182538 | 2.911531 | 2.478166 | 3.402666 |
| 10 | O | 2.327962 | 4.770571 | 4.039912 | 1.266870 | 3.517678 |

| | | 6 | 7 | 8 | 9 | 10 |
|---|---|---|---|---|---|---|
| 6 | C | 0.000000 | | | | |
| 7 | O | 3.101630 | 0.000000 | | | |
| 8 | O | 3.496771 | 3.259463 | 0.000000 | | |
| 9 | O | 1.325021 | 2.994157 | 3.830524 | 0.000000 | |
| 10 | O | 2.466389 | 5.105028 | 4.493184 | 3.702152 | 0.000000 |

U1-centroid: 1.907043Å U2-centroid: 1.964383Å

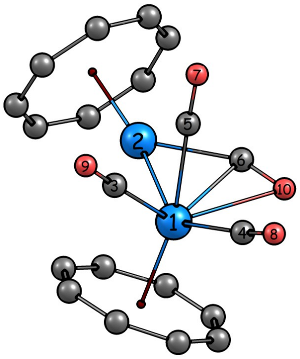

11T. -60569.966572 +15.6

| | | 1 | 2 | 3 | 4 | 5 |
|---|---|---|---|---|---|---|
| 1 | U | 0.000000 | | | | |
| 2 | U | 2.511589 | 0.000000 | | | |
| 3 | C | 2.375205 | 3.662118 | 0.000000 | | |
| 4 | C | 2.424439 | 4.637953 | 2.920560 | 0.000000 | |
| 5 | C | 2.309917 | 2.793045 | 2.258407 | 2.791454 | 0.000000 |
| 6 | C | 2.409998 | 2.260061 | 4.266524 | 3.653166 | 2.684725 |
| 7 | O | 3.489748 | 3.588179 | 2.995311 | 3.560347 | 1.180468 |
| 8 | O | 3.581179 | 5.716127 | 3.706151 | 1.159269 | 3.566428 |
| 9 | O | 3.543283 | 4.569068 | 1.168529 | 3.745437 | 2.998158 |
| 10 | O | 2.629961 | 3.378088 | 4.558780 | 3.092919 | 3.078148 |

| | | 6 | 7 | 8 | 9 | 10 |
|---|---|---|---|---|---|---|
| 6 | C | 0.000000 | | | | |
| 7 | O | 3.470208 | 0.000000 | | | |
| 8 | O | 4.585907 | 4.058849 | 0.000000 | | |
| 9 | O | 5.318880 | 3.366323 | 4.289452 | 0.000000 | |
| 10 | O | 1.216662 | 3.845486 | 3.878794 | 5.625396 | 0.000000 |

U1-centroid: 1.944882Å U2-centroid: 1.999938Å

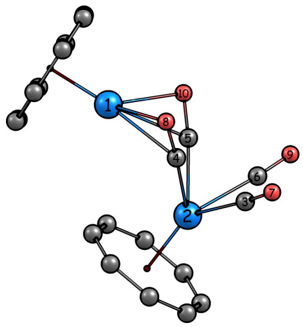

12Q. -60569.965207 +16.5

| | | 1 | 2 | 3 | 4 | 5 |
|---|---|---|---|---|---|---|
| 1 | U | 0.000000 | | | | |
| 2 | U | 3.815415 | 0.000000 | | | |
| 3 | C | 4.811861 | 2.371149 | 0.000000 | | |
| 4 | C | 2.589738 | 2.193218 | 2.274800 | 0.000000 | |
| 5 | C | 2.573666 | 2.187351 | 3.181443 | 1.991865 | 0.000000 |
| 6 | C | 4.773822 | 2.363907 | 2.629901 | 3.238167 | 2.227787 |
| 7 | O | 5.559165 | 3.535205 | 1.165791 | 3.030231 | 4.072769 |
| 8 | O | 2.361220 | 3.436076 | 3.153632 | 1.253399 | 2.691080 |
| 9 | O | 5.511292 | 3.529107 | 3.431904 | 4.145097 | 2.978621 |
| 10 | O | 2.349549 | 3.434596 | 4.123722 | 2.696546 | 1.256108 |

| | | 6 | 7 | 8 | 9 | 10 |
|---|---|---|---|---|---|---|
| 6 | C | 0.000000 | | | | |
| 7 | O | 3.421585 | 0.000000 | | | |
| 8 | O | 4.186248 | 3.561152 | 0.000000 | | |
| 9 | O | 1.166992 | 3.947736 | 4.908774 | 0.000000 | |
| 10 | O | 3.097190 | 4.820505 | 2.852748 | 3.485549 | 0.000000 |

U1-centroid: 1.965043Å U2-centroid: 1.901711Å

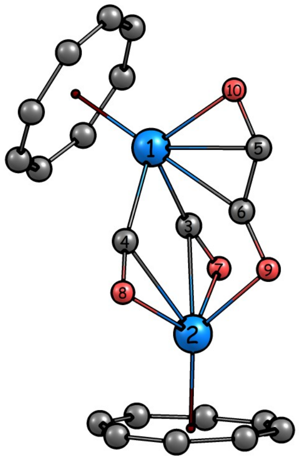


13T. -60569.964464 +16.9

| | | 1 | 2 | 3 | 4 | 5 |
|---|---|---|---|---|---|---|
| 1 | U | 0.000000 | | | | |
| 2 | U | 3.867727 | 0.000000 | | | |
| 3 | C | 2.251415 | 2.582678 | 0.000000 | | |
| 4 | C | 2.242464 | 2.577692 | 2.812995 | 0.000000 | |
| 5 | C | 2.438019 | 4.153044 | 3.278726 | 3.296881 | 0.000000 |
| 6 | C | 2.381267 | 2.805748 | 2.473695 | 2.452459 | 1.356623 |
| 7 | O | 3.458756 | 2.441515 | 1.223801 | 3.673324 | 4.038247 |
| 8 | O | 3.453438 | 2.418611 | 3.665967 | 1.227001 | 4.066018 |
| 9 | O | 3.551507 | 2.236762 | 2.983544 | 2.955216 | 2.473021 |
| 10 | O | 2.264799 | 5.113907 | 3.748812 | 3.779692 | 1.284709 |

| | | 6 | 7 | 8 | 9 | 10 |
|---|---|---|---|---|---|---|
| 6 | C | 0.000000 | | | | |
| 7 | O | 3.086378 | 0.000000 | | | |
| 8 | O | 3.061642 | 4.253392 | 0.000000 | | |
| 9 | O | 1.305148 | 3.128105 | 3.083710 | 0.000000 | |
| 10 | O | 2.467585 | 4.700166 | 4.745035 | 3.703575 | 0.000000 |

U1-centroid: 1.985610Å U2-centroid: 1.933185Å

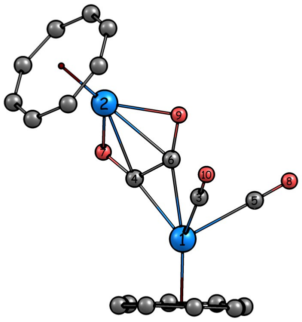


14Q. -60569.964219 +17.1

| | | 1 | 2 | 3 | 4 | 5 |
|---|---|---|---|---|---|---|
| 1 | U | 0.000000 | | | | |
| 2 | U | 4.585142 | 0.000000 | | | |
| 3 | C | 2.344930 | 4.174420 | 0.000000 | | |
| 4 | C | 2.320846 | 2.560596 | 3.182738 | 0.000000 | |
| 5 | C | 2.387927 | 5.247666 | 2.696774 | 3.571894 | 0.000000 |
| 6 | C | 2.351922 | 2.552807 | 2.225879 | 1.423175 | 2.735022 |
| 7 | O | 3.638805 | 2.163234 | 4.414150 | 1.358708 | 4.737067 |
| 8 | O | 3.549649 | 5.887391 | 3.490230 | 4.517202 | 1.161851 |
| 9 | O | 3.639762 | 2.195785 | 2.893967 | 2.466909 | 3.384477 |
| 10 | O | 3.513541 | 4.383028 | 1.169853 | 4.037589 | 3.485942 |

| | | 6 | 7 | 8 | 9 | 10 |
|---|---|---|---|---|---|---|
| 6 | C | 0.000000 | | | | |
| 7 | O | 2.436655 | 0.000000 | | | |
| 8 | O | 3.531135 | 5.563479 | 0.000000 | | |
| 9 | O | 1.332909 | 2.953226 | 3.818290 | 0.000000 | |
| 10 | O | 2.918623 | 5.122089 | 4.007966 | 3.112400 | 0.000000 |

U1-centroid: 1.905517Å U2-centroid: 1.937164Å

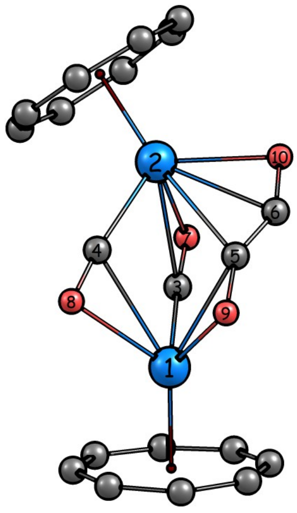


15Q. -60569.963945 +17.2

| | | 1 | 2 | 3 | 4 | 5 |
|---|---|---|---|---|---|---|
| 1 | U | 0.000000 | | | | |
| 2 | U | 3.905621 | 0.000000 | | | |
| 3 | C | 2.291735 | 2.627611 | 0.000000 | | |
| 4 | C | 2.628097 | 2.278481 | 2.773070 | 0.000000 | |
| 5 | C | 2.614714 | 2.805952 | 2.973270 | 2.640868 | 0.000000 |
| 6 | C | 3.543756 | 2.602295 | 3.092470 | 3.428106 | 1.301142 |
| 7 | O | 3.503639 | 2.436196 | 1.223033 | 3.448132 | 3.623681 |
| 8 | O | 2.461780 | 3.492980 | 3.444076 | 1.219300 | 3.192151 |
| 9 | O | 2.246554 | 3.882665 | 3.574161 | 2.944776 | 1.295810 |
| 10 | O | 4.470110 | 2.332459 | 3.368039 | 3.952935 | 2.467602 |

| | | 6 | 7 | 8 | 9 | 10 |
|---|---|---|---|---|---|---|
| 6 | C | 0.000000 | | | | |
| 7 | O | 3.321414 | 0.000000 | | | |
| 8 | O | 4.248498 | 4.347593 | 0.000000 | | |
| 9 | O | 2.520339 | 4.496220 | 2.974212 | 0.000000 | |
| 10 | O | 1.264828 | 3.120333 | 4.981883 | 3.743803 | 0.000000 |

U1-centroid: 1.944010Å U2-centroid: 1.958298Å

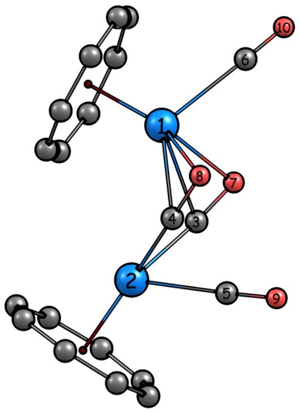


16T. -60569.963409 +17.6

| | | 1 | 2 | 3 | 4 | 5 |
|---|---|---|---|---|---|---|
| 1 | U | 0.000000 | | | | |
| 2 | U | 3.592711 | 0.000000 | | | |
| 3 | C | 2.500141 | 2.166362 | 0.000000 | | |
| 4 | C | 2.491387 | 2.152953 | 2.259185 | 0.000000 | |
| 5 | C | 4.130165 | 2.256689 | 2.245937 | 2.260646 | 0.000000 |
| 6 | C | 2.474252 | 5.680618 | 3.896531 | 4.352244 | 5.430090 |
| 7 | O | 2.386297 | 3.399817 | 1.253040 | 2.921361 | 3.001848 |
| 8 | O | 2.310046 | 3.394343 | 2.901495 | 1.266475 | 3.047928 |
| 9 | O | 4.790144 | 3.430488 | 3.021632 | 3.042303 | 1.174285 |
| 10 | O | 3.631532 | 6.755302 | 4.862994 | 5.406307 | 6.301938 |

| | | 6 | 7 | 8 | 9 | 10 |
|---|---|---|---|---|---|---|
| 6 | C | 0.000000 | | | | |
| 7 | O | 2.913020 | 0.000000 | | | |
| 8 | O | 3.631500 | 3.050117 | 0.000000 | | |
| 9 | O | 5.623425 | 3.392293 | 3.455309 | 0.000000 | |
| 10 | O | 1.157549 | 3.750968 | 4.603454 | 6.336547 | 0.000000 |

U1-centroid: 1.970861Å U2-centroid: 1.948747Å

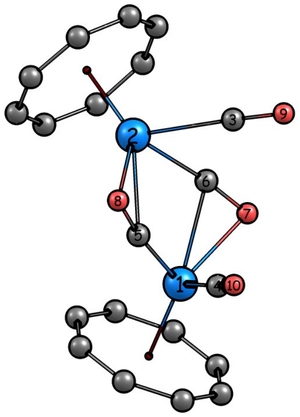

17T. -60569.962636 +18.1

| | | 1 | 2 | 3 | 4 | 5 |
|---|---|---|---|---|---|---|
| 1 | U | 0.000000 | | | | |
| 2 | U | 3.588621 | 0.000000 | | | |
| 3 | C | 4.105814 | 2.338742 | 0.000000 | | |
| 4 | C | 2.337505 | 4.112916 | 3.836699 | 0.000000 | |
| 5 | C | 2.192580 | 2.398874 | 3.367542 | 2.179432 | 0.000000 |
| 6 | C | 2.398745 | 2.195721 | 2.187961 | 3.384298 | 2.716544 |
| 7 | O | 2.299044 | 3.454539 | 2.971160 | 3.385740 | 3.437841 |
| 8 | O | 3.451932 | 2.299551 | 3.371377 | 2.966309 | 1.267596 |
| 9 | O | 4.747337 | 3.507055 | 1.169800 | 4.185962 | 4.258404 |
| 10 | O | 3.506107 | 4.756324 | 4.189678 | 1.170142 | 2.924153 |

| | | 6 | 7 | 8 | 9 | 10 |
|---|---|---|---|---|---|---|
| 6 | C | 0.000000 | | | | |
| 7 | O | 1.267619 | 0.000000 | | | |
| 8 | O | 3.441353 | 4.371102 | 0.000000 | | |
| 9 | O | 2.933080 | 3.310461 | 4.311332 | 0.000000 | |
| 10 | O | 4.278831 | 4.329204 | 3.304260 | 4.362528 | 0.000000 |

U1-centroid: 1.943910Å U2-centroid: 1.943214Å

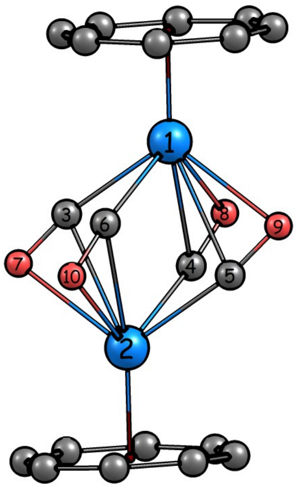

18Q. -60569.962532 +18.1

| | | 1 | 2 | 3 | 4 | 5 |
|---|---|---|---|---|---|---|
| 1 | U | 0.000000 | | | | |
| 2 | U | 3.687236 | 0.000000 | | | |
| 3 | C | 2.281729 | 2.623479 | 0.000000 | | |
| 4 | C | 2.624875 | 2.282171 | 2.506102 | 0.000000 | |
| 5 | C | 2.623217 | 2.281061 | 3.252340 | 2.073537 | 0.000000 |
| 6 | C | 2.280235 | 2.623123 | 2.071120 | 3.253118 | 2.508159 |
| 7 | O | 3.487995 | 2.514760 | 1.223005 | 3.092352 | 3.934727 |
| 8 | O | 2.519004 | 3.488024 | 3.094091 | 1.222336 | 2.723403 |
| 9 | O | 2.517826 | 3.487453 | 3.937167 | 2.724028 | 1.222620 |
| 10 | O | 3.486789 | 2.516714 | 2.724427 | 3.937301 | 3.093931 |

| | | 6 | 7 | 8 | 9 | 10 |
|---|---|---|---|---|---|---|
| 6 | C | 0.000000 | | | | |
| 7 | O | 2.720983 | 0.000000 | | | |
| 8 | O | 3.936866 | 3.902930 | 0.000000 | | |
| 9 | O | 3.097140 | 4.836959 | 2.857038 | 0.000000 | |
| 10 | O | 1.222685 | 2.857284 | 4.838132 | 3.904371 | 0.000000 |

U1-centroid: 1.935018Å U2-centroid: 1.934705Å

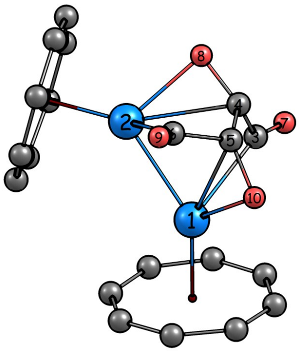


19S. -60569.959774 +19.9

| | | 1 | 2 | 3 | 4 | 5 |
|---|---|---|---|---|---|---|
| 1 | U | 0.000000 | | | | |
| 2 | U | 2.583561 | 0.000000 | | | |
| 3 | C | 2.455240 | 3.021897 | 0.000000 | | |
| 4 | C | 2.744292 | 2.498897 | 1.489383 | 0.000000 | |
| 5 | C | 2.502494 | 2.744573 | 2.526341 | 1.445941 | 0.000000 |
| 6 | C | 3.038554 | 2.461254 | 3.704991 | 2.522084 | 1.488974 |
| 7 | O | 3.481876 | 3.994674 | 1.211078 | 2.491106 | 3.666602 |
| 8 | O | 3.594295 | 2.182981 | 2.376428 | 1.359155 | 2.412958 |
| 9 | O | 4.019324 | 3.488262 | 4.881440 | 3.660407 | 2.491596 |
| 10 | O | 2.183992 | 3.590647 | 2.844487 | 2.413845 | 1.357636 |

| | | 6 | 7 | 8 | 9 | 10 |
|---|---|---|---|---|---|---|
| 6 | C | 0.000000 | | | | |
| 7 | O | 4.879834 | 0.000000 | | | |
| 8 | O | 2.833779 | 3.098265 | 0.000000 | | |
| 9 | O | 1.210672 | 6.053657 | 3.902852 | 0.000000 | |
| 10 | O | 2.378658 | 3.920309 | 3.618932 | 3.106854 | 0.000000 |

U1-centroid: 1.926115Å U2-centroid: 1.920249Å

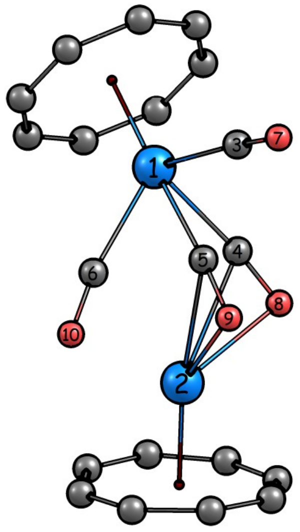


20Q. -60569.957676 +21.2

| | | 1 | 2 | 3 | 4 | 5 |
|---|---|---|---|---|---|---|
| 1 | U | 0.000000 | | | | |
| 2 | U | 3.914103 | 0.000000 | | | |
| 3 | C | 2.384099 | 4.829936 | 0.000000 | | |
| 4 | C | 2.256963 | 2.624202 | 3.199999 | 0.000000 | |
| 5 | C | 2.209764 | 2.619408 | 2.253597 | 2.007163 | 0.000000 |
| 6 | C | 2.287518 | 2.683998 | 3.650471 | 2.976161 | 2.052876 |
| 7 | O | 3.546878 | 5.548920 | 1.164322 | 4.077644 | 3.004373 |
| 8 | O | 3.486372 | 2.409269 | 4.112124 | 1.237070 | 2.709208 |
| 9 | O | 3.457428 | 2.358777 | 3.119569 | 2.710236 | 1.255332 |
| 10 | O | 3.500050 | 2.490853 | 4.604249 | 3.775880 | 2.735525 |

| | | 6 | 7 | 8 | 9 | 10 |
|---|---|---|---|---|---|---|
| 6 | C | 0.000000 | | | | |
| 7 | O | 4.598394 | 0.000000 | | | |
| 8 | O | 3.794176 | 4.791200 | 0.000000 | | |
| 9 | O | 2.762580 | 3.511895 | 2.884823 | 0.000000 | |
| 10 | O | 1.223546 | 5.411436 | 4.305176 | 2.933477 | 0.000000 |

U1-centroid: 1.908839Å U2-centroid: 1.956626Å

Table S4: Distance table for the lowest-lying $U_2COT_2(CO)_5$ optimized structures obtained at the BP86/def2-TZVP/ZORA level of theory. Included are the zero-point corrected absolute energies in (a.u.) and relative energies in (kcal/mol). The hydrogen atoms of the cyclooctatetraene rings are omitted for clarity. S, T, Q and H denote the singlet, triplet, quintet and septet spin states respectively.

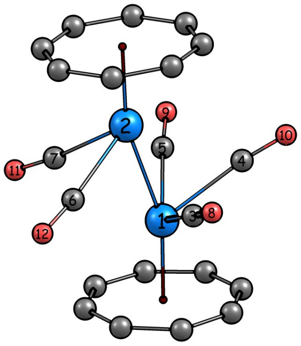


1S. -60683.339751 0.0

| | | 1 | 2 | 3 | 4 | 5 |
|---|---|---|---|---|---|---|
| 1 | U | 0.000000 | | | | |
| 2 | U | 2.559595 | 0.000000 | | | |
| 3 | C | 2.346294 | 2.846926 | 0.000000 | | |
| 4 | C | 2.309793 | 2.904144 | 2.621808 | 0.000000 | |
| 5 | C | 2.346900 | 2.859503 | 4.238867 | 2.637714 | 0.000000 |
| 6 | C | 2.898544 | 2.306096 | 2.795611 | 4.362937 | 4.348915 |
| 7 | C | 2.892992 | 2.303229 | 4.334254 | 4.364582 | 2.804081 |
| 8 | O | 3.518725 | 3.621174 | 1.174379 | 3.383941 | 5.307879 |
| 9 | O | 3.518889 | 3.651523 | 5.316461 | 3.412950 | 1.173859 |
| 10 | O | 3.478092 | 3.763391 | 3.431160 | 1.174252 | 3.454629 |
| 11 | O | 3.742139 | 3.473417 | 5.368657 | 5.398882 | 3.496501 |
| 12 | O | 3.747564 | 3.476295 | 3.498278 | 5.398657 | 5.379083 |

| | | 6 | 7 | 8 | 9 | 10 |
|---|---|---|---|---|---|---|
| 6 | C | 0.000000 | | | | |
| 7 | C | 2.597457 | 0.000000 | | | |
| 8 | O | 3.471352 | 5.331648 | 0.000000 | | |
| 9 | O | 5.354859 | 3.486633 | 6.332074 | 0.000000 | |
| 10 | O | 5.397156 | 5.402890 | 3.904728 | 3.948801 | 0.000000 |
| 11 | O | 3.406455 | 1.175115 | 6.385722 | 4.000344 | 6.447233 |
| 12 | O | 1.175112 | 3.399887 | 4.003170 | 6.401887 | 6.442536 |

| | | 11 | 12 |
|---|---|---|---|
| 11 | O | 0.000000 | |
| 12 | O | 3.927187 | 0.000000 |

U1-centroid: 1.966550Å U2-centroid: 1.943684Å

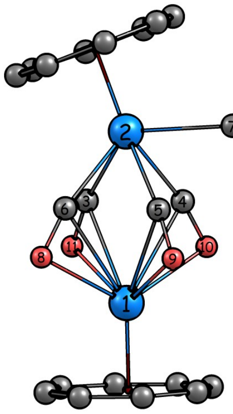


2T. -60683.333356 +4.0

| | | 1 | 2 | 3 | 4 | 5 |
|---|---|---|---|---|---|---|
| 1 | U | 0.000000 | | | | |
| 2 | U | 3.793489 | 0.000000 | | | |
| 3 | C | 2.668790 | 2.260185 | 0.000000 | | |
| 4 | C | 2.675556 | 2.259198 | 2.147860 | 0.000000 | |
| 5 | C | 2.678328 | 2.259851 | 3.129789 | 2.411396 | 0.000000 |
| 6 | C | 2.662243 | 2.257653 | 2.140835 | 3.120461 | 2.143549 |
| 7 | C | 4.631826 | 2.419790 | 4.124173 | 2.579401 | 2.567874 |
| 8 | O | 2.461901 | 3.467445 | 2.843266 | 3.906326 | 2.806877 |
| 9 | O | 2.493839 | 3.464985 | 3.916828 | 3.110036 | 1.222331 |
| 10 | O | 2.491473 | 3.464529 | 2.838386 | 1.222225 | 3.111003 |
| 11 | O | 2.465768 | 3.468585 | 1.227994 | 2.807450 | 3.912244 |
| 12 | O | 5.318547 | 3.579002 | 5.154980 | 3.352659 | 3.337719 |
| | | 6 | 7 | 8 | 9 | 10 |
| 6 | C | 0.000000 | | | | |
| 7 | C | 4.113398 | 0.000000 | | | |
| 8 | O | 1.228279 | 5.127806 | 0.000000 | | |
| 9 | O | 2.833571 | 3.427985 | 3.010344 | 0.000000 | |
| 10 | O | 3.907783 | 3.439681 | 4.410903 | 3.392794 | 0.000000 |
| 11 | O | 2.841839 | 5.136619 | 3.069737 | 4.416542 | 3.011607 |
| 12 | O | 5.141748 | 1.161696 | 6.065776 | 3.907129 | 3.924455 |
| | | 11 | 12 | | | |
| 11 | O | 0.000000 | | | | |
| 12 | O | 6.077665 | 0.000000 | | | |

U1-centroid: 1.993477Å U2-centroid: 1.928855Å

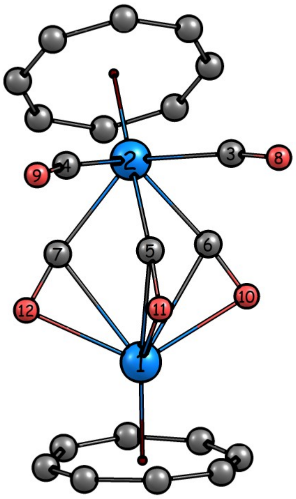


3T. -60683.329938 +6.2

| | | 1 | 2 | 3 | 4 | 5 |
|---|---|---|---|---|---|---|
| 1 | U | 0.000000 | | | | |
| 2 | U | 3.850219 | 0.000000 | | | |
| 3 | C | 4.655916 | 2.418499 | 0.000000 | | |
| 4 | C | 4.633996 | 2.416119 | 3.185115 | 0.000000 | |
| 5 | C | 2.650741 | 2.195767 | 2.399996 | 2.394637 | 0.000000 |
| 6 | C | 2.597952 | 2.257751 | 2.747560 | 4.090634 | 2.219290 |
| 7 | C | 2.592850 | 2.257102 | 4.098636 | 2.728398 | 2.226169 |
| 8 | O | 5.297057 | 3.573874 | 1.160819 | 4.025628 | 3.135611 |
| 9 | O | 5.267605 | 3.571719 | 4.026625 | 1.161143 | 3.129184 |
| 10 | O | 2.406602 | 3.473479 | 3.510653 | 5.074475 | 2.872874 |
| 11 | O | 2.386889 | 3.426762 | 3.262203 | 3.253739 | 1.245763 |
| 12 | O | 2.408042 | 3.472883 | 5.084923 | 3.481894 | 2.881624 |
| | | 6 | 7 | 8 | 9 | 10 |
| 6 | C | 0.000000 | | | | |
| 7 | C | 2.910307 | 0.000000 | | | |
| 8 | O | 3.530805 | 5.100458 | 0.000000 | | |
| 9 | O | 5.090746 | 3.507911 | 4.666023 | 0.000000 | |
| 10 | O | 1.233213 | 3.729054 | 3.993195 | 5.966946 | 0.000000 |
| 11 | O | 2.906890 | 2.913425 | 3.663977 | 3.652151 | 3.075399 |
| 12 | O | 3.735159 | 1.232982 | 5.980519 | 3.954731 | 4.263507 |
| | | 11 | 12 | | | |
| 11 | O | 0.000000 | | | | |
| 12 | O | 3.086416 | 0.000000 | | | |

U1-centroid: 1.983435Å U2-centroid: 1.917234Å

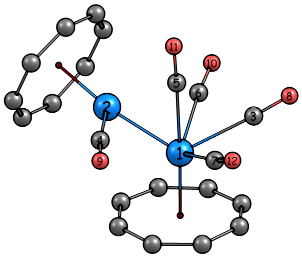


4S. -60683.329639 +6.3

| | | 1 | 2 | 3 | 4 | 5 |
|---|---|---|---|---|---|---|
| 1 | U | 0.000000 | | | | |
| 2 | U | 2.519455 | 0.000000 | | | |
| 3 | C | 2.346620 | 4.256879 | 0.000000 | | |
| 4 | C | 2.713698 | 2.295733 | 4.572500 | 0.000000 | |
| 5 | C | 2.290434 | 2.746537 | 2.548996 | 2.725009 | 0.000000 |
| 6 | C | 2.302577 | 2.761139 | 2.398317 | 4.258524 | 2.704274 |
| 7 | C | 2.436338 | 4.439346 | 2.588213 | 3.385350 | 2.662767 |
| 8 | O | 3.513755 | 5.271207 | 1.168654 | 5.632074 | 3.330752 |
| 9 | O | 3.366336 | 3.471381 | 5.057669 | 1.181831 | 3.364210 |
| 10 | O | 3.480303 | 3.588475 | 3.098674 | 5.310200 | 3.546113 |
| 11 | O | 3.471013 | 3.473045 | 3.351237 | 3.385979 | 1.181070 |
| 12 | O | 3.593681 | 5.483201 | 3.334507 | 4.198128 | 3.429001 |

| | | 6 | 7 | 8 | 9 | 10 |
|---|---|---|---|---|---|---|
| 6 | C | 0.000000 | | | | |
| 7 | C | 4.169845 | 0.000000 | | | |
| 8 | O | 3.129445 | 3.381276 | 0.000000 | | |
| 9 | O | 5.203996 | 3.257776 | 6.073907 | 0.000000 | |
| 10 | O | 1.180860 | 5.205150 | 3.469033 | 6.296175 | 0.000000 |
| 11 | O | 3.517941 | 3.457698 | 3.837762 | 3.921630 | 4.100058 |
| 12 | O | 5.188700 | 1.160255 | 3.832915 | 3.822141 | 6.149565 |

| | | 11 | 12 |
|---|---|---|---|
| 11 | O | 0.000000 | |
| 12 | O | 3.938700 | 0.000000 |

U1-centroid: 1.941880Å U2-centroid: 1.995124Å

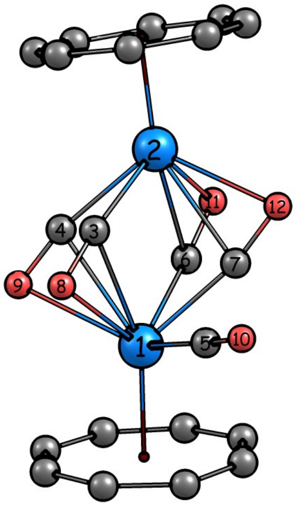


5S. -60683.327400 +7.8

| | | 1 | 2 | 3 | 4 | 5 |
|---|---|---|---|---|---|---|
| 1 | U | 0.000000 | | | | |
| 2 | U | 3.425287 | 0.000000 | | | |
| 3 | C | 2.593794 | 2.268562 | 0.000000 | | |
| 4 | C | 2.541477 | 2.249367 | 2.179424 | 0.000000 | |
| 5 | C | 2.444948 | 4.090434 | 2.835295 | 4.265664 | 0.000000 |
| 6 | C | 2.289716 | 2.511283 | 3.406169 | 2.399092 | 3.991692 |
| 7 | C | 2.228391 | 2.464553 | 2.695505 | 3.283193 | 2.258823 |
| 8 | O | 2.595123 | 3.457302 | 1.218502 | 2.775794 | 2.695494 |
| 9 | O | 2.522015 | 3.446854 | 2.787277 | 1.224436 | 4.537787 |
| 10 | O | 3.601050 | 4.785608 | 3.596152 | 5.283128 | 1.159934 |
| 11 | O | 3.485704 | 2.479834 | 4.097307 | 3.029414 | 4.901889 |
| 12 | O | 3.448995 | 2.429993 | 3.309405 | 3.997171 | 3.090639 |

| | | 6 | 7 | 8 | 9 | 10 |
|---|---|---|---|---|---|---|
| 6 | C | 0.000000 | | | | |
| 7 | C | 2.167497 | 0.000000 | | | |
| 8 | O | 4.112755 | 3.380045 | 0.000000 | | |
| 9 | O | 3.035764 | 4.003218 | 2.865457 | 0.000000 | |
| 10 | O | 4.971356 | 2.990679 | 3.468773 | 5.629725 | 0.000000 |
| 11 | O | 1.227657 | 2.776118 | 5.007736 | 3.881763 | 5.756732 |
| 12 | O | 2.829445 | 1.240115 | 4.202861 | 4.925453 | 3.466012 |

| | | 11 | 12 |
|---|---|---|---|
| 11 | O | 0.000000 | |
| 12 | O | 2.922306 | 0.000000 |

U1-centroid: 1.999638Å U2-centroid: 1.947808Å

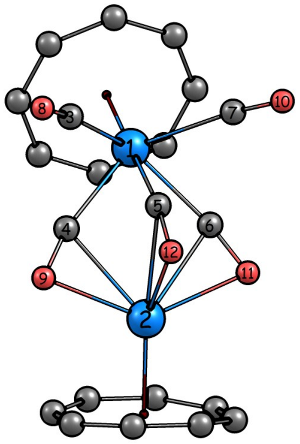


6S. -60683.327017 +8.0

| | | 1 | 2 | 3 | 4 | 5 |
|---|---|---|---|---|---|---|
| 1 | U | 0.000000 | | | | |
| 2 | U | 3.546433 | 0.000000 | | | |
| 3 | C | 2.433895 | 4.537909 | 0.000000 | | |
| 4 | C | 2.237325 | 2.429600 | 2.566884 | 0.000000 | |
| 5 | C | 2.206026 | 2.524283 | 2.535521 | 2.154859 | 0.000000 |
| 6 | C | 2.237488 | 2.430570 | 4.125105 | 3.029465 | 2.153704 |
| 7 | C | 2.434311 | 4.542638 | 3.424113 | 4.120564 | 2.527015 |
| 8 | O | 3.587367 | 5.251081 | 1.158883 | 3.320352 | 3.291405 |
| 9 | O | 3.465925 | 2.325520 | 3.356734 | 1.255408 | 2.767193 |
| 10 | O | 3.587905 | 5.258749 | 4.316897 | 5.134238 | 3.282607 |
| 11 | O | 3.466453 | 2.325020 | 5.109379 | 3.823527 | 2.767429 |
| 12 | O | 3.426859 | 2.337133 | 3.451041 | 2.805547 | 1.252603 |

| | | 6 | 7 | 8 | 9 | 10 |
|---|---|---|---|---|---|---|
| 6 | C | 0.000000 | | | | |
| 7 | C | 2.580791 | 0.000000 | | | |
| 8 | O | 5.139030 | 4.317607 | 0.000000 | | |
| 9 | O | 3.824279 | 5.102602 | 3.782099 | 0.000000 | |
| 10 | O | 3.337844 | 1.158831 | 5.051152 | 6.008648 | 0.000000 |
| 11 | O | 1.255647 | 3.374088 | 6.016214 | 4.306335 | 3.806760 |
| 12 | O | 2.804223 | 3.442961 | 3.916144 | 2.880197 | 3.906896 |

| | | 11 | 12 |
|---|---|---|---|
| 11 | O | 0.000000 | |
| 12 | O | 2.879925 | 0.000000 |

U1-centroid: 1.954742Å U2-centroid: 1.991558Å

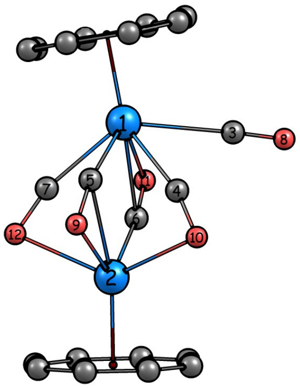


7S. -60683.326653 +8.2

| | | 1 | 2 | 3 | 4 | 5 |
|---|---|---|---|---|---|---|
| 1 | U | 0.000000 | | | | |
| 2 | U | 3.479296 | 0.000000 | | | |
| 3 | C | 2.494871 | 4.308538 | 0.000000 | | |
| 4 | C | 2.227861 | 2.500566 | 2.395533 | 0.000000 | |
| 5 | C | 2.216271 | 2.515345 | 3.964165 | 2.017995 | 0.000000 |
| 6 | C | 2.544451 | 2.325246 | 3.014940 | 2.716207 | 3.308775 |
| 7 | C | 2.255146 | 2.520521 | 4.347954 | 3.163404 | 2.050257 |
| 8 | O | 3.647166 | 5.030920 | 1.157102 | 3.138963 | 4.941440 |
| 9 | O | 3.436879 | 2.366543 | 4.935910 | 2.672221 | 1.255520 |
| 10 | O | 3.443802 | 2.403934 | 3.245628 | 1.241056 | 2.688979 |
| 11 | O | 2.615685 | 3.523555 | 2.852757 | 3.428581 | 4.068366 |
| 12 | O | 3.461703 | 2.416286 | 5.372811 | 3.920622 | 2.695040 |

| | | 6 | 7 | 8 | 9 | 10 |
|---|---|---|---|---|---|---|
| 6 | C | 0.000000 | | | | |
| 7 | C | 2.442468 | 0.000000 | | | |
| 8 | O | 3.821235 | 5.424981 | 0.000000 | | |
| 9 | O | 3.970538 | 2.708281 | 5.798397 | 0.000000 | |
| 10 | O | 3.327547 | 3.939667 | 3.666064 | 2.789170 | 0.000000 |
| 11 | O | 1.210410 | 3.027932 | 3.649253 | 4.934325 | 4.233561 |
| 12 | O | 3.023012 | 1.237711 | 6.380840 | 2.802652 | 4.397269 |

| | | 11 | 12 |
|---|---|---|---|
| 11 | O | 0.000000 | |
| 12 | O | 3.795448 | 0.000000 |

U1-centroid: 1.990218Å U2-centroid: 1.959894Å

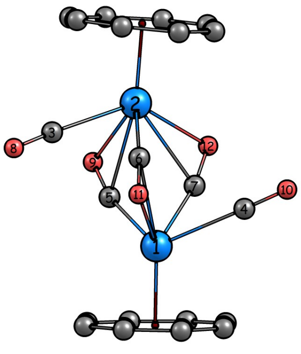

8T. -60683.325968 +8.6

| | | 1 | 2 | 3 | 4 | 5 |
|---|---|---|---|---|---|---|
| 1 | U | 0.000000 | | | | |
| 2 | U | 3.618104 | 0.000000 | | | |
| 3 | C | 3.821204 | 2.407343 | 0.000000 | | |
| 4 | C | 2.433557 | 3.943172 | 5.102039 | 0.000000 | |
| 5 | C | 2.244364 | 2.548350 | 2.829993 | 3.952219 | 0.000000 |
| 6 | C | 2.491463 | 2.245751 | 2.368643 | 2.921127 | 2.904210 |
| 7 | C | 2.200960 | 2.587895 | 4.111691 | 2.370569 | 2.122777 |
| 8 | O | 4.361356 | 3.567801 | 1.163390 | 5.876322 | 3.588496 |
| 9 | O | 3.455850 | 2.427266 | 2.925314 | 4.867471 | 1.235693 |
| 10 | O | 3.586994 | 4.518218 | 5.968239 | 1.161067 | 4.927281 |
| 11 | O | 2.413330 | 3.471593 | 3.085349 | 2.967347 | 3.567501 |
| 12 | O | 3.416747 | 2.465936 | 4.520061 | 3.166667 | 2.783116 |

| | | 6 | 7 | 8 | 9 | 10 |
|---|---|---|---|---|---|---|
| 6 | C | 0.000000 | | | | |
| 7 | C | 2.900076 | 0.000000 | | | |
| 8 | O | 3.104006 | 5.073584 | 0.000000 | | |
| 9 | O | 3.611917 | 2.745508 | 3.770089 | 0.000000 | |
| 10 | O | 3.739975 | 3.091467 | 6.804773 | 5.711064 | 0.000000 |
| 11 | O | 1.237467 | 3.603391 | 3.442235 | 4.495381 | 3.860377 |
| 12 | O | 3.574139 | 1.232741 | 5.604374 | 2.882986 | 3.538614 |

| | | 11 | 12 |
|---|---|---|---|
| 11 | O | 0.000000 | |
| 12 | O | 4.500660 | 0.000000 |

U1-centroid: 1.963827Å U2-centroid: 1.954466Å

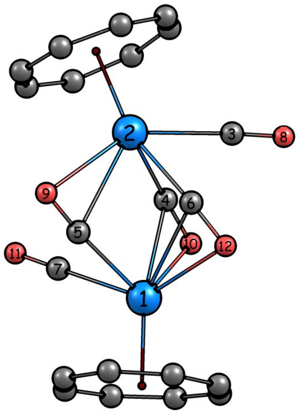

9T. -60683.325764 +8.8

| | | 1 | 2 | 3 | 4 | 5 |
|---|---|---|---|---|---|---|
| 1 | U | 0.000000 | | | | |
| 2 | U | 3.681064 | 0.000000 | | | |
| 3 | C | 4.136360 | 2.300429 | 0.000000 | | |
| 4 | C | 2.542507 | 2.191734 | 2.281769 | 0.000000 | |
| 5 | C | 2.274358 | 2.609997 | 4.225937 | 3.013662 | 0.000000 |
| 6 | C | 2.569453 | 2.234079 | 2.203047 | 2.328031 | 2.581008 |
| 7 | C | 2.406258 | 3.719022 | 4.983090 | 2.828649 | 2.471077 |
| 8 | O | 4.749877 | 3.470168 | 1.170683 | 3.045914 | 5.224338 |
| 9 | O | 3.480579 | 2.615717 | 4.642134 | 3.756344 | 1.209927 |
| 10 | O | 2.386489 | 3.414924 | 3.003726 | 1.244908 | 3.717091 |
| 11 | O | 3.568164 | 4.230502 | 5.717153 | 3.599860 | 3.229191 |
| 12 | O | 2.457099 | 3.442637 | 2.898460 | 2.953499 | 3.277877 |

| | | 6 | 7 | 8 | 9 | 10 |
|---|---|---|---|---|---|---|
| 6 | C | 0.000000 | | | | |
| 7 | C | 4.041923 | 0.000000 | | | |
| 8 | O | 2.948295 | 5.839646 | 0.000000 | | |
| 9 | O | 3.238749 | 3.234387 | 5.740123 | 0.000000 | |
| 10 | O | 2.951860 | 3.024008 | 3.380669 | 4.670743 | 0.000000 |
| 11 | O | 5.002790 | 1.163356 | 6.625944 | 3.659619 | 3.895114 |
| 12 | O | 1.234236 | 4.513966 | 3.233468 | 4.139080 | 3.085137 |

| | | 11 | 12 |
|---|---|---|---|
| 11 | O | 0.000000 | |
| 12 | O | 5.600232 | 0.000000 |

U1-centroid: 1.964117Å U2-centroid: 1.982469Å

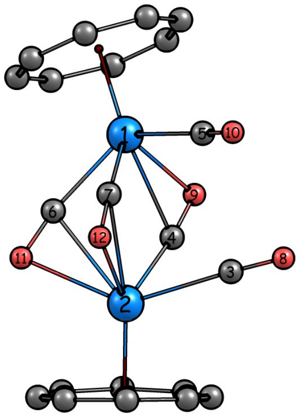

10T. -60683.324656 +9.5

| | | 1 | 2 | 3 | 4 | 5 |
|---|---|---|---|---|---|---|
| 1 | U | 0.000000 | | | | |
| 2 | U | 3.671634 | 0.000000 | | | |
| 3 | C | 3.890162 | 2.394448 | 0.000000 | | |
| 4 | C | 2.512225 | 2.250541 | 2.431720 | 0.000000 | |
| 5 | C | 2.427229 | 4.108049 | 3.051591 | 3.396962 | 0.000000 |
| 6 | C | 2.235160 | 2.603789 | 4.181699 | 2.696424 | 3.994874 |
| 7 | C | 2.200055 | 2.563851 | 3.118057 | 2.962686 | 2.378504 |
| 8 | O | 4.496071 | 3.554015 | 1.163976 | 3.191003 | 3.194312 |
| 9 | O | 2.407396 | 3.478633 | 3.205781 | 1.237215 | 3.444945 |
| 10 | O | 3.578213 | 4.742243 | 3.323909 | 4.316629 | 1.161271 |
| 11 | O | 3.441227 | 2.484784 | 4.592443 | 3.378262 | 4.937894 |
| 12 | O | 3.427761 | 2.389124 | 3.185335 | 3.641268 | 3.213364 |
| | | 6 | 7 | 8 | 9 | 10 |
| 6 | C | 0.000000 | | | | |
| 7 | C | 2.139889 | 0.000000 | | | |
| 8 | O | 5.188527 | 3.977990 | 0.000000 | | |
| 9 | O | 3.346321 | 3.651959 | 3.626066 | 0.000000 | |
| 10 | O | 4.986992 | 3.107095 | 3.198415 | 4.432836 | 0.000000 |
| 11 | O | 1.225366 | 2.775038 | 5.706827 | 4.252601 | 5.815247 |
| 12 | O | 2.808597 | 1.244881 | 4.121378 | 4.560996 | 3.607944 |
| | | 11 | 12 | | | |
| 11 | O | 0.000000 | | | | |
| 12 | O | 2.930424 | 0.000000 | | | |

U1-centroid: 1.960124Å U2-centroid: 1.966241Å

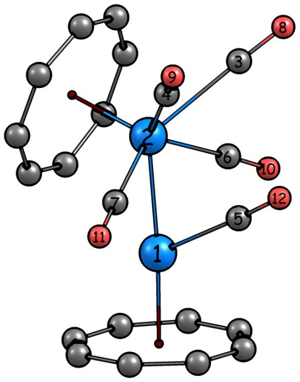

11S. -60683.321643 +11.4

| | | 1 | 2 | 3 | 4 | 5 |
|---|---|---|---|---|---|---|
| 1 | U | 0.000000 | | | | |
| 2 | U | 2.493923 | 0.000000 | | | |
| 3 | C | 4.363868 | 2.442027 | 0.000000 | | |
| 4 | C | 4.332589 | 2.442382 | 2.744932 | 0.000000 | |
| 5 | C | 2.284123 | 2.659326 | 3.347013 | 3.301701 | 0.000000 |
| 6 | C | 2.684240 | 2.327450 | 2.574425 | 4.240140 | 2.735308 |
| 7 | C | 2.687069 | 2.328217 | 4.247857 | 2.534081 | 2.743596 |
| 8 | O | 5.392768 | 3.601498 | 1.161222 | 3.576312 | 4.154160 |
| 9 | O | 5.356701 | 3.601560 | 3.582000 | 1.161203 | 4.107063 |
| 10 | O | 3.414947 | 3.510002 | 3.355412 | 5.317255 | 3.419867 |
| 11 | O | 3.434540 | 3.510770 | 5.319628 | 3.290900 | 3.432980 |
| 12 | O | 3.467343 | 3.374210 | 3.307632 | 3.261377 | 1.183924 |
| | | 6 | 7 | 8 | 9 | 10 |
| 6 | C | 0.000000 | | | | |
| 7 | C | 4.185999 | 0.000000 | | | |
| 8 | O | 3.325418 | 5.329534 | 0.000000 | | |
| 9 | O | 5.324258 | 3.277558 | 4.175701 | 0.000000 | |
| 10 | O | 1.183075 | 5.260537 | 3.800928 | 6.353282 | 0.000000 |
| 11 | O | 5.265862 | 1.182746 | 6.351572 | 3.714807 | 6.283378 |
| 12 | O | 3.405032 | 3.415320 | 3.860102 | 3.811652 | 3.966751 |
| | | 11 | 12 | | | |
| 11 | O | 0.000000 | | | | |
| 12 | O | 3.976691 | 0.000000 | | | |

U1-centroid: 1.991327Å U2-centroid: 1.950286Å

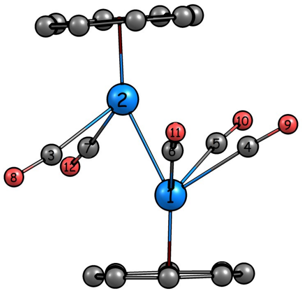


12Q. -60683.321072 +11.7

| | | 1 | 2 | 3 | 4 | 5 |
|---|---|---|---|---|---|---|
| 1 | U | 0.000000 | | | | |
| 2 | U | 2.645889 | 0.000000 | | | |
| 3 | C | 3.151582 | 2.373791 | 0.000000 | | |
| 4 | C | 2.322967 | 3.397895 | 4.830780 | 0.000000 | |
| 5 | C | 2.347093 | 2.894979 | 4.632900 | 2.359454 | 0.000000 |
| 6 | C | 2.341996 | 2.901454 | 3.317270 | 2.358379 | 3.845055 |
| 7 | C | 3.149549 | 2.371732 | 2.861823 | 4.776595 | 3.196317 |
| 8 | O | 4.020409 | 3.535406 | 1.167362 | 5.816183 | 5.714031 |
| 9 | O | 3.493571 | 4.270858 | 5.869355 | 1.171611 | 3.125542 |
| 10 | O | 3.516865 | 3.706897 | 5.653281 | 3.105613 | 1.173394 |
| 11 | O | 3.511999 | 3.718570 | 4.010462 | 3.118717 | 4.879000 |
| 12 | O | 4.012837 | 3.534099 | 3.720123 | 5.745570 | 3.933665 |

| | | 6 | 7 | 8 | 9 | 10 |
|---|---|---|---|---|---|---|
| 6 | C | 0.000000 | | | | |
| 7 | C | 4.663597 | 0.000000 | | | |
| 8 | O | 4.079657 | 3.724434 | 0.000000 | | |
| 9 | O | 3.126590 | 5.802535 | 6.861544 | 0.000000 | |
| 10 | O | 4.868752 | 3.870744 | 6.758869 | 3.531806 | 0.000000 |
| 11 | O | 1.173956 | 5.691860 | 4.623403 | 3.551943 | 5.819760 |
| 12 | O | 5.745756 | 1.167335 | 4.365290 | 6.773279 | 4.446913 |

| | | 11 | 12 |
|---|---|---|---|
| 11 | O | 0.000000 | |
| 12 | O | 6.800663 | 0.000000 |

U1-centroid: 1.967289Å U2-centroid: 1.938954Å

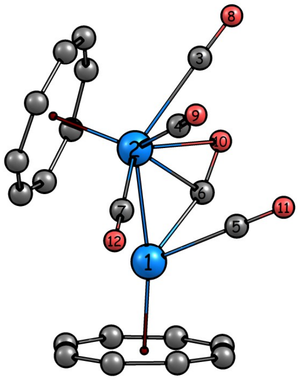


13S. -60683.319811 +12.5

| | | 1 | 2 | 3 | 4 | 5 |
|---|---|---|---|---|---|---|
| 1 | U | 0.000000 | | | | |
| 2 | U | 2.553458 | 0.000000 | | | |
| 3 | C | 4.629130 | 2.433785 | 0.000000 | | |
| 4 | C | 4.050169 | 2.375646 | 2.577006 | 0.000000 | |
| 5 | C | 2.278577 | 2.823806 | 3.773456 | 3.068371 | 0.000000 |
| 6 | C | 2.243569 | 2.468840 | 3.442908 | 4.150693 | 2.366819 |
| 7 | C | 2.705288 | 2.296214 | 4.138966 | 2.244239 | 2.917079 |
| 8 | O | 5.712662 | 3.592655 | 1.159516 | 3.330838 | 4.604352 |
| 9 | O | 5.044299 | 3.540123 | 3.372898 | 1.167120 | 3.819298 |
| 10 | O | 3.372999 | 2.718906 | 2.758330 | 4.198529 | 3.016077 |
| 11 | O | 3.454084 | 3.584684 | 3.810751 | 3.196603 | 1.177961 |
| 12 | O | 3.475466 | 3.481090 | 5.187292 | 2.951837 | 3.613289 |

| | | 6 | 7 | 8 | 9 | 10 |
|---|---|---|---|---|---|---|
| 6 | C | 0.000000 | | | | |
| 7 | C | 3.995146 | 0.000000 | | | |
| 8 | O | 4.334985 | 5.173799 | 0.000000 | | |
| 9 | O | 5.214549 | 2.972910 | 3.835989 | 0.000000 | |
| 10 | O | 1.213177 | 4.622741 | 3.456357 | 5.220070 | 0.000000 |
| 11 | O | 3.104437 | 3.689519 | 4.385721 | 3.680517 | 3.401568 |
| 12 | O | 5.036511 | 1.185497 | 6.152364 | 3.294348 | 5.729710 |

| | | 11 | 12 |
|---|---|---|---|
| 11 | O | 0.000000 | |
| 12 | O | 4.270868 | 0.000000 |

U1-centroid: 1.971560Å U2-centroid: 1.998182Å

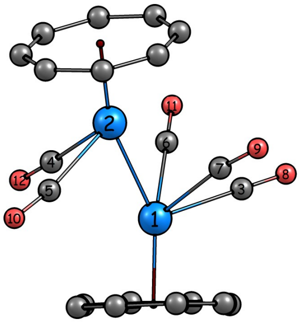


14S. -60683.319183 +12.9

| | | 1 | 2 | 3 | 4 | 5 |
|---|---|---|---|---|---|---|
| 1 | U | 0.000000 | | | | |
| 2 | U | 2.604561 | 0.000000 | | | |
| 3 | C | 2.319843 | 3.870658 | 0.000000 | | |
| 4 | C | 2.843833 | 2.306817 | 4.463819 | 0.000000 | |
| 5 | C | 3.140541 | 2.312773 | 5.259365 | 2.683144 | 0.000000 |
| 6 | C | 2.306439 | 2.719418 | 2.110926 | 2.806815 | 4.424960 |
| 7 | C | 2.299651 | 2.790443 | 2.330270 | 4.414632 | 4.030901 |
| 8 | O | 3.492305 | 4.819615 | 1.172694 | 5.488087 | 6.382126 |
| 9 | O | 3.468185 | 3.635556 | 3.040256 | 5.497189 | 4.929548 |
| 10 | O | 4.056514 | 3.476365 | 6.279844 | 3.522358 | 1.174340 |
| 11 | O | 3.486618 | 3.489054 | 2.845833 | 3.444650 | 5.379278 |
| 12 | O | 3.618839 | 3.481407 | 5.188171 | 1.175380 | 3.493800 |
| | | 6 | 7 | 8 | 9 | 10 |
| 6 | C | 0.000000 | | | | |
| 7 | C | 3.066659 | 0.000000 | | | |
| 8 | O | 2.863286 | 3.083093 | 0.000000 | | |
| 9 | O | 3.993004 | 1.177536 | 3.433506 | 0.000000 | |
| 10 | O | 5.522834 | 5.033162 | 7.423845 | 5.887265 | 0.000000 |
| 11 | O | 1.182551 | 3.997796 | 3.205185 | 4.752533 | 6.485111 |
| 12 | O | 3.522133 | 5.458232 | 6.178017 | 6.575634 | 4.074810 |
| | | 11 | 12 | | | |
| 11 | O | 0.000000 | | | | |
| 12 | O | 3.995298 | 0.000000 | | | |

U1-centroid: 1.987760Å U2-centroid: 1.957373Å

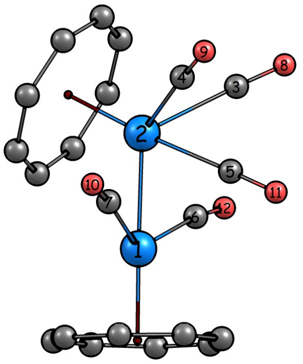


15S. -60683.318868 +13.1

| | | 1 | 2 | 3 | 4 | 5 |
|---|---|---|---|---|---|---|
| 1 | U | 0.000000 | | | | |
| 2 | U | 2.495621 | 0.000000 | | | |
| 3 | C | 4.165690 | 2.358862 | 0.000000 | | |
| 4 | C | 4.155086 | 2.361539 | 2.758943 | 0.000000 | |
| 5 | C | 2.703462 | 2.281938 | 2.440443 | 2.436749 | 0.000000 |
| 6 | C | 2.393590 | 2.760373 | 3.135514 | 4.687859 | 2.979874 |
| 7 | C | 2.384513 | 2.774669 | 4.697477 | 3.133212 | 2.986246 |
| 8 | O | 5.175529 | 3.525238 | 1.167411 | 3.551686 | 3.192836 |
| 9 | O | 5.161939 | 3.527730 | 3.552821 | 1.167263 | 3.188246 |
| 10 | O | 3.550594 | 3.632418 | 5.440145 | 3.336551 | 3.783688 |
| 11 | O | 3.441504 | 3.465514 | 3.217448 | 3.210921 | 1.183784 |
| 12 | O | 3.557527 | 3.629256 | 3.350927 | 5.441905 | 3.778252 |
| | | 6 | 7 | 8 | 9 | 10 |
| 6 | C | 0.000000 | | | | |
| 7 | C | 4.420876 | 0.000000 | | | |
| 8 | O | 3.871364 | 5.748463 | 0.000000 | | |
| 9 | O | 5.739282 | 3.863234 | 4.079762 | 0.000000 | |
| 10 | O | 5.556744 | 1.171426 | 6.445191 | 3.772727 | 0.000000 |
| 11 | O | 3.682889 | 3.683463 | 3.643252 | 3.634240 | 4.350923 |
| 12 | O | 1.171019 | 5.559804 | 3.792805 | 6.447264 | 6.678055 |
| | | 11 | 12 | | | |
| 11 | O | 0.000000 | | | | |
| 12 | O | 4.347170 | 0.000000 | | | |

U1-centroid: 1.967005Å U2-centroid: 1.972373Å

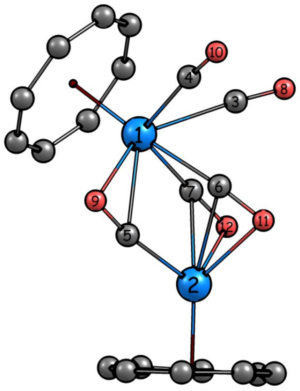


16S. -60683.315321 +15.3

| | | 1 | 2 | 3 | 4 | 5 |
|---|---|---|---|---|---|---|
| 1 | U | 0.000000 | | | | |
| 2 | U | 3.555983 | 0.000000 | | | |
| 3 | C | 2.348282 | 4.194344 | 0.000000 | | |
| 4 | C | 2.487894 | 5.018276 | 2.701610 | 0.000000 | |
| 5 | C | 2.503613 | 2.221488 | 4.179799 | 3.839194 | 0.000000 |
| 6 | C | 2.267583 | 2.443615 | 2.370778 | 2.725113 | 2.332373 |
| 7 | C | 2.212932 | 2.423362 | 2.358566 | 4.103798 | 3.029318 |
| 8 | O | 3.513036 | 4.852200 | 1.166987 | 3.495159 | 5.179744 |
| 9 | O | 2.480511 | 3.443939 | 4.482430 | 3.445227 | 1.232162 |
| 10 | O | 3.639292 | 5.891615 | 3.448150 | 1.155042 | 4.779674 |
| 11 | O | 3.486707 | 2.351863 | 3.115022 | 3.709417 | 3.042054 |
| 12 | O | 3.450109 | 2.332123 | 3.107000 | 5.116667 | 3.772755 |
| | | 6 | 7 | 8 | 9 | 10 |
| 6 | C | 0.000000 | | | | |
| 7 | C | 2.279679 | 0.000000 | | | |
| 8 | O | 3.121761 | 3.107323 | 0.000000 | | |
| 9 | O | 2.932942 | 3.804086 | 5.567499 | 0.000000 | |
| 10 | O | 3.508781 | 5.112606 | 3.962520 | 4.352187 | 0.000000 |
| 11 | O | 1.248101 | 2.892816 | 3.495982 | 3.848902 | 4.254229 |
| 12 | O | 2.901215 | 1.257391 | 3.479708 | 4.755137 | 6.025451 |
| | | 11 | 12 | | | |
| 11 | O | 0.000000 | | | | |
| 12 | O | 2.993123 | 0.000000 | | | |

U1-centroid: 1.932723Å U2-centroid: 2.020345Å

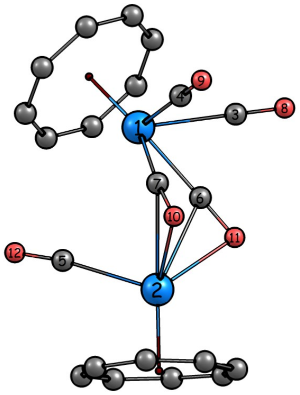


17Q. -60683.315119 +15.5

| | | 1 | 2 | 3 | 4 | 5 |
|---|---|---|---|---|---|---|
| 1 | U | 0.000000 | | | | |
| 2 | U | 3.992134 | 0.000000 | | | |
| 3 | C | 2.323551 | 4.679532 | 0.000000 | | |
| 4 | C | 2.330062 | 4.692571 | 2.508019 | 0.000000 | |
| 5 | C | 3.640634 | 2.418470 | 5.378796 | 5.016947 | 0.000000 |
| 6 | C | 2.214261 | 2.602318 | 2.143447 | 3.128045 | 3.599262 |
| 7 | C | 2.226411 | 2.598921 | 3.087826 | 2.145567 | 3.163753 |
| 8 | O | 3.491976 | 5.378037 | 1.168501 | 3.296580 | 6.390942 |
| 9 | O | 3.498192 | 5.392741 | 3.293673 | 1.168164 | 5.929238 |
| 10 | O | 3.469468 | 2.375010 | 3.941050 | 2.921392 | 3.577249 |
| 11 | O | 3.459447 | 2.361666 | 2.924755 | 3.996801 | 4.108202 |
| 12 | O | 4.161284 | 3.575956 | 6.166720 | 5.662037 | 1.166246 |
| | | 6 | 7 | 8 | 9 | 10 |
| 6 | C | 0.000000 | | | | |
| 7 | C | 2.018289 | 0.000000 | | | |
| 8 | O | 2.912729 | 3.991660 | 0.000000 | | |
| 9 | O | 4.037403 | 2.911140 | 3.785004 | 0.000000 | |
| 10 | O | 2.716697 | 1.249793 | 4.625175 | 3.287395 | 0.000000 |
| 11 | O | 1.251221 | 2.711569 | 3.296419 | 4.694666 | 2.875142 |
| 12 | O | 4.577561 | 4.040391 | 7.243806 | 6.592305 | 4.545971 |
| | | 11 | 12 | | | |
| 11 | O | 0.000000 | | | | |
| 12 | O | 5.208418 | 0.000000 | | | |

U1-centroid: 1.931658Å U2-centroid: 1.966083Å

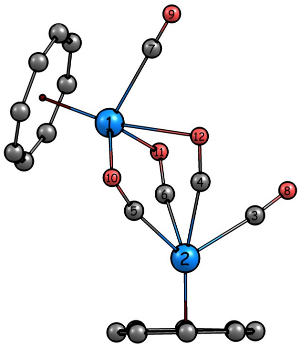


18T. -60683.314905 +15.6

| | | 1 | 2 | 3 | 4 | 5 |
|---|---|---|---|---|---|---|
| 1 | U | 0.000000 | | | | |
| 2 | U | 4.149380 | 0.000000 | | | |
| 3 | C | 4.696414 | 2.357541 | 0.000000 | | |
| 4 | C | 2.969665 | 2.182742 | 2.290881 | 0.000000 | |
| 5 | C | 2.722313 | 2.228556 | 3.852538 | 2.018455 | 0.000000 |
| 6 | C | 2.724648 | 2.197511 | 2.511233 | 2.159017 | 2.644873 |
| 7 | C | 2.477435 | 5.762772 | 5.557099 | 3.810944 | 4.377585 |
| 8 | O | 5.254318 | 3.519561 | 1.165921 | 3.026755 | 4.830089 |
| 9 | O | 3.632500 | 6.693712 | 6.256207 | 4.618802 | 5.366116 |
| 10 | O | 2.356103 | 3.453672 | 4.805175 | 2.658240 | 1.241424 |
| 11 | O | 2.340599 | 3.415741 | 3.201401 | 2.765194 | 3.428867 |
| 12 | O | 2.567421 | 3.389408 | 3.096004 | 1.234061 | 2.680915 |

| | | 6 | 7 | 8 | 9 | 10 |
|---|---|---|---|---|---|---|
| 6 | C | 0.000000 | | | | |
| 7 | C | 4.492531 | 0.000000 | | | |
| 8 | O | 3.288181 | 5.704899 | 0.000000 | | |
| 9 | O | 5.505261 | 1.155087 | 6.244639 | 0.000000 | |
| 10 | O | 3.446249 | 3.730103 | 5.650949 | 4.687517 | 0.000000 |
| 11 | O | 1.243426 | 3.873525 | 3.622128 | 4.862107 | 3.884861 |
| 12 | O | 2.760233 | 2.674773 | 3.473061 | 3.402663 | 2.784984 |

| | | 11 | 12 |
|---|---|---|---|
| 11 | O | 0.000000 | |
| 12 | O | 2.831015 | 0.000000 |

U1-centroid: 1.983140Å U2-centroid: 1.958652Å

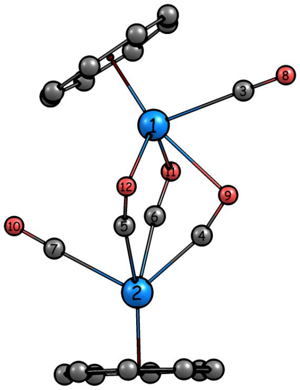


19T. -60683.313982 +16.2

| | | 1 | 2 | 3 | 4 | 5 |
|---|---|---|---|---|---|---|
| 1 | U | 0.000000 | | | | |
| 2 | U | 4.189151 | 0.000000 | | | |
| 3 | C | 2.471202 | 5.760642 | 0.000000 | | |
| 4 | C | 2.981393 | 2.210002 | 3.773251 | 0.000000 | |
| 5 | C | 2.748990 | 2.199286 | 4.456838 | 2.093096 | 0.000000 |
| 6 | C | 2.751521 | 2.201754 | 4.416565 | 2.080826 | 2.605193 |
| 7 | C | 4.060234 | 2.381664 | 6.333957 | 3.835611 | 2.687427 |
| 8 | O | 3.627013 | 6.674681 | 1.155929 | 4.562976 | 5.454623 |
| 9 | O | 2.557067 | 3.412407 | 2.625666 | 1.229942 | 2.722559 |
| 10 | O | 4.454699 | 3.546528 | 6.875618 | 4.849735 | 3.521435 |
| 11 | O | 2.345042 | 3.433416 | 3.793438 | 2.725374 | 3.398682 |
| 12 | O | 2.357823 | 3.427650 | 3.866076 | 2.732128 | 1.242637 |

| | | 6 | 7 | 8 | 9 | 10 |
|---|---|---|---|---|---|---|
| 6 | C | 0.000000 | | | | |
| 7 | C | 2.659155 | 0.000000 | | | |
| 8 | O | 5.402060 | 7.435676 | 0.000000 | | |
| 9 | O | 2.718381 | 4.722377 | 3.341958 | 0.000000 | |
| 10 | O | 3.493059 | 1.165170 | 8.015723 | 5.606786 | 0.000000 |
| 11 | O | 1.245390 | 3.572712 | 4.760979 | 2.832162 | 4.150256 |
| 12 | O | 3.406979 | 3.614804 | 4.849049 | 2.832273 | 4.201009 |

| | | 11 | 12 |
|---|---|---|---|
| 11 | O | 0.000000 | |
| 12 | O | 3.850719 | 0.000000 |

U1-centroid: 1.990220Å U2-centroid: 1.958344Å

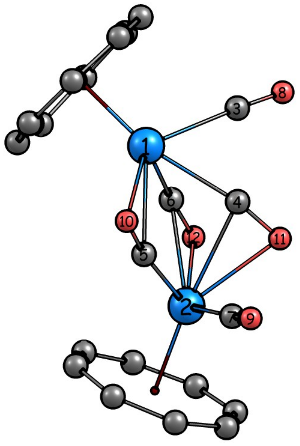


20S. -60683.313658 +16.4

| | | 1 | 2 | 3 | 4 | 5 |
|---|---|---|---|---|---|---|
| 1 | U | 0.000000 | | | | |
| 2 | U | 3.551419 | 0.000000 | | | |
| 3 | C | 2.341789 | 4.309526 | 0.000000 | | |
| 4 | C | 2.257110 | 2.527164 | 2.205514 | 0.000000 | |
| 5 | C | 2.480334 | 2.224093 | 4.189062 | 2.498303 | 0.000000 |
| 6 | C | 2.177676 | 2.489526 | 2.472020 | 2.194403 | 3.047601 |
| 7 | C | 4.262254 | 2.397811 | 5.053297 | 2.855370 | 2.311032 |
| 8 | O | 3.507079 | 5.014886 | 1.166906 | 2.947392 | 5.207056 |
| 9 | O | 4.942510 | 3.557227 | 5.721460 | 3.604429 | 3.047693 |
| 10 | O | 2.522278 | 3.446713 | 4.482893 | 3.109212 | 1.229502 |
| 11 | O | 3.455272 | 2.438388 | 2.949944 | 1.238340 | 3.222257 |
| 12 | O | 3.404264 | 2.383737 | 3.253800 | 2.802963 | 3.772615 |

| | | 6 | 7 | 8 | 9 | 10 |
|---|---|---|---|---|---|---|
| 6 | C | 0.000000 | | | | |
| 7 | C | 4.250564 | 0.000000 | | | |
| 8 | O | 3.253453 | 5.742932 | 0.000000 | | |
| 9 | O | 5.263819 | 1.162114 | 6.382611 | 0.000000 | |
| 10 | O | 3.851167 | 3.022699 | 5.567105 | 3.372257 | 0.000000 |
| 11 | O | 2.759239 | 2.709716 | 3.301847 | 3.498664 | 4.031641 |
| 12 | O | 1.246625 | 4.474280 | 3.702958 | 5.573645 | 4.776331 |

| | | 11 | 12 |
|---|---|---|---|
| 11 | O | 0.000000 | |
| 12 | O | 2.821681 | 0.000000 |

U1-centroid: 1.974078Å U2-centroid: 1.990793Å

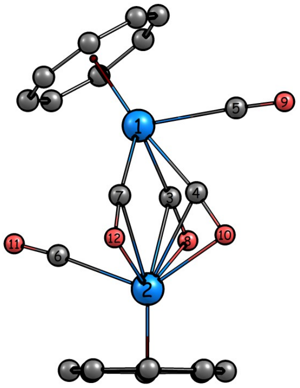


21Q. -60683.313159 +16.7

| | | 1 | 2 | 3 | 4 | 5 |
|---|---|---|---|---|---|---|
| 1 | U | 0.000000 | | | | |
| 2 | U | 3.897674 | 0.000000 | | | |
| 3 | C | 2.272602 | 2.663720 | 0.000000 | | |
| 4 | C | 2.207038 | 2.622523 | 2.051727 | 0.000000 | |
| 5 | C | 2.368806 | 4.797336 | 3.039319 | 2.272611 | 0.000000 |
| 6 | C | 3.701537 | 2.427122 | 3.051441 | 3.862323 | 5.573348 |
| 7 | C | 2.295235 | 2.698363 | 3.055618 | 2.014983 | 3.705818 |
| 8 | O | 3.488458 | 2.468645 | 1.226438 | 2.735694 | 3.920501 |
| 9 | O | 3.531912 | 5.523188 | 3.894286 | 3.031765 | 1.164139 |
| 10 | O | 3.447406 | 2.385790 | 2.737815 | 1.247534 | 3.122350 |
| 11 | O | 4.233903 | 3.587271 | 3.870625 | 4.845133 | 6.331370 |
| 12 | O | 3.504678 | 2.512037 | 3.852178 | 2.702792 | 4.679335 |

| | | 6 | 7 | 8 | 9 | 10 |
|---|---|---|---|---|---|---|
| 6 | C | 0.000000 | | | | |
| 7 | C | 3.029252 | 0.000000 | | | |
| 8 | O | 3.289697 | 3.860257 | 0.000000 | | |
| 9 | O | 6.586983 | 4.666415 | 4.555989 | 0.000000 | |
| 10 | O | 4.326920 | 2.732339 | 2.904810 | 3.528097 | 0.000000 |
| 11 | O | 1.163505 | 3.831757 | 4.183414 | 7.404760 | 5.432344 |
| 12 | O | 3.234544 | 1.221475 | 4.376401 | 5.511000 | 2.908970 |

| | | 11 | 12 |
|---|---|---|---|
| 11 | O | 0.000000 | |
| 12 | O | 4.104541 | 0.000000 |

U1-centroid: 1.959470Å U2-centroid: 1.944680Å

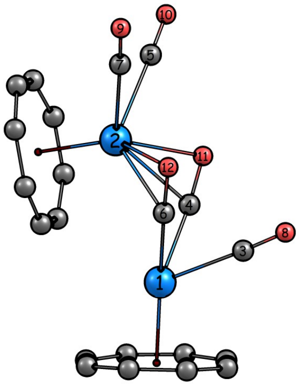


22T. -60683.313010 +16.8

| | | 1 | 2 | 3 | 4 | 5 |
|---|---|---|---|---|---|---|
| 1 | U | 0.000000 | | | | |
| 2 | U | 3.630991 | 0.000000 | | | |
| 3 | C | 2.262392 | 4.169093 | 0.000000 | | |
| 4 | C | 2.165798 | 2.523914 | 2.253521 | 0.000000 | |
| 5 | C | 5.629068 | 2.451638 | 5.525495 | 3.776980 | 0.000000 |
| 6 | C | 2.166993 | 2.524953 | 2.251410 | 2.246990 | 4.517176 |
| 7 | C | 5.636344 | 2.451617 | 5.529039 | 4.515390 | 2.711532 |
| 8 | O | 3.435379 | 4.818054 | 1.173732 | 3.024741 | 5.780526 |
| 9 | O | 6.696152 | 3.608939 | 6.421153 | 5.596443 | 3.490675 |
| 10 | O | 6.686455 | 3.608945 | 6.415854 | 4.720194 | 1.157668 |
| 11 | O | 3.398054 | 2.394018 | 3.009980 | 1.250870 | 2.776167 |
| 12 | O | 3.398898 | 2.392589 | 3.008715 | 2.903043 | 4.005031 |

| | | 6 | 7 | 8 | 9 | 10 |
|---|---|---|---|---|---|---|
| 6 | C | 0.000000 | | | | |
| 7 | C | 3.787163 | 0.000000 | | | |
| 8 | O | 3.022993 | 5.782581 | 0.000000 | | |
| 9 | O | 4.732835 | 1.157698 | 6.539828 | 0.000000 | |
| 10 | O | 5.598066 | 3.491607 | 6.536320 | 4.000630 | 0.000000 |
| 11 | O | 2.904414 | 3.999983 | 3.396586 | 5.016612 | 3.595478 |
| 12 | O | 1.250992 | 2.785726 | 3.395730 | 3.607801 | 5.022842 |

| | | 11 | 12 |
|---|---|---|---|
| 11 | O | 0.000000 | |
| 12 | O | 3.056006 | 0.000000 |

U1-centroid: 1.993001Å U2-centroid: 1.985460Å

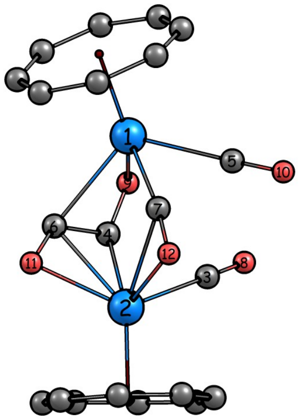


23S. -60683.312334 +17.2

| | | 1 | 2 | 3 | 4 | 5 |
|---|---|---|---|---|---|---|
| 1 | U | 0.000000 | | | | |
| 2 | U | 3.688304 | 0.000000 | | | |
| 3 | C | 3.628479 | 2.357346 | 0.000000 | | |
| 4 | C | 2.500415 | 2.295640 | 2.325600 | 0.000000 | |
| 5 | C | 2.352461 | 3.879825 | 3.012374 | 3.582343 | 0.000000 |
| 6 | C | 2.535032 | 2.450833 | 3.514432 | 1.433053 | 4.173011 |
| 7 | C | 2.199514 | 2.467328 | 3.090350 | 2.955316 | 2.086126 |
| 8 | O | 4.136696 | 3.518570 | 1.164711 | 3.047409 | 3.265032 |
| 9 | O | 2.177061 | 3.507732 | 2.762863 | 1.344523 | 3.357346 |
| 10 | O | 3.521827 | 4.480351 | 3.407786 | 4.537101 | 1.172135 |
| 11 | O | 3.456675 | 2.244732 | 4.095246 | 2.477968 | 4.831313 |
| 12 | O | 3.450838 | 2.355943 | 3.423656 | 3.731665 | 2.924975 |

| | | 6 | 7 | 8 | 9 | 10 |
|---|---|---|---|---|---|---|
| 6 | C | 0.000000 | | | | |
| 7 | C | 2.920297 | 0.000000 | | | |
| 8 | O | 4.391355 | 3.961213 | 0.000000 | | |
| 9 | O | 2.358162 | 3.453227 | 3.021131 | 0.000000 | |
| 10 | O | 5.211200 | 2.844966 | 3.489237 | 4.365476 | 0.000000 |
| 11 | O | 1.301536 | 3.107356 | 5.139943 | 3.603512 | 5.758319 |
| 12 | O | 3.642946 | 1.257973 | 4.396067 | 4.476982 | 3.286754 |

| | | 11 | 12 |
|---|---|---|---|
| 11 | O | 0.000000 | |
| 12 | O | 3.419651 | 0.000000 |

U1-centroid: 1.945409Å U2-centroid: 1.981835Å

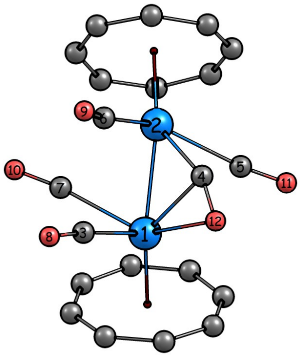

24S. -60683.312237 +17.3

| | | 1 | 2 | 3 | 4 | 5 |
|---|---|---|---|---|---|---|
| 1 | U | 0.000000 | | | | |
| 2 | U | 2.593210 | 0.000000 | | | |
| 3 | C | 2.400800 | 3.633057 | 0.000000 | | |
| 4 | C | 2.473216 | 2.259016 | 3.128973 | 0.000000 | |
| 5 | C | 2.841883 | 2.311866 | 4.779977 | 2.405763 | 0.000000 |
| 6 | C | 3.571022 | 2.436611 | 2.760158 | 2.749261 | 4.387068 |
| 7 | C | 2.306048 | 2.747034 | 2.560477 | 3.874187 | 4.306779 |
| 8 | O | 3.553955 | 4.585006 | 1.161725 | 4.060464 | 5.894685 |
| 9 | O | 4.502141 | 3.587720 | 3.125875 | 3.549051 | 5.450043 |
| 10 | O | 3.479786 | 3.574530 | 3.274379 | 4.907958 | 5.362713 |
| 11 | O | 3.667839 | 3.479717 | 5.683953 | 3.165674 | 1.175619 |
| 12 | O | 2.670796 | 3.378433 | 3.146631 | 1.214257 | 3.059193 |

| | | 6 | 7 | 8 | 9 | 10 |
|---|---|---|---|---|---|---|
| 6 | C | 0.000000 | | | | |
| 7 | C | 3.117430 | 0.000000 | | | |
| 8 | O | 3.160298 | 3.318160 | 0.000000 | | |
| 9 | O | 1.159503 | 3.971152 | 3.110878 | 0.000000 | |
| 10 | O | 3.566606 | 1.180517 | 3.708616 | 4.235535 | 0.000000 |
| 11 | O | 5.461553 | 5.379642 | 6.802147 | 6.474163 | 6.463327 |
| 12 | O | 3.596558 | 4.469301 | 4.010332 | 4.192721 | 5.549647 |

| | | 11 | 12 |
|---|---|---|---|
| 11 | O | 0.000000 | |
| 12 | O | 3.508431 | 0.000000 |

U1-centroid: 1.966520Å U2-centroid: 1.998794Å

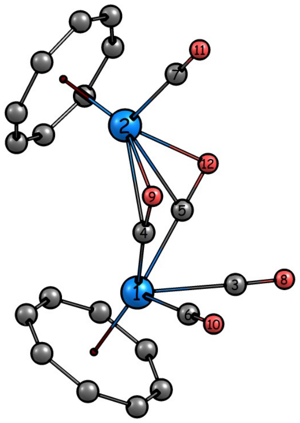

25Q. -60683.311792 +17.5

| | | 1 | 2 | 3 | 4 | 5 |
|---|---|---|---|---|---|---|
| 1 | U | 0.000000 | | | | |
| 2 | U | 4.078320 | 0.000000 | | | |
| 3 | C | 2.331263 | 4.737990 | 0.000000 | | |
| 4 | C | 2.215845 | 2.638176 | 3.150785 | 0.000000 | |
| 5 | C | 2.208346 | 2.612393 | 2.169859 | 1.968160 | 0.000000 |
| 6 | C | 2.332591 | 4.767826 | 2.536764 | 2.203269 | 3.101161 |
| 7 | C | 5.936012 | 2.465963 | 5.992237 | 3.965013 | 4.253337 |
| 8 | O | 3.498803 | 5.405665 | 1.167779 | 4.055727 | 2.933084 |
| 9 | O | 3.463832 | 2.369272 | 4.025799 | 1.252823 | 2.682019 |
| 10 | O | 3.499150 | 5.447831 | 3.327170 | 2.969055 | 4.002291 |
| 11 | O | 6.943966 | 3.623461 | 6.820208 | 4.905388 | 5.256014 |
| 12 | O | 3.466192 | 2.316411 | 2.951089 | 2.681170 | 1.262789 |

| | | 6 | 7 | 8 | 9 | 10 |
|---|---|---|---|---|---|---|
| 6 | C | 0.000000 | | | | |
| 7 | C | 5.725451 | 0.000000 | | | |
| 8 | O | 3.325399 | 6.328024 | 0.000000 | | |
| 9 | O | 2.974088 | 2.938662 | 4.719963 | 0.000000 | |
| 10 | O | 1.166750 | 5.948634 | 3.824428 | 3.347621 | 0.000000 |
| 11 | O | 6.462759 | 1.157534 | 7.031018 | 3.777989 | 6.507250 |
| 12 | O | 3.975009 | 3.458986 | 3.312549 | 2.849828 | 4.657487 |

| | | 11 | 12 |
|---|---|---|---|
| 11 | O | 0.000000 | |
| 12 | O | 4.393865 | 0.000000 |

U1-centroid: 1.930019Å U2-centroid: 1.969870Å

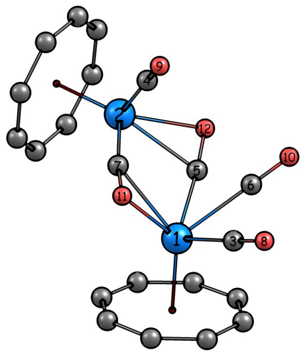

26T. -60683.310580 +18.3

| | | 1 | 2 | 3 | 4 | 5 |
|---|---|---|---|---|---|---|
| 1 | U | 0.000000 | | | | |
| 2 | U | 3.649297 | 0.000000 | | | |
| 3 | C | 2.460910 | 5.362410 | 0.000000 | | |
| 4 | C | 4.130638 | 2.347253 | 4.722016 | 0.000000 | |
| 5 | C | 2.200910 | 2.401359 | 3.854084 | 3.354339 | 0.000000 |
| 6 | C | 2.307026 | 3.950133 | 2.485877 | 3.795154 | 1.920518 |
| 7 | C | 2.428447 | 2.214000 | 3.749654 | 2.275517 | 2.726653 |
| 8 | O | 3.611666 | 6.332495 | 1.156774 | 5.386725 | 4.824772 |
| 9 | O | 4.760904 | 3.513478 | 4.772544 | 1.167691 | 4.245909 |
| 10 | O | 3.488439 | 4.569847 | 3.218816 | 4.140212 | 2.686534 |
| 11 | O | 2.345255 | 3.464037 | 3.049862 | 3.053887 | 3.478669 |
| 12 | O | 3.468039 | 2.300265 | 4.933773 | 3.398887 | 1.270922 |

| | | 6 | 7 | 8 | 9 | 10 |
|---|---|---|---|---|---|---|
| 6 | C | 0.000000 | | | | |
| 7 | C | 3.430769 | 0.000000 | | | |
| 8 | O | 3.201225 | 4.730184 | 0.000000 | | |
| 9 | O | 4.220280 | 3.024451 | 5.214130 | 0.000000 | |
| 10 | O | 1.181416 | 4.345677 | 3.596225 | 4.406754 | 0.000000 |
| 11 | O | 3.609533 | 1.254726 | 3.969314 | 3.418314 | 4.581581 |
| 12 | O | 2.699997 | 3.485503 | 5.799391 | 4.346276 | 2.997317 |

| | | 11 | 12 |
|---|---|---|---|
| 11 | O | 0.000000 | |
| 12 | O | 4.442603 | 0.000000 |

U1-centroid: 1.967441Å U2-centroid: 1.943127Å

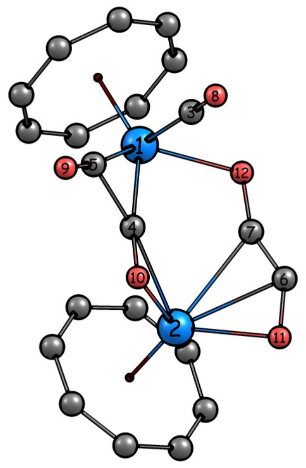

27T. -60683.310058 +18.6

| | | 1 | 2 | 3 | 4 | 5 |
|---|---|---|---|---|---|---|
| 1 | U | 0.000000 | | | | |
| 2 | U | 3.901587 | 0.000000 | | | |
| 3 | C | 2.350477 | 4.820071 | 0.000000 | | |
| 4 | C | 2.192581 | 2.519666 | 2.771993 | 0.000000 | |
| 5 | C | 2.265073 | 4.186993 | 2.318966 | 1.677391 | 0.000000 |
| 6 | C | 4.145417 | 2.497398 | 4.276887 | 3.477149 | 4.910207 |
| 7 | C | 2.951594 | 2.570982 | 3.435357 | 2.838476 | 4.118703 |
| 8 | O | 3.511329 | 5.594904 | 1.161138 | 3.624117 | 3.076217 |
| 9 | O | 3.455148 | 4.902884 | 3.072414 | 2.523304 | 1.197042 |
| 10 | O | 3.473048 | 2.232658 | 3.637784 | 1.290863 | 2.537861 |
| 11 | O | 5.042309 | 2.248386 | 5.183376 | 3.874386 | 5.435869 |
| 12 | O | 2.240736 | 3.583103 | 2.847714 | 3.072444 | 3.900729 |

| | | 6 | 7 | 8 | 9 | 10 |
|---|---|---|---|---|---|---|
| 6 | C | 0.000000 | | | | |
| 7 | C | 1.294014 | 0.000000 | | | |
| 8 | O | 4.803418 | 4.187584 | 0.000000 | | |
| 9 | O | 5.754634 | 5.125703 | 3.479386 | 0.000000 | |
| 10 | O | 3.487674 | 3.322905 | 4.222173 | 2.900998 | 0.000000 |
| 11 | O | 1.291982 | 2.446183 | 5.638949 | 6.116878 | 3.455390 |
| 12 | O | 2.521258 | 1.293162 | 3.720301 | 5.016410 | 3.943227 |

| | | 11 | 12 |
|---|---|---|---|
| 11 | O | 0.000000 | |
| 12 | O | 3.730114 | 0.000000 |

U1-centroid: 1.972762Å U2-centroid: 1.940348Å

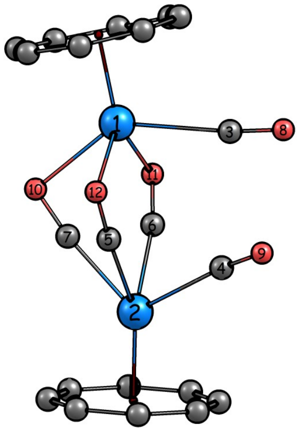

28Q. -60683.310023 +18.7

| | | 1 | 2 | 3 | 4 | 5 |
|---|---|---|---|---|---|---|
| 1 | U | 0.000000 | | | | |
| 2 | U | 4.120362 | 0.000000 | | | |
| 3 | C | 2.462528 | 4.441506 | 0.000000 | | |
| 4 | C | 3.962268 | 2.405062 | 3.033743 | 0.000000 | |
| 5 | C | 2.793146 | 2.253107 | 3.657974 | 2.543927 | 0.000000 |
| 6 | C | 2.812839 | 2.296545 | 3.155587 | 2.996835 | 2.940102 |
| 7 | C | 2.741152 | 2.211193 | 4.257640 | 3.698202 | 1.996503 |
| 8 | O | 3.613994 | 5.110514 | 1.157881 | 3.343696 | 4.578923 |
| 9 | O | 4.496961 | 3.555000 | 3.050290 | 1.161191 | 3.351880 |
| 10 | O | 2.379694 | 3.445661 | 4.483598 | 4.645895 | 2.702869 |
| 11 | O | 2.507953 | 3.501324 | 2.991732 | 3.894426 | 3.719286 |
| 12 | O | 2.412040 | 3.476972 | 3.592114 | 3.327429 | 1.240113 |
| | | 6 | 7 | 8 | 9 | 10 |
| 6 | C | 0.000000 | | | | |
| 7 | C | 2.099801 | 0.000000 | | | |
| 8 | O | 3.917148 | 5.271119 | 0.000000 | | |
| 9 | O | 3.965573 | 4.737152 | 3.037535 | 0.000000 | |
| 10 | O | 2.788938 | 1.241714 | 5.594340 | 5.574916 | 0.000000 |
| 11 | O | 1.222174 | 2.739824 | 3.777279 | 4.688646 | 2.917396 |
| 12 | O | 3.690047 | 2.681411 | 4.588313 | 3.844961 | 2.859317 |
| | | 11 | 12 | | | |
| 11 | O | 0.000000 | | | | |
| 12 | O | 4.154895 | 0.000000 | | | |

U1-centroid: 1.952133Å U2-centroid: 1.952596Å

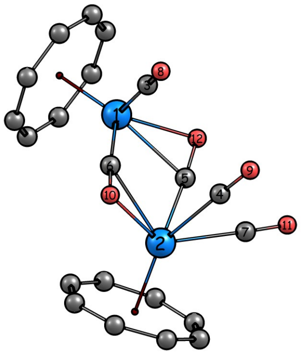

29T. -60683.308446 +19.6

| | | 1 | 2 | 3 | 4 | 5 |
|---|---|---|---|---|---|---|
| 1 | U | 0.000000 | | | | |
| 2 | U | 3.682835 | 0.000000 | | | |
| 3 | C | 2.349249 | 4.227934 | 0.000000 | | |
| 4 | C | 4.161912 | 2.404113 | 3.413376 | 0.000000 | |
| 5 | C | 2.422485 | 2.181492 | 3.413325 | 2.757962 | 0.000000 |
| 6 | C | 2.236644 | 2.498644 | 2.315566 | 3.001368 | 2.761158 |
| 7 | C | 4.526808 | 2.322439 | 4.871200 | 2.358717 | 2.157806 |
| 8 | O | 3.515018 | 4.894576 | 1.167084 | 3.596362 | 4.325290 |
| 9 | O | 4.822015 | 3.563885 | 3.590932 | 1.160860 | 3.598064 |
| 10 | O | 3.467423 | 2.462043 | 3.093733 | 2.861409 | 3.522439 |
| 11 | O | 5.261493 | 3.492926 | 5.536952 | 3.120228 | 2.909855 |
| 12 | O | 2.315107 | 3.442072 | 3.437203 | 3.595073 | 1.262776 |
| | | 6 | 7 | 8 | 9 | 10 |
| 6 | C | 0.000000 | | | | |
| 7 | C | 4.143915 | 0.000000 | | | |
| 8 | O | 3.070528 | 5.437338 | 0.000000 | | |
| 9 | O | 3.826492 | 3.088632 | 3.454494 | 0.000000 | |
| 10 | O | 1.233337 | 4.336300 | 3.483528 | 3.688457 | 0.000000 |
| 11 | O | 5.165919 | 1.172194 | 6.042543 | 3.509441 | 5.422595 |
| 12 | O | 3.509796 | 2.977671 | 4.397951 | 4.162017 | 4.468341 |
| | | 11 | 12 | | | |
| 11 | O | 0.000000 | | | | |
| 12 | O | 3.331165 | 0.000000 | | | |

U1-centroid: 1.973449Å U2-centroid: 1.942899Å

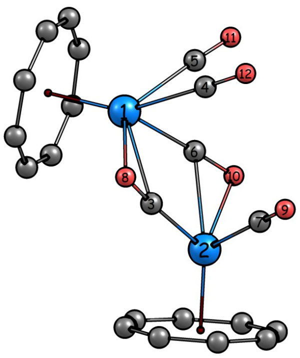

30Q. -60683.308392 +19.7

| | | 1 | 2 | 3 | 4 | 5 |
|---|---|---|---|---|---|---|
| 1 | U | 0.000000 | | | | |
| 2 | U | 3.820412 | 0.000000 | | | |
| 3 | C | 2.611113 | 2.310806 | 0.000000 | | |
| 4 | C | 2.396747 | 4.209101 | 3.063565 | 0.000000 | |
| 5 | C | 2.326791 | 4.567233 | 4.252210 | 2.465258 | 0.000000 |
| 6 | C | 2.183972 | 2.449675 | 2.798213 | 2.805141 | 2.164575 |
| 7 | C | 4.436926 | 2.398883 | 2.630779 | 3.470050 | 4.897761 |
| 8 | O | 2.602306 | 3.516647 | 1.207459 | 2.970327 | 4.508457 |
| 9 | O | 5.131621 | 3.559537 | 3.420385 | 3.681573 | 5.471246 |
| 10 | O | 3.448045 | 2.300327 | 3.569765 | 3.699606 | 2.959176 |
| 11 | O | 3.496212 | 5.257890 | 5.260173 | 3.233620 | 1.170870 |
| 12 | O | 3.558419 | 4.842862 | 3.876476 | 1.163187 | 3.199222 |

| | | 6 | 7 | 8 | 9 | 10 |
|---|---|---|---|---|---|---|
| 6 | C | 0.000000 | | | | |
| 7 | C | 3.446033 | 0.000000 | | | |
| 8 | O | 3.570334 | 3.405395 | 0.000000 | | |
| 9 | O | 4.360963 | 1.161513 | 3.896466 | 0.000000 | |
| 10 | O | 1.264751 | 3.441328 | 4.535659 | 4.402068 | 0.000000 |
| 11 | O | 2.914383 | 5.475883 | 5.587664 | 5.972888 | 3.300108 |
| 12 | O | 3.655672 | 3.557835 | 3.781102 | 3.425991 | 4.297833 |

| | | 11 | 12 |
|---|---|---|---|
| 11 | O | 0.000000 | |
| 12 | O | 3.644794 | 0.000000 |

U1-centroid: 1.943923Å U2-centroid: 1.970899Å

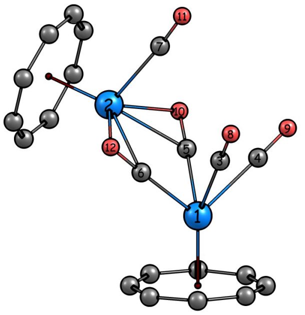

31Q. -60683.308034 +19.9

| | | 1 | 2 | 3 | 4 | 5 |
|---|---|---|---|---|---|---|
| 1 | U | 0.000000 | | | | |
| 2 | U | 3.911059 | 0.000000 | | | |
| 3 | C | 2.332424 | 4.008607 | 0.000000 | | |
| 4 | C | 2.323269 | 4.320650 | 2.347951 | 0.000000 | |
| 5 | C | 2.216728 | 2.557277 | 3.050458 | 2.116336 | 0.000000 |
| 6 | C | 2.261785 | 2.600844 | 2.237524 | 3.580866 | 2.748704 |
| 7 | C | 4.806156 | 2.437583 | 3.652116 | 4.226094 | 3.503768 |
| 8 | O | 3.502266 | 4.544421 | 1.170112 | 3.127330 | 3.965522 |
| 9 | O | 3.495284 | 4.930154 | 3.110723 | 1.172178 | 2.875519 |
| 10 | O | 3.456988 | 2.397527 | 3.961475 | 2.887966 | 1.240628 |
| 11 | O | 5.623911 | 3.594663 | 4.100701 | 4.719099 | 4.417446 |
| 12 | O | 3.486283 | 2.458352 | 3.013109 | 4.572297 | 3.595530 |

| | | 6 | 7 | 8 | 9 | 10 |
|---|---|---|---|---|---|---|
| 6 | C | 0.000000 | | | | |
| 7 | C | 3.507961 | 0.000000 | | | |
| 8 | O | 3.003309 | 3.560391 | 0.000000 | | |
| 9 | O | 4.556806 | 4.381001 | 3.559417 | 0.000000 | |
| 10 | O | 3.611875 | 3.214348 | 4.674846 | 3.237097 | 0.000000 |
| 11 | O | 4.414652 | 1.159242 | 3.693429 | 4.641103 | 4.104220 |
| 12 | O | 1.226047 | 3.215299 | 3.404882 | 5.417464 | 4.171269 |

| | | 11 | 12 |
|---|---|---|---|
| 11 | O | 0.000000 | |
| 12 | O | 4.095523 | 0.000000 |

U1-centroid: 1.926468Å U2-centroid: 1.952471Å

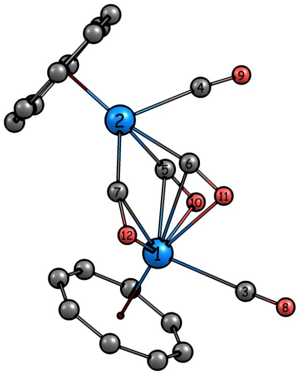

32S. -60683.307887 +20.0

| | | 1 | 2 | 3 | 4 | 5 |
|---|---|---|---|---|---|---|
| 1 | U | 0.000000 | | | | |
| 2 | U | 3.547574 | 0.000000 | | | |
| 3 | C | 2.542412 | 5.443932 | 0.000000 | | |
| 4 | C | 4.415450 | 2.377036 | 5.483130 | 0.000000 | |
| 5 | C | 2.426520 | 2.207522 | 4.147768 | 2.351329 | 0.000000 |
| 6 | C | 2.631410 | 2.202295 | 3.591896 | 2.493072 | 2.197655 |
| 7 | C | 2.431272 | 2.225614 | 4.237129 | 4.006470 | 2.984045 |
| 8 | O | 3.692155 | 6.430835 | 1.150267 | 6.227873 | 5.143226 |
| 9 | O | 5.125113 | 3.537740 | 5.770393 | 1.163722 | 3.106451 |
| 10 | O | 2.312630 | 3.441544 | 3.494268 | 3.129765 | 1.261291 |
| 11 | O | 2.527347 | 3.407582 | 2.550778 | 3.330706 | 2.828967 |
| 12 | O | 2.289966 | 3.464082 | 3.641991 | 4.988768 | 3.762210 |

| | | 6 | 7 | 8 | 9 | 10 |
|---|---|---|---|---|---|---|
| 6 | C | 0.000000 | | | | |
| 7 | C | 2.054627 | 0.000000 | | | |
| 8 | O | 4.430723 | 5.243129 | 0.000000 | | |
| 9 | O | 3.260335 | 5.021822 | 6.355522 | 0.000000 | |
| 10 | O | 2.811413 | 3.771307 | 4.417016 | 3.519224 | 0.000000 |
| 11 | O | 1.231752 | 2.742074 | 3.259251 | 3.789172 | 2.914647 |
| 12 | O | 2.678660 | 1.266095 | 4.586461 | 5.889983 | 4.220006 |

| | | 11 | 12 |
|---|---|---|---|
| 11 | O | 0.000000 | |
| 12 | O | 2.821011 | 0.000000 |

U1-centroid: 2.018412Å U2-centroid: 1.971472Å

Table S5: Mulliken spin densities for the lowest-lying $(COT)_2U_2(CO)_n$ (n = 2, 3, 4, 5) structures computed at the BP86/def2-TZVP/ZORA level of theory

| Structure | U-1 | U-2 | CO-1 | CO-2 | CO-3 | CO-4 | CO-5 | COT-1 | COT-2 | Total |
|---|---|---|---|---|---|---|---|---|---|---|
| $(COT)_2U_2(CO)_2$—01T | 1.077 | 1.102 | -0.031 | -0.029 | | | | -0.058 | -0.061 | 2.000 |
| $(COT)_2U_2(CO)_2$—02T | 1.370 | 0.826 | -0.061 | -0.032 | | | | -0.074 | -0.039 | 2.000 |
| $(COT)_2U_2(CO)_2$—03S | 0.000 | 0.000 | 0.000 | 0.000 | | | | 0.000 | 0.000 | 0.000 |
| $(COT)_2U_2(CO)_2$—04S | 0.000 | 0.000 | 0.000 | 0.000 | | | | 0.000 | 0.000 | 0.000 |
| $(COT)_2U_2(CO)_3$—01T | 0.694 | 1.303 | 0.006 | 0.024 | 0.049 | | | -0.051 | -0.025 | 2.000 |
| $(COT)_2U_2(CO)_3$—02S | 0.000 | 0.000 | 0.000 | 0.000 | 0.000 | | | 0.000 | 0.000 | 0.000 |
| $(COT)_2U_2(CO)_3$—03T | 0.403 | 1.543 | 0.049 | 0.111 | 0.032 | | | -0.053 | -0.085 | 2.000 |
| $(COT)_2U_2(CO)_3$—04S | 0.000 | 0.000 | 0.000 | 0.000 | 0.000 | | | 0.000 | 0.000 | 0.000 |
| $(COT)_2U_2(CO)_3$—05S | 0.000 | 0.000 | 0.000 | 0.000 | 0.000 | | | 0.000 | 0.000 | 0.000 |
| $(COT)_2U_2(CO)_3$—06T | -0.076 | 2.009 | 0.112 | -0.019 | 0.100 | | | -0.102 | -0.025 | 2.000 |
| $(COT)_2U_2(CO)_3$—07Q | 1.814 | 1.859 | 0.175 | 0.068 | 0.223 | | | -0.078 | -0.061 | 4.000 |
| $(COT)_2U_2(CO)_4$—01Q | 2.221 | 2.211 | -0.074 | 0.000 | -0.118 | -0.019 | | -0.085 | -0.135 | 4.000 |
| $(COT)_2U_2(CO)_4$—02Q | 1.899 | 2.195 | 0.001 | 0.067 | 0.001 | 0.062 | | -0.073 | -0.152 | 4.000 |
| $(COT)_2U_2(CO)_4$—03S | 0.000 | 0.000 | 0.000 | 0.000 | 0.000 | 0.000 | | 0.000 | 0.000 | 0.000 |
| $(COT)_2U_2(CO)_4$—04S | 0.000 | 0.000 | 0.000 | 0.000 | 0.000 | 0.000 | | 0.000 | 0.000 | 0.000 |
| $(COT)_2U_2(CO)_4$—05Q | 1.275 | 2.241 | 0.192 | 0.175 | 0.200 | 0.158 | | -0.071 | -0.170 | 4.000 |
| $(COT)_2U_2(CO)_4$—06S | 0.000 | 0.000 | 0.000 | 0.000 | 0.000 | 0.000 | | 0.000 | 0.000 | 0.000 |
| $(COT)_2U_2(CO)_4$—07S | 0.000 | 0.000 | 0.000 | 0.000 | 0.000 | 0.000 | | 0.000 | 0.000 | 0.000 |
| $(COT)_2U_2(CO)_5$—01S | 0.000 | 0.000 | 0.000 | 0.000 | 0.000 | 0.000 | 0.000 | 0.000 | 0.000 | 0.000 |
| $(COT)_2U_2(CO)_5$—02T | 0.463 | 1.489 | 0.031 | 0.033 | 0.035 | 0.039 | 0.033 | -0.116 | -0.007 | 2.000 |
| $(COT)_2U_2(CO)_5$—03T | 0.517 | 1.388 | 0.041 | 0.050 | 0.051 | 0.047 | 0.048 | -0.031 | -0.111 | 2.000 |
| $(COT)_2U_2(CO)_5$—04S | 0.000 | 0.000 | 0.000 | 0.000 | 0.000 | 0.000 | 0.000 | 0.000 | 0.000 | 0.000 |
| $(COT)_2U_2(CO)_5$—05S | 0.000 | 0.000 | 0.000 | 0.000 | 0.000 | 0.000 | 0.000 | 0.000 | 0.000 | 0.000 |
| $(COT)_2U_2(CO)_5$—06S | 0.000 | 0.000 | 0.000 | 0.000 | 0.000 | 0.000 | 0.000 | 0.000 | 0.000 | 0.000 |
| $(COT)_2U_2(CO)_5$—07S | 0.000 | 0.000 | 0.000 | 0.000 | 0.000 | 0.000 | 0.000 | 0.000 | 0.000 | 0.000 |
| $(COT)_2U_2(CO)_5$—08T | 0.444 | 1.461 | 0.061 | 0.060 | 0.039 | 0.054 | 0.040 | -0.009 | -0.124 | 2.000 |
| $(COT)_2U_2(CO)_5$—09T | 1.574 | 0.366 | 0.071 | 0.054 | 0.016 | 0.068 | 0.053 | -0.091 | -0.045 | 2.000 |
| $(COT)_2U_2(CO)_5$—10T | 0.416 | 1.458 | 0.080 | 0.120 | 0.009 | -0.029 | 0.082 | -0.091 | -0.045 | 2.000 |

Table S6: Electronic energies (a.u.) and relative energies (kcal mol$^{-1}$) for the lowest-lying $(COT)_2U_2(CO)_n$ ($n$ = 2–5) structures from single-point M06L/def2-TZVP/ZORA calculations on BP86/def2-TZVP/ZORA optimized geometries. Structures are labeled according to the BP86 ordering.

| Structure | $E$ | ZPC | $E + \text{ZPC}$ | $\Delta E$ |
|---|---|---|---|---|
| $COT_2U_2CO_2$—01T | -60334.298351 | 0.278405 | -60334.019946 | 0.00 |
| $COT_2U_2CO_2$—02T | -60334.290697 | 0.278251 | -60334.012446 | 4.71 |
| $COT_2U_2CO_2$—03S | -60334.281090 | 0.278398 | -60334.002692 | 10.83 |
| $COT_2U_2CO_2$—04S | -60334.278522 | 0.278489 | -60334.000033 | 12.50 |
| $COT_2U_2CO_3$—01T | -60447.647458 | 0.285735 | -60447.361723 | 0.00 |
| $COT_2U_2CO_3$—02S | -60447.640536 | 0.286075 | -60447.354461 | 4.56 |
| $COT_2U_2CO_3$—06T | -60447.638873 | 0.284981 | -60447.353892 | 4.91 |
| $COT_2U_2CO_3$—07Q | -60447.634415 | 0.284509 | -60447.349906 | 7.42 |
| $COT_2U_2CO_3$—03T | -60447.633547 | 0.285355 | -60447.348192 | 8.49 |
| $COT_2U_2CO_3$—04S | -60447.624735 | 0.286073 | -60447.338662 | 14.47 |
| $COT_2U_2CO_3$—05S | -60447.620739 | 0.285533 | -60447.335206 | 16.64 |
| $COT_2U_2CO_4$—02Q | -60561.030644 | 0.297486 | -60560.733158 | 0.00 |
| $COT_2U_2CO_4$—01Q | -60561.029414 | 0.296872 | -60560.732542 | 0.39 |
| $COT_2U_2CO_4$—05Q | -60561.008867 | 0.293344 | -60560.715523 | 11.07 |
| $COT_2U_2CO_4$—03S | -60560.992376 | 0.294210 | -60560.698166 | 21.96 |
| $COT_2U_2CO_4$—04S | -60560.986278 | 0.292905 | -60560.693373 | 24.97 |
| $COT_2U_2CO_4$—06S | -60560.979588 | 0.292933 | -60560.686655 | 29.18 |
| $COT_2U_2CO_4$—07S | -60560.982142 | 0.297951 | -60560.684191 | 30.73 |
| $COT_2U_2CO_5$—02T | -60674.342401 | 0.300576 | -60674.041825 | 0.00 |
| $COT_2U_2CO_5$—01S | -60674.336967 | 0.300463 | -60674.036504 | 3.34 |
| $COT_2U_2CO_5$—03T | -60674.335720 | 0.300175 | -60674.035545 | 3.94 |
| $COT_2U_2CO_5$—04S | -60674.331054 | 0.300353 | -60674.030701 | 6.98 |
| $COT_2U_2CO_5$—09T | -60674.329374 | 0.300490 | -60674.028884 | 8.12 |
| $COT_2U_2CO_5$—08T | -60674.326469 | 0.300134 | -60674.026335 | 9.72 |
| $COT_2U_2CO_5$—05S | -60674.327391 | 0.301442 | -60674.025949 | 9.96 |
| $COT_2U_2CO_5$—10T | -60674.325205 | 0.300104 | -60674.025101 | 10.49 |
| $COT_2U_2CO_5$—07S | -60674.325709 | 0.301435 | -60674.024274 | 11.01 |
| $COT_2U_2CO_5$—06S | -60674.320940 | 0.301023 | -60674.019917 | 13.75 |
